# Selective targeting of mutually exclusive DNA G-quadruplexes: HIV-1 LTR as paradigmatic model

**DOI:** 10.1093/nar/gkaa186

**Published:** 2020-04-13

**Authors:** Martina Tassinari, Michela Zuffo, Matteo Nadai, Valentina Pirota, Adriana Carolina Sevilla Montalvo, Filippo Doria, Mauro Freccero, Sara N Richter

**Affiliations:** 1 Department of Molecular Medicine, University of Padova, via A. Gabelli 63, 35121 Padova, Italy; 2 Department of Chemistry, University of Pavia, v. le Taramelli 10, 27100, Pavia, Italy

## Abstract

Targeting of G-quadruplexes, non-canonical conformations that form in G-rich regions of nucleic acids, has been proposed as a novel therapeutic strategy toward several diseases, including cancer and infections. The unavailability of highly selective molecules targeting a G-quadruplex of choice has hampered relevant applications. Herein, we describe a novel approach, based on naphthalene diimide (NDI)-peptide nucleic acid (PNA) conjugates, taking advantage of the cooperative interaction of the NDI with the G-quadruplex structure and hybridization of the PNA with the flanking region upstream or downstream the targeted G-quadruplex. By biophysical and biomolecular assays, we show that the NDI-PNA conjugates are able to specifically recognize the G-quadruplex of choice within the HIV-1 LTR region, consisting of overlapping and therefore mutually exclusive G-quadruplexes. Additionally, the conjugates can induce and stabilize the least populated G-quadruplex at the expenses of the more stable ones. The general and straightforward design and synthesis, which readily apply to any G4 target of choice, together with both the red-fluorescent emission and the possibility to introduce cellular localization signals, make the novel conjugates available to selectively control G-quadruplex folding over a wide range of applications.

## INTRODUCTION

G-quadruplexes (G4s) are non-canonical nucleic acid (NA) secondary structures formed by guanine-rich single stranded sequences. Deviating from the Watson–Crick base pairing of double stranded DNA, four guanines (Gs) can assemble *via* Hoogsteen-type hydrogen bonds. They thus yield a square planar arrangement, called G-quartet. Two or more quartets can stack on top of each other, constituting the backbone of the G4 structure (1). G4s are highly polymorphic both in terms of strand stoichiometry and strand orientation/topology (2). Additional differentiating structural elements are the loops and flanking regions, respectively connecting the G tracts and lying outside the G4 motif.

Over the past two decades, G4 natural occurrence, significance and roles have emerged (3,4). Putative G4 forming sequences (PQS) are non-randomly distributed across the cell genome and are mainly clustered in ‘hot’ genomic regions, involved in processes key to a number of pathologies (5). These include telomeres (6,7), gene promoters (8,9), DNA replication origins (10,11), open reading frames (12) and untranslated regions (UTRs) (13,14). Their implication in the pathogenesis of cancer (15) and neurodegenerative diseases, such as amyotrophic lateral sclerosis and frontotemporal dementia (16,17), has been extensively described. Moreover, putative G4-forming sequences have been found in mammalian genomes other than the human one (18), as well as in yeasts (19), protozoa (20), bacteria (21,22) and viruses (23). Over the last few years, the presence of G4s in viruses has attracted increasing interest due to their localization in regulatory regions of the genome and subsequent implication in the control of key viral processes (24). We have previously identified and characterized functionally significant G4s in the unique long terminal repeat (LTR) promoter of the human immunodeficiency virus type 1 (HIV-1), the etiological agent of the acquired immune deficiency syndrome (AIDS). Bioinformatic and experimental analysis revealed the presence of three overlapping and thus mutually exclusive G4s, named LTR-II, LTR-III and LTR-IV (25,26) Interestingly, within the full-length LTR G-rich sequence in the presence of physiological concentrations of K^+^, both LTR-II and LTR-III form, with LTR-III being the predominant structure. In contrast, LTR-IV can form only in the presence of G4-ligands and in any case at a lower extent with respect to LTR-III and LTR-II, therefore representing the least stable among LTR G4s (25–27). The LTR G4s act as regulators of viral promoter activity: in physiological conditions formation of LTR-G4s results in decreased viral transcription in cells (25).

Besides the natural presence and therefore relevance of G4s in organisms, G4s have also found a wide range of applications in artificial systems: for example, in the nanotechnology field they have been employed in multiple designs such as G-wires, DNA origami, reconfigurable nanodevices, biosensing nanostructures and nanocarriers for therapeutic purposes [for a recent and comprehensive review see (28)]. Moreover, most of the known aptamers are based on G4-forming oligonucleotides (29,30): some of them are being tested in clinical trials (31), where they show interesting therapeutic and diagnostic applications, while others are effective as biosensors (32).

Such an involvement of G4 structures in diverse human diseases and technological applications propelled the development of G4 ligands (33,34). However, despite the wealth of selective ligands for G4s over other NA conformations, reports on ligand specificity for a relevant G4 with respect to other G4s are lacking. In fact, notwithstanding the considerable G4 polymorphism, ligands usually target the end tetrads and, less diffusedly, the grooves (33), displaying poor specific recognition due to insufficient structural diversity of these sites. To date, the most effective selective G4 targeting has been achieved manly by topological differentiation (parallel vs. anti-parallel and hybrid) (35–38). In this direction, some groups have recently developed different strategies to reach selective binding to specific G4s: G4-binding scaffolds with appended peptide substituents (39), duplex-binders for the recognition of the G4 flanking regions (40,41), DNA molecules that hybridize to the single-stranded flanking regions of an RNA G4 (42).

In this context, we propose a conceptually new approach to achieve high selectivity in the targeting of a specific G4. This is based on the conjugation of two recognition moieties, a peptide nucleic acid (PNA) sequence that hybridizes to the G4 flanking sequence (down- or upstream) to force the system toward the G4 of choice, and a G4 ligand that stacks to the end quartet of the target G4. We validated this new approach on the HIV-1 LTR G4 region. In particular, we were able to induce and stabilize the poorly populated LTR-IV at the expenses of the naturally forming and stable LTR-III. Our results on one hand indicate a successful approach to target one G4s over many others, on the other open up the possibility to use G4s and the control of their folding on a wider range of applications. This goal has been achieved by a general and straightforward chemical strategy which in principle is readily exploitable toward any G4 target of choice.

## MATERIALS AND METHODS

Reagents, solvents and chemicals were purchased from Alfa Aesar (Karlsruhe, Germany) or Sigma-Aldrich (Milan, Italy) and were used as supplied without further purification. PNA monomers were purchased from Panagene (Daejeon, Daejeon, South Korea). Oligonucleotides were purchased from Sigma-Aldrich (Milan, Italy).

PNA and NDI-PNA conjugates were synthesized on a semi-automatic synthesizer (Biotage^®^ Initiator + SP Wave). TLC analysis was carried out on silica gel (Merck 60F-254) with visualization at 254 and 366 nm. For flash column chromatography purification, we used an Isolera ONE Flash Chromatography System (Biotage, Uppsala, Sweden), combined with a tunable wavelength UV/VIS detector. For such purifications we used SNAP 100g columns (KP-SIL Pk 20 by Biotage, flow: 50 ml/min). The solvents used for all the HPLC analyses and purifications were 0.1% trifluoroacetic acid in water and acetonitrile. HPLC analysis was performed using an Agilent system SERIES 1260. The column was XSelectHSS C18 (2.5 μm) (50 × 4.6 mm) (Waters, Mildford, Massachussets). The following analytical method (method 1) was used, flow: 1.4 ml/min; gradient: 95% aqueous, gradually to 40% aqueous over 8 min and then isocratic flow for 4 min. For semi-preparative HPLC purification of NDIs, we used a Waters system combining a Delta 600 PUMP, a 2489 UV/VIS detector and a Fraction Collector III. The preparative column was XSelect CSH Prep Phenyl-Hexyl 5 μm (150 × 30 mm) (Waters, Mildford, Massachussets). The following method was used (method 2), flow: 27 ml/min; gradient: 95% aqueous, gradually to 92% aqueous over 4 min, then gradually to 70% aqueous over 14 min. Preparative reverse phase purification of PNA and NDI-PNA conjugates was carried out using an Agilent Technologies 1260 Infinity preparative HPLC provided with a diode array UV-vis detector. The column was a SunFire C18 OBD (5 μm, 150 × 30 mm). The following method was used (method 3): flow: 30 ml/min; gradient: isocratic flow over 2 min 95% of aqueous solvent, gradually to 60% aqueous over 13 min, then isocratic flow for 1 min (λ detection: 630, 254 and 310 nm). ^1^H-, ^13^C-NMR spectra were recorded on a Bruker ADVANCE 300 MHz. PNA and NDI-PNA conjugates were analysed by ESI-MS (direct injection, MeOH, positive-ion mode, capillary temperature 200°C), using a LCQ ADV MAX ion-trap mass spectrometer, with an ESI ion source.

### Synthesis of tert-butyl 6-aminohexanoate.

Three gram of 6-amino hexanoic acid were gradually dissolved in 15 ml of SOCl_2_, neutralizing the produced HCl with a trap containing 0.6 M aqueous solution of NaOH (Supplementary Scheme S1). The mixture was then stirred for 90 min at r.t. Thionyl chloride was then removed under vacuum. The resulting acyl chloride was submitted to the following step without further purification. It was thus dissolved in 15 ml of *tert*-butanol containing 4 g of sodium monohydrogencarbonate. The mixture was stirred for 2 h. The solvent was subsequently removed under reduced pressure and the resulting solid was dissolved in ethyl acetate. This was washed with three portions of NaOH aqueous solution (1 M), three portions of water and one of brine. The organic phase was then concentrated under reduced pressure to obtain the desired product as a yellow oil (yield = 51%). Comparison of ^1^H-NMR data with those available in the literature (43) confirmed the identity and the purity of tert-butyl 6-aminohexanoate.

### Synthesis of NDI 2

**Scheme 1.**
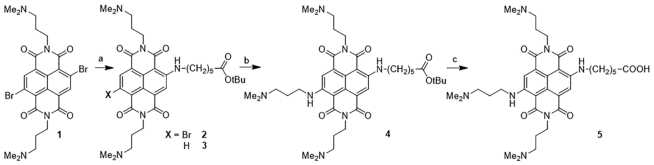
Synthesis of the NDI moiety. (**A**) *tert*-Butyl 6-aminohexanoate, acetonitrile, 60°C, 4.5 h, N_2_; (**B**) *N*,*N*-dimethyl-1,3-propandiamine, 120°C, 5 min, MW; (**C**) dichloromethane, trifluoroacetic acid, triisopropylsilane, r.t., 6 h.

NDI **2** was synthesized using **1** as a starting material (Scheme 1). Its synthesis and characterization are already reported in the literature (44). 0.61 mmol of **1** were dissolved in 150 ml of acetonitrile and 2.5 molar equivalents of tert-butyl 6-aminohexanoate were added. The mixture was heated at 60°C upon stirring for 4.5 h, under nitrogen atmosphere. Upon reaction completion, which was assessed by analytical HPLC (method 1), the solvent was removed under reduced pressure, yielding a mixture of brominated and dehalogenated products (**2** and **3**, Scheme 1). The mixture was usually submitted to the following step without further purification, as the two products cannot be quantitatively separated. Isolation could however be performed by column chromatography (pure chloroform, then gradually to 5% of methanol).

**2:** red solid, purification yield = 11%, ^1^H NMR (300 MHz, CD_3_OD), δ = 7.82 (s, 1H), 7.54 (s, 1H), 3.84 (m, 4H), 3.41 (m, 2H), 2.48 (m, 4H), 2.39 (m, 2H), 2.34 (s, 12H), 1.85 (m, 8H), 1.62 (m, 2H), 1.52 (s, 9H). ^13^C NMR (75 MHz, CD_3_OD), δ = 174.9; 166.1; 162.1; 162.0; 161.5; 152.2; 138.0; 128.4; 127.4; 123.3; 123.2; 121.2; 121.1; 120.6; 99.7; 81.7; 58.4; 58.3; 45.8; 44.4; 40.9; 40.1; 36.6; 31.1; 30.2; 28.8; 27.9; 26.8; 26.7; 26.2.

**3:** red solid, purification yield = 15 %, ^1^H NMR (300 MHz, CDCl_3_), δ = 10.1 (t, 1H, *J* = 5 Hz), 8.62 (d, 1H, *J*_orto_ = 8 Hz), 8.32 (d, 1H, *J*_orto_ = 8 Hz), 8.18 (s, 1H), 4.25 (m, 4H), 3.60 (m, 2H), 2.57 (m, 4H), 2.41 (s, 6H), 2.32 (s, 6H), 2.30 (t, 2H, *J* = 7.5 Hz), 2.00 (m, 4H), 1.86 (m, 2H), 1.71 (m, 2H), 1.57 (m, 2H), 1.46 (s, 9H). ^13^C NMR (75 MHz, CD_3_OD), δ = 174.5; 167.8; 165.1; 164.8; 164.7; 154.1; 133.0; 131.2; 129.6; 127.9; 126.2; 125.2; 121.5; 121.1; 101.5; 81.9; 58.7; 46.7; 46.6;44.9, 40.8; 40.1; 37.0; 31.4; 30.9; 29.8; 28.2; 27.3; 27.2; 26.4.

### Synthesis of NDI 4

A mixture of **2** and **3** (300 mg) was submitted to the second S_N_Ar step, dissolving the crude in 5 ml of *N*,*N*-dimethylpropandiamine. The resulting solution was then heated at 120°C for 5 min (250 psi) with microwaves assistance. The reaction progress was checked by the analytical HPLC (method 1). The diamine was then removed under reduced pressure. Again, the mixture of products (**4** and the remaining by-product **3**), could be directly submitted to the final deprotection step, postponing the purification. However, the desired tetra-substituted product could also be isolated by flash column chromatography (from pure chloroform to 30% methanol supplemented with 1% Et_3_N).

**4:** blue solid, purification yield = 21%, ^1^H NMR (300 MHz, CDCl_3_), δ = 9.33 (t, 1H, *J* = 5 Hz), 9.25 (t, 1H, *J* = 5 Hz), 8.01 (s, 1H), 7.95 (s, 1H), 4.18 (t, 4H, *J* = 7 Hz), 3.52 (m, 2H), 3.46 (m, 2H), 2.45 (m, 6H), 2.28 (m, 20H), 1.97–1.86 (m, 8H), 1.70 (m, 2H), 1.53 (m, 2H), 1.45 (s, 9H). ^13^C NMR (75 MHz, CDCl_3_), δ = 172.7; 165.8; 165.7; 162.7; 162.6; 148.8; 148.7; 125.3; 120.7; 118.0; 117.8; 101.4; 80.0; 57.1; 56.9; 45.4; 45.2; 42.8; 41.1; 38.6; 35.3; 29.0; 28.0; 27.3; 26.5; 25.9; 24.7.

### Synthesis of NDI 5

350 mg of the crude were dissolved in DCM containing 20% trifluoroacetic acid (TFA) and 1% triisopropylsilane (TIS). The solution was stirred at r.t. for 6 h, checking the reaction progress by analytical HPLC (method 1). Upon reaction completion, the solvent was removed under reduced pressure and the final product **5** was obtained by semi-preparative HPLC purification (method 2). Removal of the solvent under reduced pressure yielded the product as a blue solid.

**4·3CF_3_COOH:** blue solid, overall yield = 16%, ^1^H NMR (300 MHz, CD_3_OD), δ = 7.67 (s, 1H), 7.64 (s, 1H); 4.17 (m, 4H), 3.59 (t, 2H, J = 7 Hz), 3.43 (m, 4H); 3.29 (m, 4H); 3.01 (s, 6H); 2.95 (s, 12H); 2.46 (t, 2H, *J* = 7 Hz), 2.28 (m, 2H); 2.17 (m, 4H); 1.87 (m, 4H); 1.31 (m, 2H). ^13^C NMR (75 MHz, CD_3_OD), δ = 177.6; 167.0; 164.1; 150.2; 149.7; 126.4; 126.4; 122.1; 121.8; 118.9; 118.3; 103.0; 102.0; 57.0; 56.9; 44.1; 43.9; 43.8; 41.3; 38.8; 35.1; 30.2; 28.0; 26.1; 25.0.

### PNA synthesis

PNA sequences and conjugates were synthesized on solid support using lysine functionalized Wang resin (0.4–0.6 mmol/g, polystyrene, crosslinked with 1% DVB, 100–200 mesh) (Sigma Aldrich, Milan, Italy) and microwave-assisted Fmoc chemistry. For **NDI-PNA 9** we used a Rink Amide resin (Novabiochem, by Merck, Darmstadt, Germany) (0.37 mmol/g, 100–200 mesh). After deprotection of the resin with 20% (v/v) piperidine in DMF, the first PNA monomer (4 molar equivalents with respect to the resin sites), was added in the presence of 5 equivalents of ethylhydroxyimino cyanoactetate and 5 equivalents of diisopropylcarbodiimide in DMF. After vortexing the mixture for 25 min upon heating (75°C, MW assistance), the supernatant was removed. The reaction time was reduced to 10 min for amino acids. The convenience of a recoupling step was assessed using a Kaiser test kit (Sigma Aldrich, Milan Italy). After that, a capping step was performed using 5% acetic anhydride and 5% of pyridine in DMF. Deprotection of the Fmoc group was performed by treating the resin twice with 20% piperidine in DMF (7 min, r.t.). NDI conjugation was performed directly on the solid support, using HATU as a condensing agent and DIPEA as a base in DMF. Briefly, the NDI was activated for 20 min upon stirring at r.t. in the presence of HATU and DIPEA and then transferred in the reactor. The suspension was vortexed for 2 h at r.t. PNA sequences and conjugates were cleaved from solid support by treatment with a solution of TFA:TIS:double distilled water (ddH_2_O) (95:2.5:2.5, v/v). The treatment was repeated twice (2 h, r.t.). The crude was diluted in acidic water (0.1% TFA) and washed three times with diethyl ether. The desired PNAs were purified by preparative HPLC and characterized by ESI-MS.

**NDI-PNA 1:** m/z found (calculated): 1042.9 (1042.47) [M+4H]^4+^, 1070.9 (1070.97) [M+4H+CF_3_COOH]^4+^, 834.7 (834.38) [M+5H]^5+^, 857.1 (857.14) [M+5H+CF_3_COOH]^5+^, 695.9 (695.31) [M+6H]^6+^, 714.6 (714.31) [M+6H+CF_3_COOH]^6+^, 596.8 (596.13) [M+7H]^7+^, 612.7 (612.41) [M+7H+CF_3_COOH]^7+^.

**NDI-PNA 2:** *m*/*z* found (calculated): 1038.6 (1040.46) [M+4H]^4+^, 1066.7 (1068.96) [M+4H+CF_3_COOH]^4+^, 831.3 (832.58) [M+5H]^5+^, 853.7 (855.37) [M+5H+CF_3_COOH]^5+^, 693.0 (693.98) [M+6H]^6+^.

**NDI-PNA 3:** m/z found (calculated): 1130.9 (1130.52) [M+3H]^3+^, 1182.0 (1182.20) [M+3H+CF_3_COOH]^3+^, 848.5 (848.14) [M+4H]^4+^, 886.7 (886.90) [M+4H+CF_3_COOH]^4+^,915.2 (915.40) [M+4H+2CF_3_COOH]^2+^, 679.1 (678.71) [M+5H]^5+^, 709.5 (709.72) [M+5H+CF_3_COOH]^5+^, 566.2 (566.21) [M+6H]^6+^.

**NDI-PNA 4:** *m*/*z* found (calculated): 1311.0 (1311.12) [M+2H]^2+^, 874.9 (874.42) [M+3H]^3+^, 656.5 (656.06) [M+4H]^4+^, 525.4 (525.05) [M+5H]^5+^, 438.1 (437.71) [M+6H]^6+^.

**NDI-PNA 5:** *m*/**z** found (calculated): 890.8 (890.45) [M+2H]^2+^, 947.4 (947.46) [M+2H+CF_3_COOH]^2+^, 1004.1 (1004.46) [M+2H+2CF_3_COOH]^2+^, 594.4 (594.0) [M+3H]^3+^, 632.1 (632.0) [M+3H+CF_3_COOH]^3+^, 446.2 (445.73) [M+4H]^4+^.

**NDI-PNA 6:** *m*/*z* found (calculated): 1450.4 (1449.32) [M+3H]^3+^, 1088.2 (1087.24) [M+4H]^4+^, 870.8 (869.99) [M+5H]^5+^, 725.9 (725.16) [M+6H]^6+^.

**PNA 7:** *m*/*z* found (calculated): 1234.0 (1233.52) [M+3H]^3+^, 1271.5 (1271.52) [M+3H+CF_3_COOH]^3+^, 1309.5 (1309.53) [M+3H+2CF_3_COOH]^3+^, 1347.6 251 (1347.53) [M+3H+3CF_3_COOH]^3+^, 925.9 (925.40) [M+4H]^4+^, 954.0 (953.89) [M+4H+CF_3_COOH]^4+^.

**NDI-PNA 8:** *m*/*z* found (calculated): 1109.8 (1109.85) [M+3H]^3+^, 832.8 (832.64) [M+4H]^4+^, 666.6 (666.11) [M+5H]^5+^.

**NDI-PNA 9:** *m*/*z* found (calculated): 1713.9 (1713.82) [M+3H]^3+^, 1286.0 (1285.62) [M+4H]^4+^, 1028.9 (1028.70) [M+5H]^5+^, 857.6 (857.41) [M+6H]^6+^, 735.4 (735.07) [M+7H]^7+^, 643.6 (643.3) [M+8H]^8+^.

### Circular dichroism

For CD analysis, oligonucleotides (Supplementary Table S1) were diluted from stock to final concentration (2 × 10^−6^ M) in lithium cacodylate buffer (1 × 10^−2^ M, pH 7.4) and KCl (1 × 10^−1^ M). All samples were annealed by heating at 95°C for 5 min and were left to cool down to r.t. for 4 h. Where indicated, 4 molar equivalents of NDI **6** or NDI-PNA conjugates were added after DNA annealing, and samples were incubated at r.t. for additional 16 h. CD spectra were recorded on a Chirascan-Plus spectropolarimeter (Applied Photophysics, Leatherhead, United Kingdom) equipped with a Peltier temperature controller, using a quartz cell of 5 mm optical path length, over a wavelength range of 230–320 nm. The reported spectrum of each sample represents the average of 2 scans at 20°C and it is baseline corrected for signal contributions due to the buffer. Observed ellipticities were converted to mean residue ellipticity (θ) = deg × cm^2^ × dmol^−1^ (molar ellipticity). For the determination of *T*_m_, spectra were recorded over a temperature range of 20–90°C, with temperature increase steps of 5°C. *T*_m_ values were calculated according to the van’t Hoff equation, applied for a two-state transition from folded to unfolded state, assuming that the heat capacity of the folded and unfolded states are equal (45).

### Taq polymerase stop assay

The DNA primer (Supplementary Table S1) was 5′-end labeled with [γ-^32^P]ATP using T4 polynucleotide kinase (Thermo Scientific, Milan, Italy) at 37°C for 30 min and then purified with Illustra MicroSpin G-25 columns (GE Healthcare, Milan, Italy). The labeled primer (final concentration 7.2 × 10^−8^ M) was annealed to the template (final concentration 3.6 × 10^−8^ M) (Supplementary Table S1) in lithium cacodylate buffer (1 × 10^−2^ M, pH 7.4) in the presence or absence of KCl (1 × 10^−1^ M) by heating at 95°C for 5 min and gradually cooling to r.t. to allow both primer annealing and G4 folding. Where specified, NDI **6** or NDI-PNA conjugates were added at the indicated concentration and incubated overnight. The primer was subsequently extended on the template strand by adding 2 U/reaction of AmpliTaq Gold DNA polymerase (Applied Biosystem, Carlsbad, California, USA) at the indicated temperature for 30 min. Reactions were stopped by ethanol precipitation and primer extension products were separated on a 16 % denaturing gel, and finally visualized by phosphorimaging (Typhoon FLA 9000, GE Healthcare, Milan, Italy). Markers were prepared based on Maxam & Gilbert sequencing by PCR reaction with ^32^P-labeled primer. PCR products were treated with formic acid for 5 min at 25°C and then with piperidine for 30 min at 90°C.

In the competition assay, LTR-III+IV template (Supplementary Table S1) was prepared as reported above. DNA oligonucleotides, designed without the sequence complementary to the primer (competitors) (Supplementary Table S1), were separately incubated at 95°C for 5 min. After that, the template was mixed with increasing amounts of competitor (1–8-fold excess) in the presence of a constant amount of **NDI-PNA 6**. The primer was next extended at 42°C and products were separated on a 16% denaturing gel and visualized by phosphorimaging as previously described.

### UV and fluorescence spectroscopy

NDI **6** and **NDI-PNA 6** were diluted from stock to the final indicated concentration in lithium cacodylate buffer (1 × 10^−2^ M, pH 7.4) and KCl (1 × 10^−1^ M). Absorption spectra were recorded on a Lambda 25 UV-Vis Spectrometer (Perkin Elmer, Milan, Italy) over a wavelength range of 230–800 nm. The emission spectrum of NDI **6** was performed on LS55 Fluorescence Spectrometer (Perkin Elmer, Milan, Italy). All spectrometers were equipped with Peltier temperature controllers. Quartz cuvettes with 10 mm path length were used.

### FRET analysis

In isothermal FRET-based competition assay 5′-FAM and 3′-TAMRA labeled LTR-III+IV and unlabeled NAs (competitors) (Supplementary Table S1) were separately folded for 5 min at 95°C in lithium cacodylate buffer (1 × 10^−2^ M, pH 7.4) supplemented with KCl (1 × 10^−1^ M). After 4 h equilibration at r.t., the labeled NA (2.5 × 10^−7^ M) was mixed with increasing amounts of competitor (0-8 folds excess) in the presence or absence of **NDI-PNA 6** or **PNA 7** (1 × 10^−6^ M). After additional 16 h at r.t., the fluorescence of FAM donor was then measured by Light Cycler II (Roche, Milan, Italy) at 30°C. Data were plotted as a function of the competitor ratio, reporting on the y axis the percentage Δ*F*%, calculated as (Δ*F*_1_/Δ*F*_2_) × 100. Δ*F*_1_ is the difference between the fluorescence of the labeled NA in the presence of both **NDI-PNA 6** or **PNA 7** and competitor and the basal fluorescence of the NA alone. Δ*F*_2_ is the difference in fluorescence measured without competitor.

In FRET melting experiment, the 5′-FAM/3′-TAMRA labeled NAs (Supplementary Table S1) were folded for 5 min at 95°C in lithium cacodylate buffer (1 × 10^−2^ M, pH 7.4) supplemented with KCl (1 × 10^−1^ M). After 4 h equilibration at r.t., NDI **6** or **NDI-PNA 6** (1 × 10^−6^ M) were added where specified. After additional 16 h at r.t., samples were processed in a Light Cycler II (Roche, Milan, Italy). Oligonucleotide melting was monitored by FAM emission in the temperature range of 30–95°C with 1°C/min gradient. Melting profiles were normalized as previously described (46) and *T*_m_ was defined as the temperature corresponding to the 0.5 fraction of the normalized fluorescence.

### EMSA analysis

LTR-III+IV (Supplementary Table S1) was 5′-end labeled with [γ-^32^P]ATP using T4 polynucleotide kinase and, after purification through DNA precipitation or Illustra MicroSpin G-25 columns (GE Healthcare, Milan, Italy), it was resuspended in lithium cacodylate buffer (1 × 10^−2^ M, pH 7.4) and KCl (1 × 10^−1^ M). For strand displacement experiments, labeled LTR-III+IV was annealed to 1.1-fold excess of complementary sequences of different lengths (from 6 to 33 nucleotides) (Supplementary Table S1), for 5 min at 95°C and gradually cooled to room temperature to achieve proper folding. After 30 min, samples were incubated with increasing amounts of **NDI-PNA 6** or **NDI-PNA 7** (0.5–4.0 × 10^−6^ M), at 37°C for 24 h. For competition experiments, ^32^P labeled LTR-III+IV (2.5 × 10^−7^ M) was incubated with **NDI-PNA 9** (2.5 × 10^−7^ M) alone or in the presence of increasing amounts of cold competitor LTR-III+IV or hTel oligonucleotides (0.25–0.5–1–2 × 10^−6^ M) in lithium cacodylate buffer (1 × 10^−2^ M, pH 7.4) in the presence of KCl (1 × 10^−1^ M) at 37°C for 24 h. Samples were loaded on a 16% polyacrylamide native gel in 1× TBE buffer and KCl (1 × 10^−1^ M) and run for 22 h at 60 V. Gels were exposed overnight and visualized by phosphorimaging (Typhoon FLA 9000, GE Healthcare).

### Cell culture

TZM-bI cell line was provided by NIH AIDS Research Program. TZM-bl is a HeLa cell line stably expressing large amounts of CD4 and CCR5 and containing integrated copies of the reporter genes for firefly luciferase and E.coli β-galactosidase under control of the HIV-1 promoter. TZM-bl were grown in Dulbecco's modified Eagle Medium (DMEM) (Gibco, Life Technologies, Monza, Italy) supplemented with 10% heat-inactivated fetal bovine serum (FBS) (Gibco, Life Technologies, Monza, Italy) and maintained as a monolayer in the logarithmic growth phase at 37°C in a 5% CO_2_-controlled humidified atmosphere.

### Cell viability assay

The MTT (3-(4,5-dimethylthiazol 2-yl)-2,5-diphenyltetrazolium bromide, Sigma-Aldrich, Milan, Italy) assay was performed to assess ligand cytotoxicity on TZM-bl cell line. Cells (1 × 10^4^) were plated in 96-well plates and incubated for 24 h. Cells were next treated with increasing concentrations of tested ligands and incubated for an additional 3 or 48 h. Cell viability was evaluated by MTT assay: 10 μl of freshly dissolved solution of MTT (5 mg/ml in PBS) were added to each well, and after 4 h of incubation, MTT crystals were dissolved in solubilization solution (10% sodium dodecyl sulfate (SDS) and 0.01 M HCl). After overnight incubation at 37°C, absorbance was read at 570 nm. The percentage of cell viability was calculated as follows: cell survival = (*A*_well_ − *A*_blank_)/(*A*_control_ − *A*_blank_) × 100, where blank denotes the medium without cells.

### Confocal microscopy

Confocal microscopy was employed to evaluate cell entry of conjugates, exploiting the intrinsic fluorescence of NDI. TZM-bI cells were seeded in 24-well plates (7× 10^4^ cells/well) pretreated with polylysine (1 μg/μl) for 5 min at r.t. and grown overnight. Cells were next treated with the indicated concentration of NDI **6** or NDI-PNA conjugates and, after the indicated time, they were washed five times with PBS to remove the excess of ligand. Cells were fixed with paraformaldehyde (PFA) 2% for 20 min at r.t. and, where indicated, after additional five washes with PBS, nuclear staining was obtained with Nuclear Green LCS1 (Abcam, Cambridge, United Kingdom). For G4 visualization, fixed cells were permeabilized with 0.5% Tween-20 for 40 min at r.t. and blocked in BlockAid (Thermo Scientific, Milan, Italy) for 1 h at 37°C. Subsequently they were incubated with 1 μg/ml anti-G4 antibody 1H6 (kindly provided by P. Lansdorp, European Research Institute for the Biology of Ageing, University of Groeningen, the Netherlands) for 2 h at r.t. and with 1:250 Alexa 488 anti-mouse IgG antibody for 1 h at 37°C. Where specified, fixed cells were treated with DNase (200 units DNase I for 30 min at 37°C) or RNase (40 μg/ml RNaseA for 30 min at 37°C). Fluorescence was evaluated by Nikon A1R confocal laser scanning microscope (Nikon, Tokyo, Japan). For NDI/conjugates (red channel) images were visualized at 561 nm excitation wavelength and 570–620 nm emission range. For 1H6 and cell nuclei (green channel) 488 nm excitation wavelength and 500–550 nm emission range were applied.

## RESULTS

### Targets and conjugates rational design

**Figure 1.**
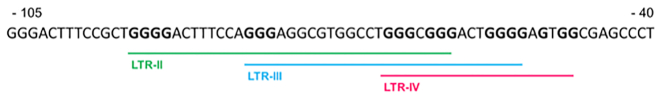
Schematic representation of the sequences that fold into LTR-II, LTR-III and LTR-IV G4s. Guanines involved in G4 structure formation are shown in bold.

The HIV-1 LTR G4 system constitutes a paradigmatic model to target a single G4 among others. In fact, folding of one of the three LTR G4s engages part of the nucleobases common to the other two, thus preventing their concurrent formation (25) (Figure 1). This system highly facilitates the assessment of ligand specificity. In fact, impairment of the LTR G4 folding pattern toward the target of interest would be proof of specificity. Moreover, while LTR-III (and LTR-II to a lesser extent) spontaneously fold in ionic physiological conditions, LTR-IV is very unstable and adopts the G4 conformation *in vitro* exclusively upon ligand binding (25). Therefore, its induction would be an indication of both ligand potency and specificity, to the detriment of the naturally occurring and more stable LTR-III and LTR-II G4s.

**Figure 2.**
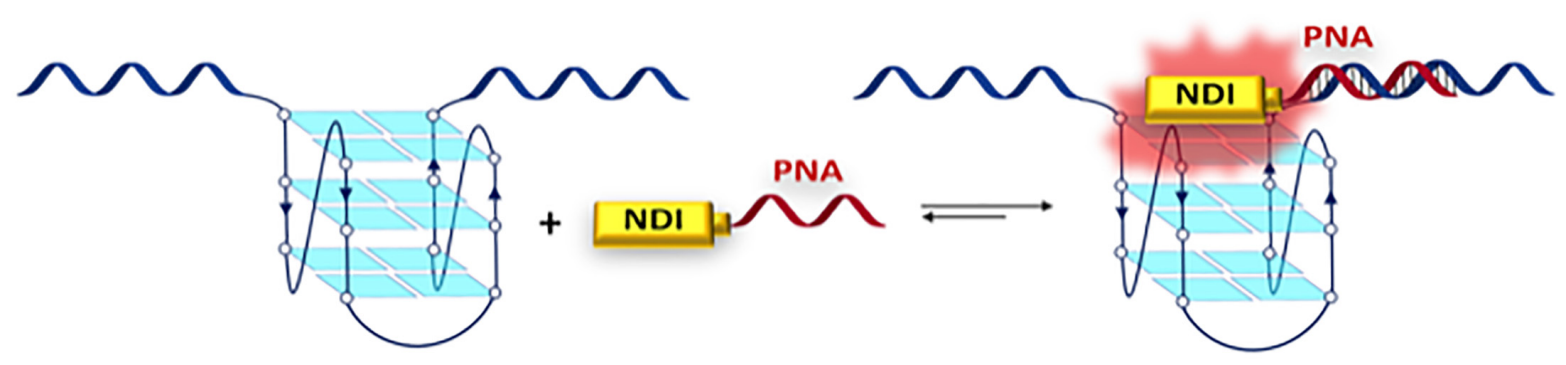
Proposed recognition model for a general G4 structure, based on the cooperative binding of the PNA (in red) to a sequence flanking the G4 of interest (in blue) and contextual interaction of a red fluorescent NDI moiety (in yellow) with the G4 structure.

We thus designed and synthesized a set of conjugates as selective ligands toward the HIV-1 LTR G4s, merging the structural identification of a potent G4 ligand with the antisense recognition of a peptide nucleic acid sequence (PNA)(Figure 2). In particular, a core substituted naphthalene diimide (NDI) was chosen as G4 ligand because of the widely reported potency of NDI derivatives in terms of G4 binding, both *in vitro* (44,47,48) and in more complex systems, such as cells (49,50). Concerning cellular applications, the efficient red emission of the NDI core makes it an ideal tool for tagging and distribution studies. Moreover, the NDI straightforward synthesis facilitates the introduction of the functional groups required for the conjugation chemistry. PNAs are synthetic analogues of nucleic acids, equipped with a neutral aminoethylene glycine backbone. Due to lack of charge repulsion, PNA sequences form highly stable hybrids with complementary DNA single strands and duplexes (51). In addition, the non-natural backbone allows PNAs to escape DNAse and protease degradation (52). Applications of PNAs have been reported in seminal works by Armitage (53) and Ladame (54) and in more recent developments (55–57). PNA sequences can be easily assembled by means of well-established solid phase peptide synthesis (SPPS) (58). The PNA moiety conjugated to the NDI was intended to act as a fingerprint recognition moiety of the DNA sequence proximal to the G4 of interest, driving the binding toward the selected target. The NDI-PNA conjugates are likely to be suitable also for cellular applications: in fact, despite bare PNA sequences are poorly water-soluble and do not easily permeate the cell membrane (58), their conjugation to the polycationic and fluorescent NDI is expected to overcome these issues and allow monitoring cell membrane permeability and distribution (59,60).

In principle, tailored PNA moieties should either hybridize to the flanking regions or the loops of the G4 of interest. Considering that the PNA length is a key factor for specificity, we deemed the flanking regions more suitable as a target. In fact, loops contain a limited number of nucleobases, while the 5′- or 3′-flanking regions enable hybridization with PNAs of any desired length. Moreover, despite LTR-II and LTR-III have long loops (respectively 8 and 11 nucleotides), most G4s only display shorter loops. We thus proceeded with the flanking region option, so as to set up a design with general applicability.

In order to mitigate the constraint arising from contextual hybridization and π-π stacking interactions, we excluded from the hybridization the nucleotide in the flanking region closest to the G4 sequence. We replaced it in the PNA sequence with a lysine residue, to further increase water solubility. With the same aim, an additional lysine residue was added at the opposite end of the PNA sequence. Concerning the NDI moiety, this was designed with a terminal carboxylic group (**5** in Scheme 1) to be conjugated with the free amine of the PNA sequence. In fact, PNA synthesis is usually carried out from the C-terminus to the N-terminus, leaving a free amine at the growing end of the sequence. As up to four substituents can be placed on the NDI, we decided to occupy the three remaining positions with tertiary amine side chains. Besides increasing water-solubility upon protonation, this is also reported to enhance affinity for the G4 structure (47).

### Synthesis of the NDI-PNA conjugates

The NDI for the final conjugation was synthesized starting from the 2,6-dibromo functionalized precursor **1** (Scheme 1), whose synthesis is reported in the literature (61). This was subjected to two subsequent nucleophilic aromatic substitution (S_N_Ar) steps. In particular, the side chain containing the protected carboxylic acid group was inserted first. Reaction with the bare acid proved inefficient, due to the scarce reactivity of the dominant zwitterionic form. The intermediate **2** was obtained in a mixture with the partially de-halogenated derivative **3**, due to the competing reduction favored by the amine reactant. The second core substituent was inserted using the nucleophile as a solvent, through a microwave-assisted protocol. Product **4** was finally recovered and deprotected under acidic conditions, quantitatively yielding the NDI **5**.

The PNA sequences were synthesized according to well-established SPPS procedures, using the standard Fmoc chemistry in DMF. The NDI conjugation was performed directly on the resin. In order to prevent the compound degradation, the coupling was performed at r.t. with HATU as condensing agent. Final cleavage and purification by reverse phase preparative HPLC yielded the desired conjugates. The list of the conjugates synthesized according to this protocol is reported in Table 1. Conjugates differed both in terms of PNA length, HIV-1 LTR G4 and 5′- or 3′-flanking region targets.

**Table 1.** NDI-PNA conjugates and the reference PNA sequence (**PNA 7**) synthesized for the present study

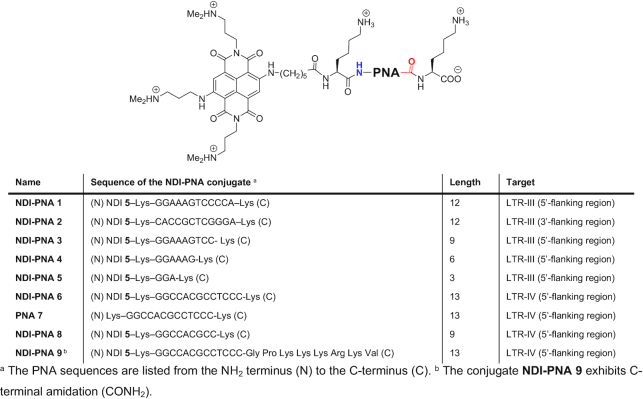

### Design optimization: targeting of LTR-III

**Figure 3.**
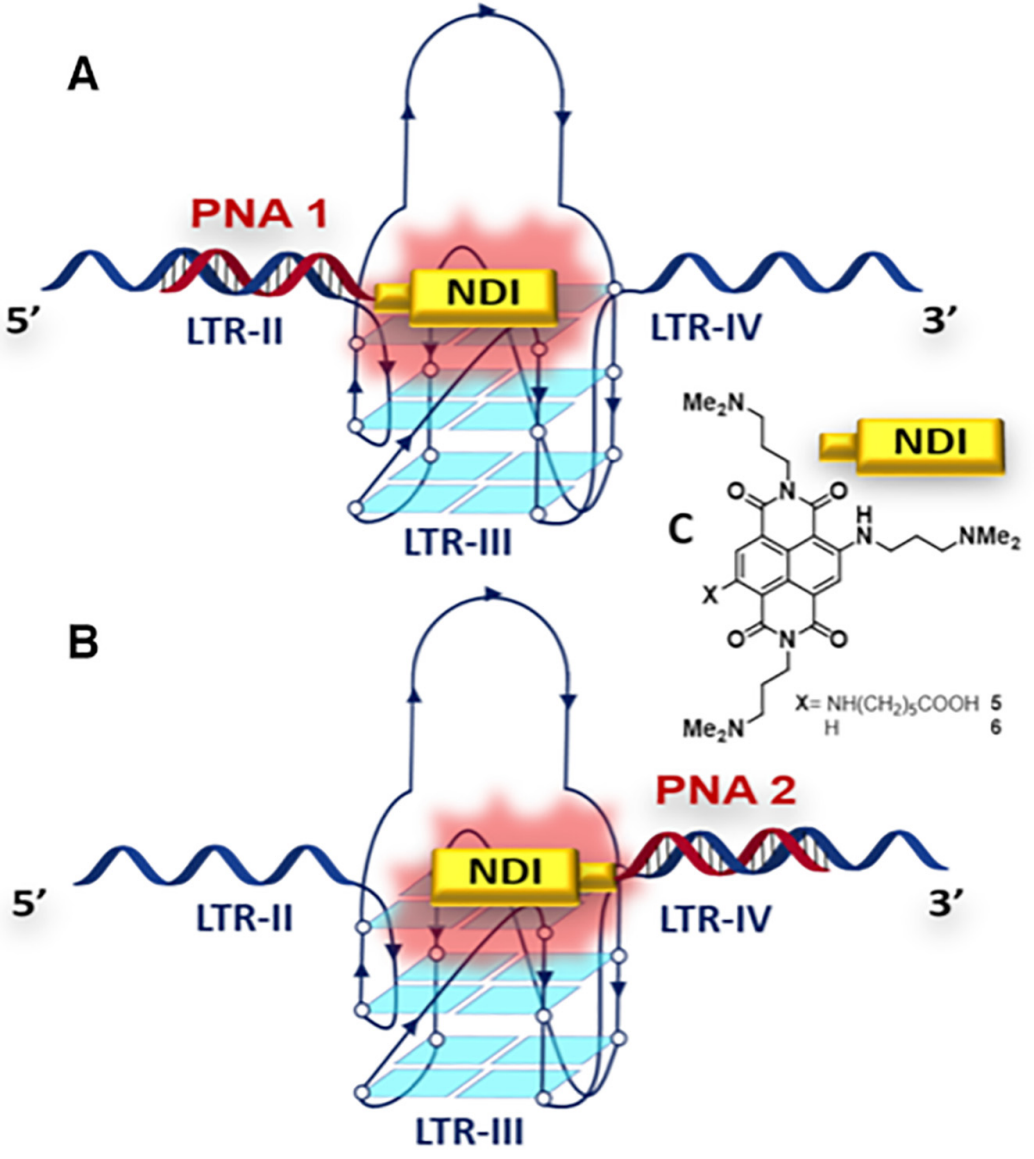
Conjugates **NDI-PNA 1** and **NDI-PNA 2** binding the full G-rich LTR sequence (here called LTR-II+III+IV), according to (**A**) anti-parallel and (**B**) parallel binding modes; (**C**) structures of both conjugated NDI **5** and control NDI **6**.

The conjugates were initially optimized against LTR-III G4, which is the LTR G4 that folds spontaneously under physiological conditions (25,26). Its convenient central position within the LTR G-rich sequence allows the PNA moiety to extend either toward the 5′ or 3′ direction and thus to complement sequences involved in either LTR-II or LTR-IV folding, respectively. We synthesized **NDI-PNA 1** and **2**, two conjugates formed by NDI **5** and a PNA strand complementary to the LTR-III 5′- and 3′-flanking regions, respectively (Table 1). **NDI-PNA 1** comprises 12 monomers that hybridize with all the nucleotides participating in LTR-II folding and lying outside the LTR-III sequence (Figure 3A). In **NDI-PNA 2** we maintained the same PNA length for a meaningful comparison, with the PNA complementing four nucleotides contained in LTR-IV and lying outside LTR-III G4, and eight additional nucleotides in the 3′ direction not involved in G4 formation. Since NDI **5** was conjugated to the PNA N-terminus for synthetic reasons (Table 1), the two conjugates hybridize to the target DNA sequence with opposite orientations. According to the conventional definition, the orientation adopted by the 5′-flanking region - **NDI-PNA 1** hybrid (DNA 5′-end facing the PNA C-terminus and DNA 3′-end facing the PNA N-terminus) is anti-parallel (Figure 3A), whereas that expected for the 3′-flanking region - **NDI-PNA 2** hybrid (the opposite one) is parallel (Figure 3B). According to literature data, the two orientations produce similar stability, with a slightly higher melting temperature for the anti-parallel structure (62).

A control NDI (**6**, Figure 3C), whose synthesis and high affinity for G4s have already been reported in the literature (59), was used to compare the effect of the combined PNA and NDI moieties in the conjugate with the less specific interaction provided by the NDI alone.

Binding of the conjugates to the target oligonucleotides, preventively annealed in K^+^ containing buffer, was first assessed by circular dichroism (CD) experiments. To test **NDI-PNA 1**, we used a sequence encompassing all LTR G4s (LTR-II+III+IV; Supplementary Table S1). For **NDI-PNA 2** we used an extended version of the sequence (LTR-II+III+IV+3′; Supplementary Table S1), containing the relevant 3′-flanking region with 8 additional bases targeted by the PNA moiety. The CD spectra of both LTR-II+III+IV and LTR-II+III+IV+3′ sequences are complex and cannot be ascribed to a single topology (Supplementary Figures S1A and B, magenta lines). **NDI-PNA** 1 had the highest impact on these two sequences, since it induced them to fold into a prevalent parallel conformation (Supplementary Figures S1A and B, red lines); in contrast, in the presence of control NDI **6** (Supplementary Figures S1A and B, blue lines) and **NDI-PNA 2** (Supplementary Figure S1B, green line) both sequences maintained the multiple conformation signature,suggesting a less specific interaction with respect to the **NDI-PNA 1** conjugate. These data indicate that the interaction of **NDI-PNA 1** with the target, and thus the anti-parallel PNA design, is more effective (Figure 3). Next, we sought to evaluate whether the presence of an encumbering PNA sequence affected the interaction of the NDI moiety with the G-quartet of a control G4 sequence. We thus assessed the interaction of the two conjugates with the telomeric G4 (hTel; 21 nucleotides long, Supplementary Table S1), whose flanking regions cannot hybridize with the PNA moiety of the conjugates. Both conjugates and NDI **6** barely increased the molar ellipticity and modified the hybrid-type signature of hTel to a similar extent (Supplementary Figure S1C), suggesting that the only active moiety against hTel G4 is the NDI core. In fact, while **NDI-PNA 1** stabilized the LTR-II+III+IV sequence to a higher extent compared to NDI **6** (Supplementary Figure S2), in contrast, **NDI-PNA 1** stabilization toward hTel was similar to that of control NDI **6**, thus corroborating that the effect on hTel was solely to be ascribed to the NDI moiety (Supplementary Figure S3).

**Figure 4.**
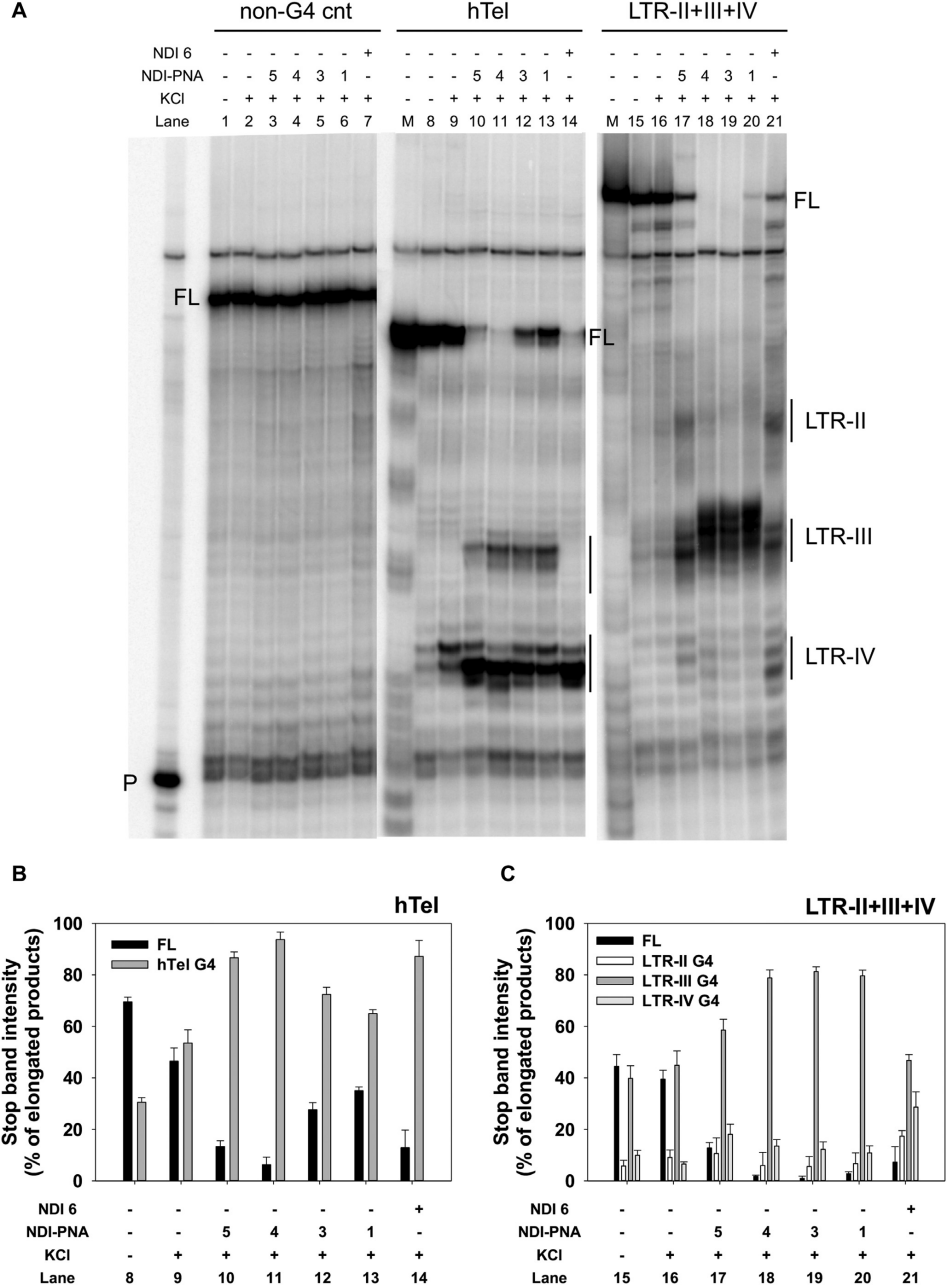
Taq polymerase stop assay on LTR-II+III+IV and control hTel templates. (**A**) LTR-II+III+IV and hTel templates were amplified by Taq polymerase at 42°C in the absence (lanes 8 and 15) and presence of 1 × 10^−1^ M KCl, alone (lanes 9 and 16) or with 4 × 10^−7^ M of **NDI-PNA 5** (lanes 10 and 17), **NDI-PNA 4** (lanes 11 and 18), **NDI-PNA 3** (lanes 12 and 19), **NDI-PNA 1** (lanes 13 and 20), or NDI **6** (lanes 14 and 21). A template (non-G4 cnt) made of a scrambled sequence unable to fold into G4 was also used as negative control (lanes 1–7). Lane P: unreacted labeled primer. Lane M: ladder of markers obtained by the Maxam and Gilbert sequencing carried out on the amplified strand complementary to the template strand. Vertical bars indicate G4-specific Taq polymerase stop sites. The three gel portions derive from a single gel run. (**B**) Quantification of lanes 8–14 shown in panel A. Quantification of stop bands corresponding to hTel G4 and of the full-length amplification product (FL) is shown. (**C**) Quantification of lanes 15–21 shown in panel A. Quantification of stop bands corresponding to LTR-II, LTR-III, LTR-IV G4s and of the full-length amplification product (FL) is shown.

To identify the LTR G4 preferentially bound by **NDI-PNA 1**, we performed a Taq polymerase stop assay on the full-length LTR sequence (Figure 4A). NDI **6** was used as control compound, while hTel G4 and a NA unable to fold into G4 were used as control sequences (Supplementary Table S1). **NDI-PNA 1** induced a unique marked stop corresponding to LTR-III G4 on the LTR-II+III+IV template (Figure 4A, lane 20). In contrast, NDI **6** induced stops corresponding to all three G4s (Figure 4A, lane 21). On the hTel template, NDI **6** almost entirely paused the enzyme at the first encountered G-tract, indicating tight binding with the target (Figure 4A, lane 14). On the contrary, **NDI-PNA 1** induced milder stops at both the first and second G-tracts, suggesting less efficient interaction with the hTel G4 as also indicated by the higher amplification of the full-length products (Figure 4A, lane 13). Neither **NDI-PNA 1** nor NDI **6** displayed any effect on the non-G4 forming template (Figure 4A, lanes 6–7). These data clearly advocate for specific binding of **NDI-PNA 1** to its designated LTR-III target.

To assess the PNA length role in dictating target specificity, we employed conjugates **NDI-PNA 3**, **4** and **5**, with shorter PNAs (9-, 6- and 3-monomer long, respectively, Table 1) with respect to **NDI-PNA 1**. **NDI-PNA 5** induced additional stops corresponding to LTR-II and LTR-IV G4s on the LTR-II+III+IV template (Figure 4A, lanes 17), while **NDI-PNA 3** and **4** maintained an activity similar to that of **NDI-PNA 1** (Figure 4A, lanes 18–19). In good agreement, bands quantification showed that the stop induced at LTR-III G4 depended on the PNA length (Figure 4C). On the hTel sequence, the shortest PNA sequence displayed the most similar behavior to that of NDI **6**, whereas the longer sequences mimicked **NDI-PNA 1** behavior (Figure 4A, lanes 10–14, and 4B). Finally, no stops were observed on the control template (Figure 4A, lanes 3–7). Altogether, these data indicate that NDI-PNA specificity depends on the PNA length, with a minimum of 6 monomers being necessary to observe a selectivity effect.

### Specificity assessment: targeting of LTR-IV

The next step in conjugate development was to target LTR-IV G4. This G4 does not naturally fold within the LTR sequence in physiological ionic conditions and can be induced through the binding of G4 ligands (25,26). Only the conjugate hybridizing with the 5′-flanking region was synthesized in this case. On one side, this allowed the engagement of the bases involved in LTR-III and LTR-II folding. On the other side, this design enabled a pseudo anti-parallel hybridization, which showed to be more effective in LTR-III targeting. The conjugate contained a 13 monomer-long PNA (**NDI-PNA 6**, Table 1) able to hybridize with the entire LTR-III sequence lying outside LTR-IV. To assess the role of the PNA moiety, the unconjugated PNA (**PNA 7**) was also synthesized. These compounds were assayed on the shorter LTR-III+IV sequence (Supplementary Table S1), which contains all the targeted bases and folds exclusively into LTR-III or LTR-IV G4s, yielding a less complex system than the previously utilized sequences.

The LTR-III+IV G4 CD spectrum showed a mixture of conformations (Supplementary Figure S4A, magenta line) which became a clear parallel G4 structure in the presence of **NDI-PNA 6** (Supplementary Figure S4A, red line). No induced CD was observed at the longer **NDI-PNA 6** absorption wavelengths (Supplementary Figure S5). **PNA 7** had a similar but less marked effect (Supplementary Figure S4A, green line), while incubation with control NDI **6** only slightly modified LTR-III+IV G4 positive peaks (Supplementary Figure S4A, blue line). These data indicate that PNA hybridization allows formation of a parallel G4, which is stabilized by the NDI moiety of the conjugate, whereas the NDI alone does not preferentially stabilize any G4 conformation. Both **NDI-PNA 6** and **PNA 7** had minor effects on the CD spectrum of the hTel sequence, whereas NDI **6** prompted its transition to an anti-parallel conformation (Supplementary Figure S4B). This suggests that the PNA moiety prevents an effective interaction of the NDI moiety with the hTel sequence.

These indications were completed by CD thermal unfolding experiments. **NDI-PNA 6** strongly stabilized LTR-III+IV G4 parallel conformation (*T*_m_ > 90°C and Δ*T*_m_ > 25°C, corresponding to the maximum measurable variation under these conditions, Supplementary Figure S6B-C), while **PNA 7** did not produce any changes in the G4 melting temperature (Δ*T*_m_ < 1°C, Supplementary Figure S6F-G). Similar to LTR-II+III+IV, LTR-III+IV was also stabilized by NDI **6** with multiphasic melting curves (Supplementary Figure S6D-E). These data indicate that the presence of the PNA forces the LTR-III+IV G4 to fold into the parallel conformation, which is then highly stabilized by the NDI moiety. As for hTel G4, in the presence of **NDI-PNA 6**, when the temperature was increased, the parallel conformation was induced and stabilized by the NDI moiety (Supplementary Figure S7B-C), mirroring the behavior of the control NDI **6** (Supplementary Figure S7D-E). Finally, no stabilization was detected upon addition of **PNA 7** (Δ*T*_m_ = 1.2 ± 0.4°C) (Supplementary Figure S7F-G). Altogether, these results indicate that the PNA region provides specific stability toward the target LTR-II+III G4 at room temperature (r.t.). Upon temperature increase, however, PNA hybridization is likely lost and the unspecific effect of the NDI moiety becomes prevalent.

CD experiments on LTR-III+IV were also performed at 1:2 and 1:1 DNA/**NDI-PNA 6** molar ratios. CD spectra of LTR-III+IV oligonucleotide recorded at 1:2 and 1:4 DNA/NDI-PNA ratios were almost identical (Supplementary Figure S8, compare magenta and red lines), indicating that 2-fold excess of **NDI-PNA 6** were enough to induce a clear parallel G4. At 1:1 DNA/ NDI-PNA, the LTR-III+IV template switched from a mixed to a prevalent parallel conformation in the absence vs. the presence of ligand (Supplementary Figure S8, blue line). At all molar ratios, T_m_ > 90°C was observed (Supplementary Figure S9C-E-G), indicating that the 1:1 DNA/NDI-PNA molar ratio was sufficient to induce high G4 stabilization.

**Figure 5.**
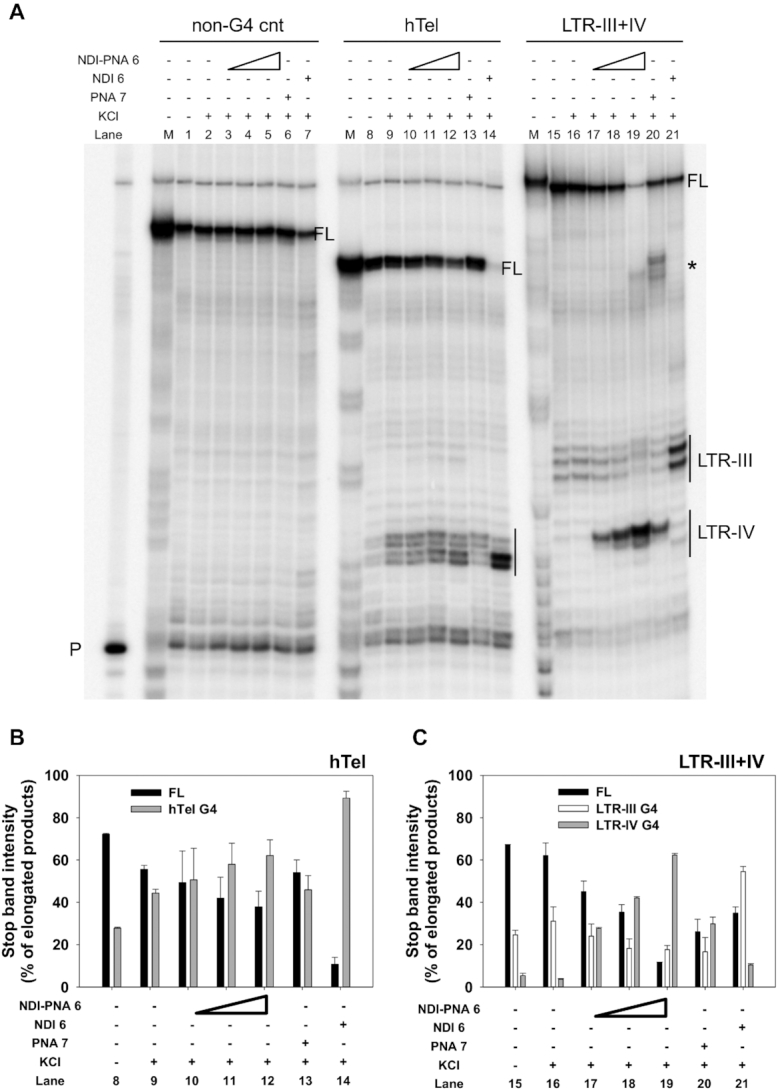
Taq polymerase stop assay on LTR-III+IV and control hTel templates. (**A**) LTR-III+IV and hTel templates were amplified by Taq polymerase at 42°C in the absence (lanes 8 and 15) and presence of 1 × 10^−1^ M KCl, alone (lanes 9 and 16) or with increasing amounts (1 × 10^−7^, 2 × 10^−7^ and 4 × 10^−7^ M) of **NDI-PNA 6** (lanes 10–12 and 17–19), 4 × 10^−7^ M of **PNA 7** (lanes 13 and 20) or 4 × 10^−7^ M of NDI **6** (lanes 14 and 21). A template (non-G4 cnt) made of a scrambled sequence unable to fold into G4 was also used as negative control (lanes 1–7). Lane P: unreacted labeled primer. Lane M: ladder of markers obtained by the Maxam and Gilbert sequencing carried out on the amplified strand complementary to the template strand. Vertical bars indicate G4-specific Taq polymerase stop sites. * indicates the stop site corresponding to the binding of the PNA to its complementary sequence on the LTR-III+IV template. (**B**) Quantification of lanes 8–14 shown in panel A. Quantification of stop bands corresponding to hTel G4 and of the full-length amplification product (FL) is shown. (**C**) Quantification of lanes 15–21 shown in panel A. Quantification of stop bands corresponding to LTR-III, LTR-IV G4s and of the full-length amplification product (FL) is shown.

The Taq polymerase stop assay was next performed on the LTR-III+IV, hTel and non-G4 forming templates. LTR-III formed the most stable G4 when embedded in the LTR-III+IV template (Figure 5A, lane 16), which was further greatly stabilized by NDI **6** (Figure 5A, lane 21). **PNA 7** induced formation of an intense stop site corresponding to LTR-IV, and concurrently decreased the LTR-III stop site (Figure 5A, lane 20). These data confirm that the PNA, by binding to its DNA complementary region, prevents formation of LTR-III and blocks Taq polymerase progression. **NDI-PNA 6** stabilized LTR-IV G4 in a concentration-dependent manner and to a higher extent compared to **PNA 7** (Figure 5A, lanes 17–19 and comparison between lanes 19 and 20, and Figure 5C). Moreover, the G4 stop induced by **NDI-PNA 6** was more intense than that observed with NDI **6** (Figure 5A, lane 19 versus lane 21, and Figure 5C): at the LTR-IV and LTR-III G4 stop sites, stop bands were 62.2 ± 0.9% and 17.7 ± 1.9% of the elongated products, respectively. On the hTel template, stabilization of the G4 in the presence of both **NDI-PNA 6** and **PNA 7** was only slightly higher than that obtained in the presence of KCl alone (Figure 5A, lanes 9–13, and Figure 5B). In contrast, NDI **6** induced a G4 stop that accounted for >90% of the total elongated products (Figure 5A, lane 14, and Figure 5B).

**Figure 6.**
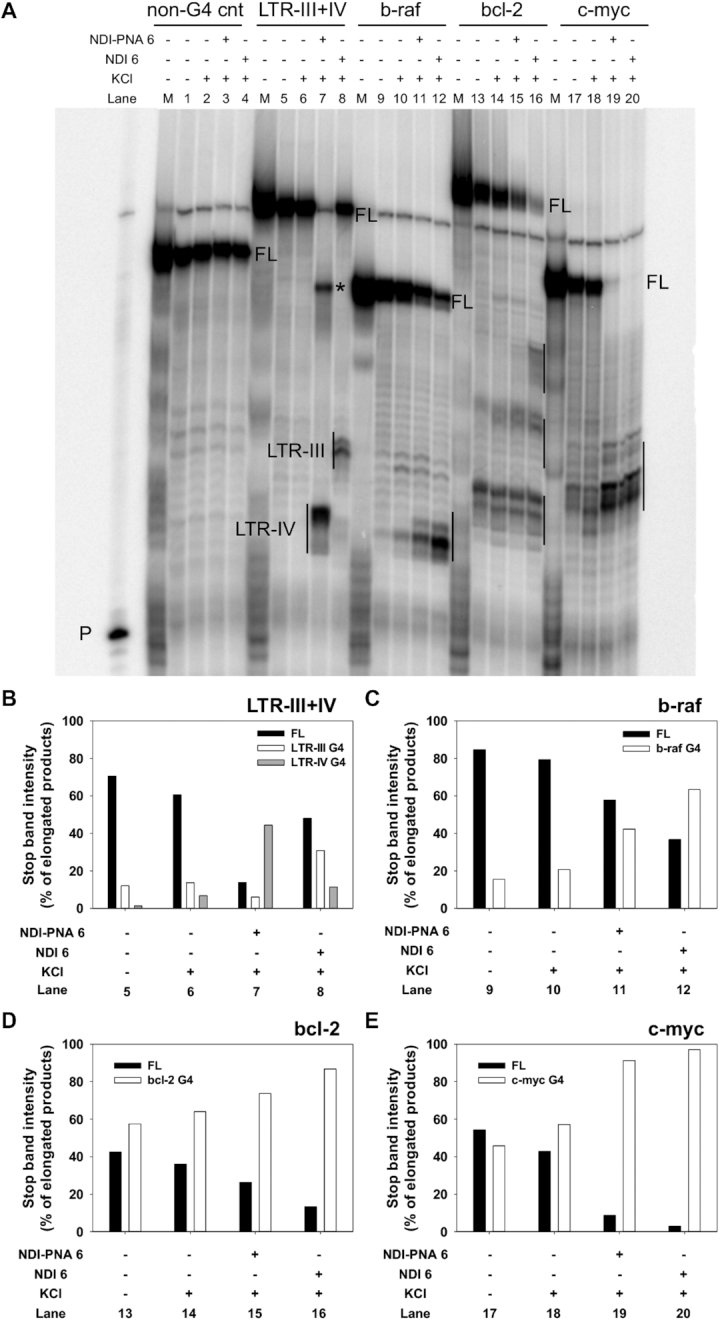
Taq polymerase stop assay on control G4-forming templates devoid of the PNA complementary sequence. (**A**) LTR-III+IV, b-raf, bcl-2 and c-myc templates were amplified by Taq polymerase at 60°C in the absence (lanes 5, 9, 13 and 17) and presence of 1 × 10^−1^ M KCl, alone (lanes 6, 10, 14 and 18) or with 4 × 10^−7^ M of **NDI-PNA 6** (lanes 7, 11, 15 and 19) or NDI **6** (lanes 8, 12, 16 and 20). A template (non-G4 cnt) made of a scrambled sequence unable to fold into G4 was also used as internal control (lanes 1–4). Lane P: unreacted labeled primer. Lane M: ladder of markers obtained by the Maxam and Gilbert sequencing carried out on the amplified strand complementary to the template strand. Vertical bars indicate G4-specific Taq polymerase stop sites. * indicates the stop site corresponding to the binding of the PNA moiety to its complementary sequence on the LTR-III+IV template. (**B**) Quantification of lanes 5–8 shown in panel A. Quantification of stop bands corresponding to LTR-III, LTR-IV G4s and of the full-length amplification product (FL) is shown. (**C**) Quantification of lanes 9–12 shown in panel A. Quantification of stop bands corresponding to b-raf G4 and of the full-length amplification product (FL) is shown. (**D**) Quantification of lanes 13–16 shown in panel A. Quantification of stop bands corresponding to bcl-2 G4 and of the full-length amplification product (FL) is shown. (**E**) Quantification of lanes 17–20 shown in panel A. Quantification of stop bands corresponding to c-myc G4 and of the full-length amplification product (FL) is shown.

A similar behavior was observed on other control G4-forming templates (i.e. b-raf, bcl-2, c-myc) devoid of the PNA complementary sequence (Figure 6). In all these control sequences, the stop sites were less intense and the full-length products more abundant when the template was treated with **NDI-PNA 6** compared to NDI **6** (Figure 6A, comparison between lanes 11 and 12, lanes 15 and 16, lanes 19 and 20). This suggests that when the DNA target sequence is absent, the PNA moiety partially hinders the binding of the NDI to the G4 (Figure 6B–E).

Finally, none of the compounds interfered with polymerase progression on the control non-G4 forming template (Figure 5A, lanes 3–7). In good agreement with CD experiments, these data indicate that the PNA moiety in the **NDI-PNA 6** conjugate allows to reach selectivity. In fact, it directed the NDI stabilizing activity toward the barely populated LTR-IV G4 compared to the naturally forming LTR-III G4.

**Figure 7.**
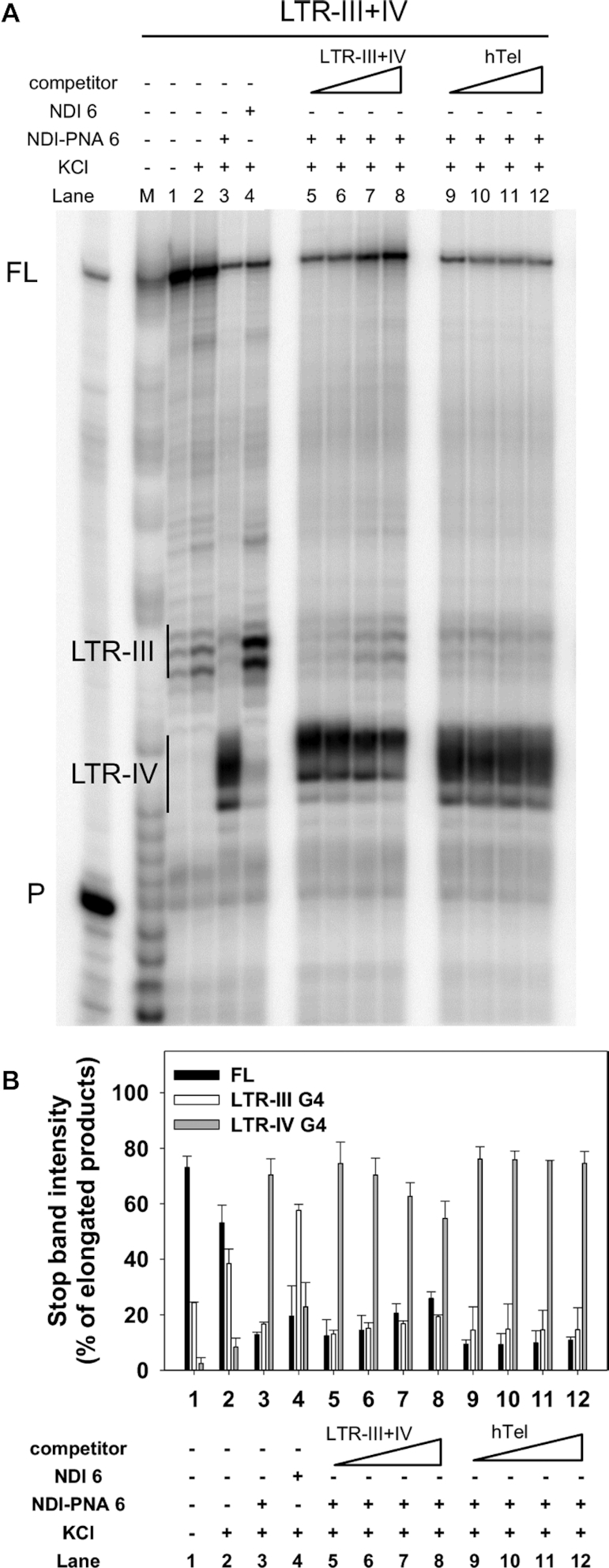
Competition Taq polymerase stop assay. (**A**) LTR-III+IV template was amplified by Taq polymerase at 42°C in the presence of 1 × 10^−1^ M KCl combined with a constant amount (4 × 10^−7^ M) of **NDI-PNA 6** and increasing concentrations (1–8-fold) of competitor, LTR-III+IV (lanes 5–8) or hTel (lanes 9–12). Lanes without competitor (lanes 1–4) were used as internal controls, in the absence (lane 1) and presence of 1 × 10^−1^ M KCl, alone (lane 2), or combined with 4 × 10^−7^ M of **NDI-PNA 6** (lane 3) or NDI 6 (lane 4). Lane P: unreacted labeled primer. Lane M: ladder of markers obtained by the Maxam and Gilbert sequencing carried out on the amplified strand complementary to the template strand. Vertical bars indicate G4-specific Taq polymerase stop sites. (**B**) Quantification of lanes 1–12 shown in panel A. Quantification of stop bands corresponding to LTR-III, LTR-IV G4s and of the full-length amplification product (FL) is shown.

The degree of specificity was next tested in a competition assay. We performed a Taq polymerase stop assay on the LTR-III+IV template in the presence of increasing amounts of unlabeled hTel or LTR-III+IV G4 competitors. These displayed similar *T*_m_ (64.9 ± 0.5°C and 68.7 ± 0.1°C, respectively) under these conditions, therefore allowing a meaningful comparison. The intensity of LTR-IV stop induced by the interaction of the template with **NDI-PNA 6** did not significantly decrease even in the presence of 8 molar equivalents of hTel competitor (Figure 7A, lanes 9–12, and Figure 7B). At the same time, the LTR-IV stop faded in a concentration-dependent manner upon self-competition with the unlabeled LTR-III+IV, while the stop corresponding to LTR-III and the full-length product were partially restored (Figure 7A, lanes 5–8, and Figure 7B). This confirms that **NDI-PNA 6** preferentially binds and stabilizes LTR-IV G4 over LTR-III and hTel G4s.

**Figure 8.**
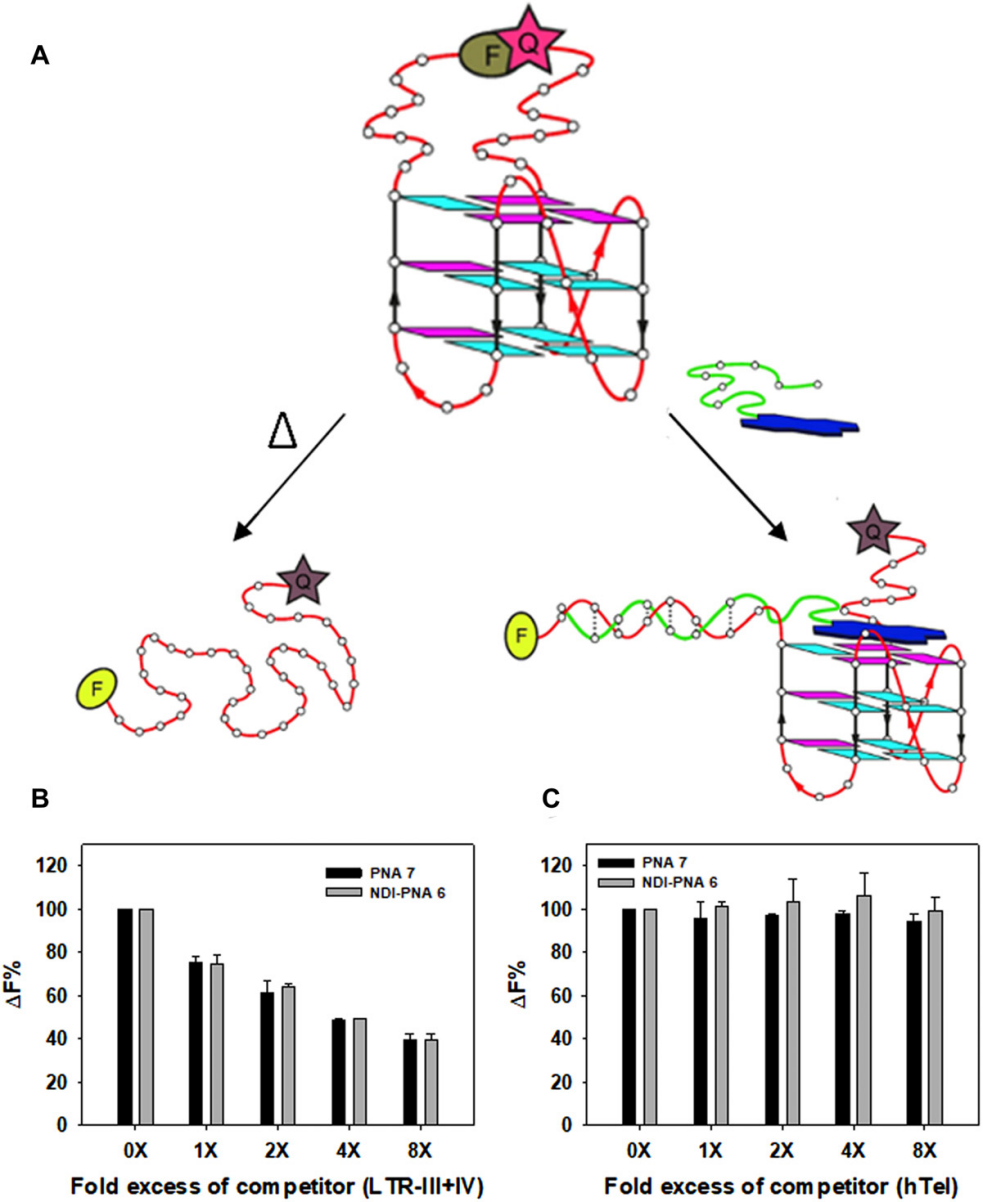
Isothermal FRET assay. (**A**) Schematics of the experiment: classic FRET melting assay is carried out monitoring the donor fluorescence (F) upon heating of the NA that disrupts the fluorescence donor (F)/quencher (Q) interaction (top). In our modified isothermal assay, the disruption of the F/Q interaction occurs at r.t., upon hybridization with the conjugate (bottom). (A, B) Isothermal FRET assay results obtained for 5′-FAM and 3′-TAMRA labeled LTR-III+IV (2.5 × 10^−7^ M) mixed with increasing concentrations (1–8×) of unlabeled competitor, (**B**) LTR-III+IV or (**C**) hTel, and a constant amount (1 × 10^−6^ M) of **NDI-PNA 6** or **PNA 7**. Δ*F*% is calculated as (Δ*F*_1_/Δ*F*_2_) × 100, where Δ*F*_1_ is the difference between the fluorescence of the labeled NA in the presence of both **NDI-PNA 6** or **PNA 7** and competitor, and the basal fluorescence of the NA alone, while Δ*F*_2_ is the difference in fluorescence measured without competitor.

To get a further and independent proof of specificity in competing conditions, we set up an isothermal FRET assay (Figure 8A), based on the adaptation of the classic FRET melting assay (63). In the classic test, recovery of the donor fluorescence is monitored upon heating, which unfolds the G4 and disrupts the G4–ligand complex. In contrast, here fluorescence was measured at r.t. with G4 unfolding prompted by hybridization of the PNA with the nucleic acid sequence proximal to the fluorophore. Higher or lower donor fluorescence at r.t. indicates the degree of interaction with the PNA in the conjugate and, upon competition with another substrate, the amount of ligand still bound to the labeled substrate. We thus recorded fluorescence of the 5′-FAM-/3′-TAMRA-labeled LTR-III+IV sequence in the presence of **NDI-PNA 6** or **PNA 7** (4 molar equivalents) adding increasing concentrations of the unlabeled LTR-III+IV or hTel competitors (1–8-fold excess). In good agreement with the Taq polymerase stop assay, the FAM fluorescence decreased in a dose-dependent manner in the presence of the unlabeled LTR-III+IV competitor (Figure 8B), while it was maintained in the presence of hTel (Figure 8C). This further confirms that both **NDI-PNA 6** and **PNA 7** preferentially bind LTR G4 with respect to hTel G4.

Disruption of the fluorescence pair by **NDI-PNA 6** was also confirmed in a control FRET melting assay (Supplementary Figure S10A). In the same assay, NDI **6** showed great stabilization of LTR-III+IV (Δ*T*_m_ = 21 ± 1°C) and no fluorescence perturbation (Supplementary Figure S10A), as proved by identical melting profiles of a dsDNA in the presence/absence of NDI **6** (Supplementary Figure S10B).

We next extended isothermal competition FRET assay to other G4 forming sequences found in oncogene promoters, such as bcl-2, c-myc and b-raf, hTERT G4, the latter characterized by a long duplex stem (64) and a dsDNA sequence. In all cases, the FAM fluorescence of the labeled LTR-III+IV was maintained in the presence of NDI-PNA 6 and the unlabeled competitors (Supplementary Figure S10C), indicating high selectivity of NDI-PNA 6 for its target G4 over other structures.

**Figure 9.**
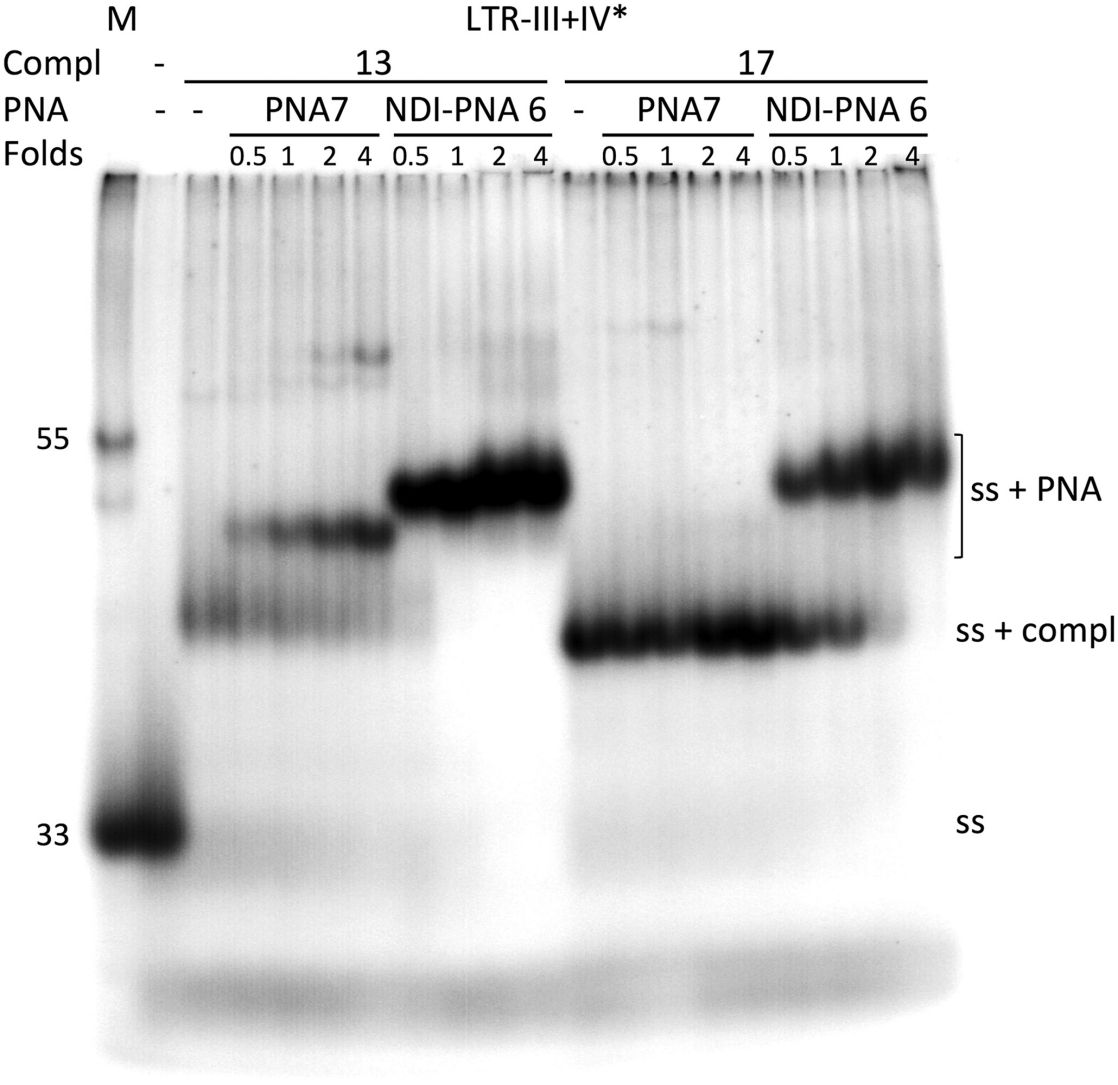
Strand displacement analysis by EMSA. Labeled LTR-III+IV (LTR-III+IV*) was annealed to 1.1-fold excess of LTR-III+IV complementary sequences (Compl) of different lengths (13 or 17 nucleotides) and incubated with increasing fold excess (0.5-4X) of **NDI-PNA 6** or **PNA 7** (PNA), as indicated, at 37°C, overnight. Reaction solutions were loaded onto 16% native polyacrylamide gel in 1X TBE buffer and KCl (1 × 10^−1^ M). The gel was run overnight at 60 V and DNA molecules were visualized by phosphorimaging. Lane M: markers of 33 and 55 nt-long oligonucleotides. ‘Ss’ indicates the folded LTR-III+IV* oligonucleotide; ‘ss + compl’ indicates the LTR-III+IV* oligonucleotide complemented to the 13- or 17-nt long oligonucleotides; ‘ss + PNA’ indicates the complex between the LTR-III+IV* oligonucleotide and the indicated PNA derivative.

We next evaluated the ability of **NDI-PNA 6** to induce strand displacement in a double-stranded context (Figure 9 and Supplementary Figure S11). To this end, we annealed the labeled LTR-III+IV to 1.1-fold excess of complementary sequences of different lengths (from 6 to 33 nucleotides). Only the duplex obtained with complementary sequences equal or longer that 13-nt was stable enough to be observed in these experimental conditions (Supplementary Figure S11). To assess the ability of the PNA moiety to displace the complementary strand, 16-fold excess of **NDI-PNA 6** or **PNA 7** were incubated with the double-stranded oligonucleotides (Supplementary Figure S11). **NDI-PNA 6** was able to fully displace the complementary strand when this was up to 17 nt-long and to partially displace the 26 and 21-nt long complementary strand (Supplementary Figure S11A). In contrast, **PNA 7** induced strand displacement of only the 13-nt long complementary strand (Supplementary Figure S11B). When **NDI-PNA 6** or **PNA 7** were added at lower fold excess (0.5-4) in the presence of 13- and 17-nt long complementary sequences (Figure 9), **NDI-PNA 6** was able to fully displace the 13-nt long oligonucleotide even at the lowest concentration (0.5-fold excess). The 17-nt long sequence was fully displaced at 2-fold excess, while >50% of displacement was observed at 0.5-fold excess. In contrast, in the case of **PNA 7**, full displacement was obtained only with the 13-nt long sequence, while no displacement was obtained with the 17-nt long sequence (Figure 9). These data indicate that the concurrent presence of the NDI and PNA highly improves the displacement activity of the PNA moiety.

### Cell entry and localization assessment

**Figure 10.**
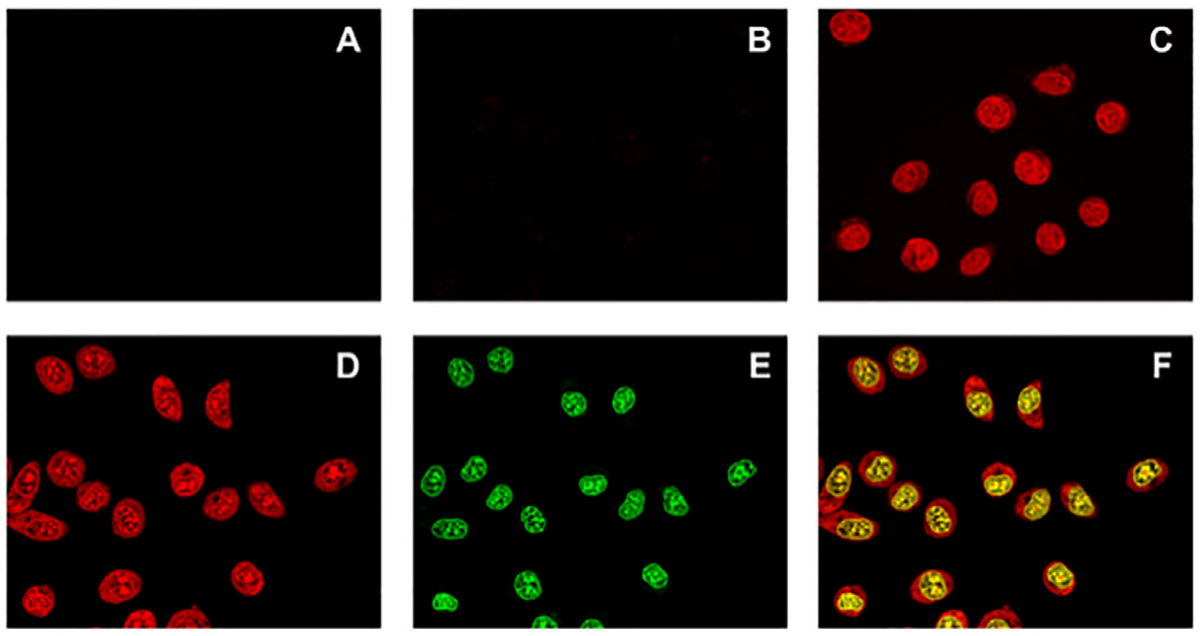
Evaluation of cell entry by confocal microscopy. Images of (**A**) untreated TZM-bl cells, TZM-Bl cells treated with 5 × 10^−5^ M of (**B**) **NDI-PNA 6**, (**C**) NDI **6** or (**D–F**) **NDI-PNA 8**. The –compounds were incubated with the cells for 2 h before cell fixation in 2% PFA. Nuclear staining was obtained with Nuclear Green LCS1. For NDI/conjugates (red channel) images (A-D) were visualized at 561 nm excitation wavelength and 570–620 nm emission range; for cell nuclei (green channel) (E) 488 nm excitation wavelength and 500–550 nm emission range were applied. (F) Overlap of panels (D) and (E).

Because of the reported poor PNA uptake by eukaryotic cells (65), we next evaluated the ability of our conjugates to enter into the cell, taking advantage of the intrinsic red emission of the NDI moiety (absorption and emission spectra are shown in Supplementary Figure S12). Experiments were carried out on TZM-Bl cells (Figure 10A), which were treated with **NDI-PNA 6** (Figure 10B) and control NDI **6** (Figure 10C). While NDI **6** readily entered the cell and localized into the nucleus (Figure 10C), the conjugate did not (as the emission was barely detectable, see Figure 10B). This clearly indicates that the PNA moiety hampers cell uptake. We reasoned that the PNA length could be a determining factor in the regulation of this effect. We thus synthesized **NDI-PNA 8** (Table 1), a shorter analogue of **NDI-PNA 6**, formed by a nine-monomer long PNA, since the 9-monomer **NDI-PNA 3** previously tested toward LTR-III had maintained good specificity for the target (Figure 4A, lane 19). Indeed, **NDI-PNA 8** activity and specificity were comparable to those of **NDI-PNA 6**, as assessed by Taq polymerase stop assay (Supplementary Figure S13). However, differently from **NDI-PNA 6, NDI-PNA 8** effectively entered the cell, similarly to NDI **6** (Figure 10D–F).

**Figure 11.**
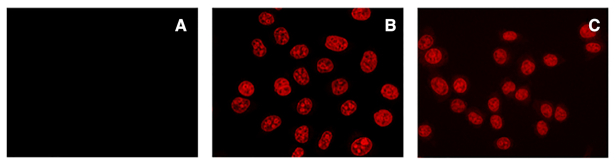
Evaluation of cell entry by confocal microscopy. Images of (**A**) untreated TZM-bl cells, TZM-Bl cells treated with 1 × 10^−5^ M of (**B**) **NDI-PNA 9** or (**C**) NDI **6**. The compounds were incubated with the cells for 1 h before cell fixation in 2% PFA. For NDI/conjugates images were visualized at 561 nm excitation wavelength and 570–620 nm emission range.

Based on these results, we inferred that G4 selectivity and effective cell entry are subjected to opposite PNA length requirements. We thus opted to maintain the PNA length that allowed the best G4 selectivity (and thus likely a lower number of off-target binding events in a complex system, such as the cellular context) and achieve cell entry by adding a nuclear localization sequence (NLS), already utilized to this end for bare PNA sequences (66). We thus conjugated **NDI-PNA 6** to the NLS PKKKRKV at its C-terminus, yielding **NDI-PNA 9** (Table 1). To avoid hampering conjugation to the first PNA monomer through the encumbered amine group of the proline residue, we inserted a glycine bridge between the peptide and the PNA sequence. Specificity of the recognition event was confirmed by means of Taq polymerase stop assay (Supplementary Figure S14) and by competition in EMSA: the first demonstrated that **NDI-PNA 9** stopped enzyme progression only in the presence of its specific target template (i.e. LTR-III+IV), while it not display any effect on the hTel template; the latter showed that formation of the complex between LTR-III+IV and **NDI-PNA 9** was efficiently competed by LTR-III+IV itself, while hTel did not compete to any extent (Supplementary Figure S15A). CD analysis further confirmed specificity: **NDI-PNA 9** largely modified the topology of LTR-III+IV, while not that of hTel (Supplementary Figure S15B). Cell entry was then verified by means of confocal microscopy: notably, the new conjugate efficiently permeated the cellular membrane and went into the nucleus (Figure 11B). All compounds were not cytotoxic at 3 h incubation at 10 μM as assessed by MTT assay (Supplementary Figure S16A). At higher concentrations (50 μM) only NDI **6** displayed cell viability reduction, while the **NDI-PNA** and **PNA** derivatives maintained their non-cytotoxic behavior. At 48 h incubation, NDI **6** displayed full cytotoxicity at 1 μM (Supplementary Figure S16B), while **NDI-PNA 8** and **NDI-PNA 9** became cytotoxic only at and >10 μM (Supplementary Figure S16C). These data indicate that addition of the PNA moiety to the NDI in general decreases compound cytotoxicity. The least cytotoxic compound was **NDI-PNA 6**, which decreased cell viability by only 30% at the highest tested concentration (Supplementary Figure S16B): this behavior is likely due to its limited ability to enter the cell (Figure 10B).

We next evaluated cell entry by confocal microscopy at higher and lower compound concentrations and longer incubation times. After 1 h of incubation with 1 × 10^−5^ M of **NDI-PNA 9**, fluorescence was detectable only in cell nuclei (Supplementary Figure S17 bottom left panel), proving the efficiency of the NLS peptide. No significant difference was found by increasing incubation times (Supplementary Figure S17), whereas, by decreasing compound concentration (up to 1 × 10^−6^ M) we observed a dramatic decrease of the signal (Supplementary Figure S18), fact that prevented testing for the specific targeting of the LTR region. At its detectable concentration (i.e. 5–10 × 10^−6^ M), **NDI-PNA 9** mainly colocalized with cellular DNA G4s, as proven by colocalization with an anti-G4 antibody (67) (Supplementary Figure S19) and DNase and RNase treatment (Supplementary Figure S20).

## DISCUSSION

In the last decades, reports of ligands capable of discriminating G4 structures over duplex DNA have flourished. However, targeting of a single G4 over many still remains elusive, although it is desirable for biological investigation, target validation, drug development and nanotechnology applications (68,69).

Few different approaches have been attempted in this direction. In 2009, Redman *et al.* demonstrated the feasibility of constructing high-affinity G4 selective ligands by appending peptide substituents to an acridine G4-binding scaffold. Molecular modeling suggested that the G4-interacting core stacked on the target tetrad and the peptide substituents made loop and groove contacts, thus allowing the discrimination between different G4 types (39). However, the choice of peptides was arbitrary and did not allow to tailor in advance the compounds for the G4 target of choice. Phan *et al.* described a dual-specific targeting strategy based on the simultaneous application of separated duplex- and G4-binding ligands, without structural conjugation. Combining the sequence specificity of duplex-binders and tight binding affinity of G4-binders, they provided the basis for the development of quadruplex-binding drugs specific to a single genomic G4 (40). Sugiyama *et al.* reported on hybrid molecules that were constructed with dual DNA-binding components, a cyclic imidazole/lysine polyamide (cIKP), and a hairpin pyrrole/imidazole polyamide (hPIP), directly bound with a linker. With this strategy, they aimed at specific quadruplex targeting by interaction with the duplex DNA sequence adjacent to the designated targets (41). Additionally, the use of a DNA sequence complementary to the target and connected to a G4-triggered fluorescent probe has been recently developed by Chen *et al.*, to uniquely recognize and visualize an RNA G4 (42). All approaches yielded valuable results. In this line of research, we here implemented the recognition of a G4 of choice developing original G4 ligands with unique recognition properties, red-fluorescent emission, resistance to degradation *in vivo* and easy adaptation to novel targets.

The newly developed NDI-PNA conjugates drive specificity by recognizing the flanking regions upstream or downstream of the target, according to natural base complementarity rules. Even if improvement in the ability to displace longer tract of dsDNA is to be sought, especially in the genome context, the NDI-PNAs are not subjected to fast degradation in the cellular environment. Even more importantly, the new compounds do not only stabilize naturally forming G4s, but also induce and stabilize G-rich sequences that have weak G4 forming tendency, while avoiding formation of neighboring stronger G4-folding sequences. This fact may have important therapeutic applications, for example pathological gene expression could be inhibited by G4 stabilization even when only weak G4 forming sequences are present at the relevant sites. On the opposite, pathologically relevant G4s may be released by favoring stabilization of adjacent non-critical G4s.

In addition, our strategy could also have important impact in nanotechnology applications. For example, G4s have been involved in long-range charge transports in molecular electronics (70) and as electron switches (71,72) and the possibility to control folding and unwinding of close and differently stable G4s could have a tremendous impact in this field.

To add to these highly favorable features, the NDI-PNA conjugates can be tailored against any G4 target of choice, using the universally known base complementarity rules in the design of the PNA moiety. Moreover, depending on the application, the cellular properties of the conjugates can be easily implemented by the insertion, for example, of a NLS peptide and taking advantage of the red emission associated to the NDI chromophore ligand, as shown here for the **NDI-PNA 9** compound. The conjugation of a PNA moiety can be conceived also for other G4 ligand cores: in fact, several of them have shown a certain degree of selectivity toward specific G4s (73–75) and the addition of PNA appendages would likely improve their performance by enhancing selectivity and proper localization. In this case the only limitation is the availability on the G4 ligand core of sites for PNA conjugation that do not interfere with the G4 binding activity.

To summarize, the new approach reported here presents several advantages: (i) it provides in principle specificity for any G4 of interest, through the straightforward engineering of the PNA sequence; (ii) it is independent of the G4 topology; (iii) it can potentially be adapted to both DNA and RNA targets, thanks to the PNA ability to hybridize with both NA duplexes (as in genomic DNA) and single strands (as in RNA targets); (iv) it does not rely on transfection steps, thanks to the possible insertion of localization peptide sequences; (v) being an intrinsically fluorescent ligand, it is a ready to use molecular device for cell imaging applications.

In conclusion, the present approach offers a versatile, selective and multifunctional molecular tool for wider applications, prompting the development of G4 binders specific for G4 involved in relevant human diseases and allowing new developments in NA-based nanotechnologies.

## Supplementary Material

gkaa186_Supplemental_File
