# Supplementary material for: Selective targeting of mutually exclusive DNA G-quadruplexes: HIV-1 LTR as paradigmatic model
Source: Nucleic Acids Res. 2020 Apr 13;48(9):4627–42. doi: 10.1093/nar/gkaa186 (PMC7229848; doi:10.1093/nar/gkaa186)
Supplement: gkaa186_Supplemental_File [file gkaa186_supplemental_file.pdf]

Supporting Information

Selective targeting of mutually exclusive DNA G-quadruplexes: HIV-1 LTR as paradigmatic model

Martina Tassinari[1†], Michela Zuffo[2†], Matteo Nadai[1], Valentina Pirota[2], Adriana Carolina Sevilla Montalvo[2], Filippo Doria[2], Mauro Freccero[2]*, Sara N. Richter[1]*

[1] Department of Molecular Medicine, University of Padova, via A. Gabelli 63, 35121 Padova, Italy.
[2] Department of Chemistry, University of Pavia, v. le Taramelli 10, 27100, Pavia, Italy.

Contents:

Scheme S1. p. S2
Table S1. Oligonucleotides used in this study p. S3
Figure S1. Representative CD spectra of LTR-II+III+IV, (B) LTR-II+III+IV+3' and hTel G4, alone or in the presence of **NDI-PNA 1**, **NDI-PNA 2** and NDI **6**. p. S4
Figure S2. CD thermal unfolding analysis of LTR-II+III+IV G4 p. S5
Figure S3. CD thermal unfolding analysis of hTel G4 p. S6
Figure S4. CD of LTR-III+IV and hTel G4 alone or in the presence of **NDI-PNA 6**, **PNA 7**, and NDI **6**. p. S7
Figure S5. Investigation of **NDI-PNA 6**-induced CD spectrum changes at the longest wavelengths p. S8
Figure S6. CD thermal unfolding of LTR-III+IV G4 alone or in the presence of **NDI-PNA 6**, NDI **6**, **PNA 7**. p. S9
Figure S7. CD thermal unfolding of hTel G4, alone or in the presence of **NDI-PNA 6**, NDI **6**, **PNA 7**. p. S11
Figure S8. CD spectra of LTR-III+IV in the presence of different amounts of **NDI-PNA 6** p. S13
Figure S9. CD thermal unfolding of LTR-III+IV, alone or in the presence of **NDI-PNA 6**. p. S14
Figure S10. FRET analysis. p. S16
Figure S11. Strand displacement analysis by EMSA. p. S17
Figure S12. Absorption and emission spectra of NDI **6** p. S18
Figure S13. Taq polymerase stop assay. LTR-III+IV and hTel templates in the absence and presence of **NDI-PNA 8**, **NDI-PNA 6** or NDI **6**. p. S19
Figure S14. Taq polymerase stop assay. LTR-III+IV and hTel templates in the absence and presence of **NDI-PNA 9, NDI-PNA 6**, **PNA 7** or NDI **6**. p. S21
Figure S15. EMSA competition analysis of the complex between LTR-III+IV and **NDI-PNA 9** with LTR-III+IV or hTel oligonucleotides. p. S23
Figure S16. TZM-bl cells viability. p. S24
Figure S17. Images of TZM-bl cells treated with $1x10^{-5}$ M of **NDI-PNA 9** at different incubation times (1, 4, 5 h). p. S25
Figure S18. Images of TZM-bl cells treated with different concentrations of **NDI-PNA 9 (**$1-50x10^{-6}$ M) for 1 h. p. S26
Figure S19. Colocalization of **NDI-PNA 9** with the anti-G4 antibody 1H6. p. S27
Figure S20. Images of untreated TZM-bl cells, treated with **NDI-PNA 9** alone or in the presence of DNase I or RNaseA. p. S28
HPLC purity data p. S29
NMR characterization p. S31
ESI-MS characterization of the NDI-PNAs p. S39

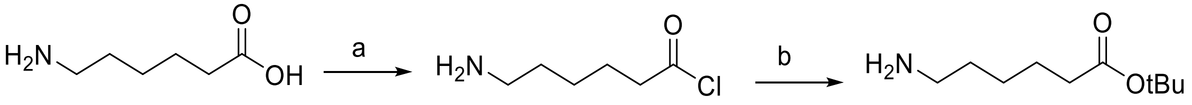

Scheme S1. a) $SOCl_2$, r.t., 1.5 h; b) tBuOH, $NaHCO_3$, r.t., 2 h

Table S1. Oligonucleotides used in this study

Application	Name	Sequence (5'→ 3')
CD	LTR-II+III+IV	GGGGACTTTCCAGGGAGGCGTGGCCTGGGCGGGACTGGGGAGT GG
	LTR-II+III+IV 3'	TGGGGACTTTCCAGGGAGGCGTGGCCTGGGCGGGACTGGGGAG TGGCGAGCCCT
	LTR-III+IV	GGGAGGCGTGGCCTGGGCGGGACTGGGGAGTGG
	hTel	AGGGTTAGGGTTAGGGTTAGGG
Taq polymerase stop assay	Primer	GGCAAAAAGCAGCTGCTTATATGCAG
	non-G4 cnt	TTGTCGTTAAAGTCTGACTGCGAGCTCTCAGATCCTGCATATAAG CAGCTGCTTTTTGCC
	LTR-II+III+IV	TTTTTGGGGACTTTCCAGGGAGGCGTGGCCTGGGCGGGACTGG GGAGTGG TTTTTCTGCATATAAGCAGCTGCTTTTTGCC
	LTR-III+IV	TTTTTGGGAGGCGTGGCCTGGGCGGGACTGGGGAGTGGTTTTTC TGCATATAAGCAGCTGCTTTTTGCC
	hTel	TTTTTGGGTTAGGGTTAGGGTTAGGGTTTTTCTGCATATAAGCGCT TTTTGCC
	LTR-III+IV competitor	GGGAGGCGTGGCCTGGGCGGGACTGGGGAGTGG
	hTel competitor	AGGGTTAGGGTTAGGGTTAGGG
FRET assay	LTR-III+IV labeled	FAM[1]- GGGAGGCGTGGCCTGGGCGGGACTGGGGAGTGG-TAMRA[2]
	dsDNA labeled	FAM[1] -CTATAGCGCGCTATAG -TAMRA[2]
	LTR-III+IV unlabeled	GGGAGGCGTGGCCTGGGCGGGACTGGGGAGTGG
	hTel unlabeled	AGGGTTAGGGTTAGGGTTAGGG
	c-myc unlabeld	TGGGGAGGGTGGGGAGGGTGGGGAAGG
	bcl-2 unlabeled	AGGGGCGGGCGCGGGAGGAAGGGGGCGGGAGCGGGGCTG
	b-raf unlabeled	GGGCGGGGAGGGGGAAGGGA
	dsDNA unlabeled	CAATCGGATCGAATTCGATCCGATTG
	hTERT unlabeled	AGGGGAGGGGCTGGGAGGGCCCGGAGGGGGCTGGGCCGGGGA CCCGGGAGGGGTCGGGACGGGGCGGGGT
EMSA	LTR-III+IV	GGGGACTTTCCAGGGAGGCGTGGCCTGGGCGGGACTGGGGAGT GG
	LTR-III+IV C 33	CCACTCCCCAGTCCCGCCCAGGCCACGCCTCCC
	LTR-III+IV C 30	CTCCCCAGTCCCGCCCAGGCCACGCCTCCC
	LTR-III+IV C 26	CCAGTCCCGCCCAGGCCACGCCTCCC
	LTR-III+IV C 21	CCCGCCCAGGCCACGCCTCCC
	LTR-III+IV C 17	CCCAGGCCACGCCTCCC
	LTR-III+IV C 13	GGCCACGCCTCCC
	LTR-III+IV C 9	GGCCACGCC
	LTR-III+IV C 6	GGCCAC
	hTel	AGGGTTAGGGTTAGGGTTAGGG

[1] 6-carboxyfluorescein. [2] 6-carboxy-tetramethylrhodamine.

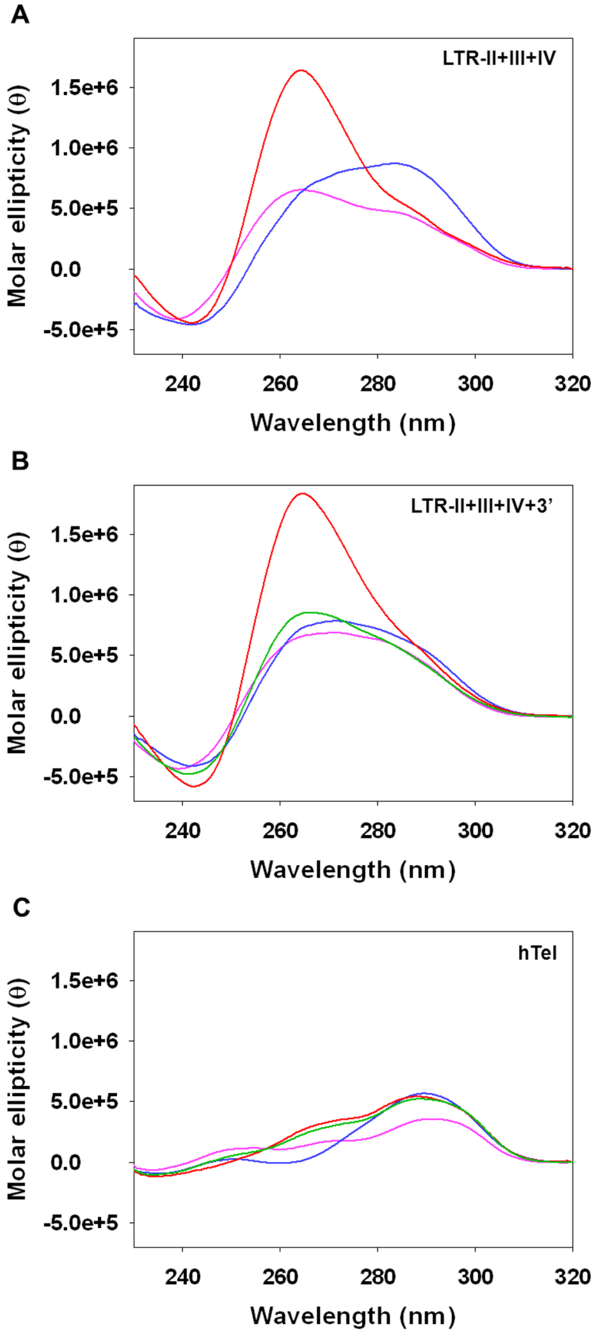

Figure S1. Representative CD spectra of (**A**) LTR-II+III+IV, (**B**) LTR-II+III+IV+3' and (**C**) hTel G4 ($2x10^{-6}$ M) at $1x10^{-1}$ M KCl, alone (magenta) or in the presence of 4-fold excess of **NDI-PNA 1** (red), **NDI-PNA 2** (green), NDI **6** (blue).

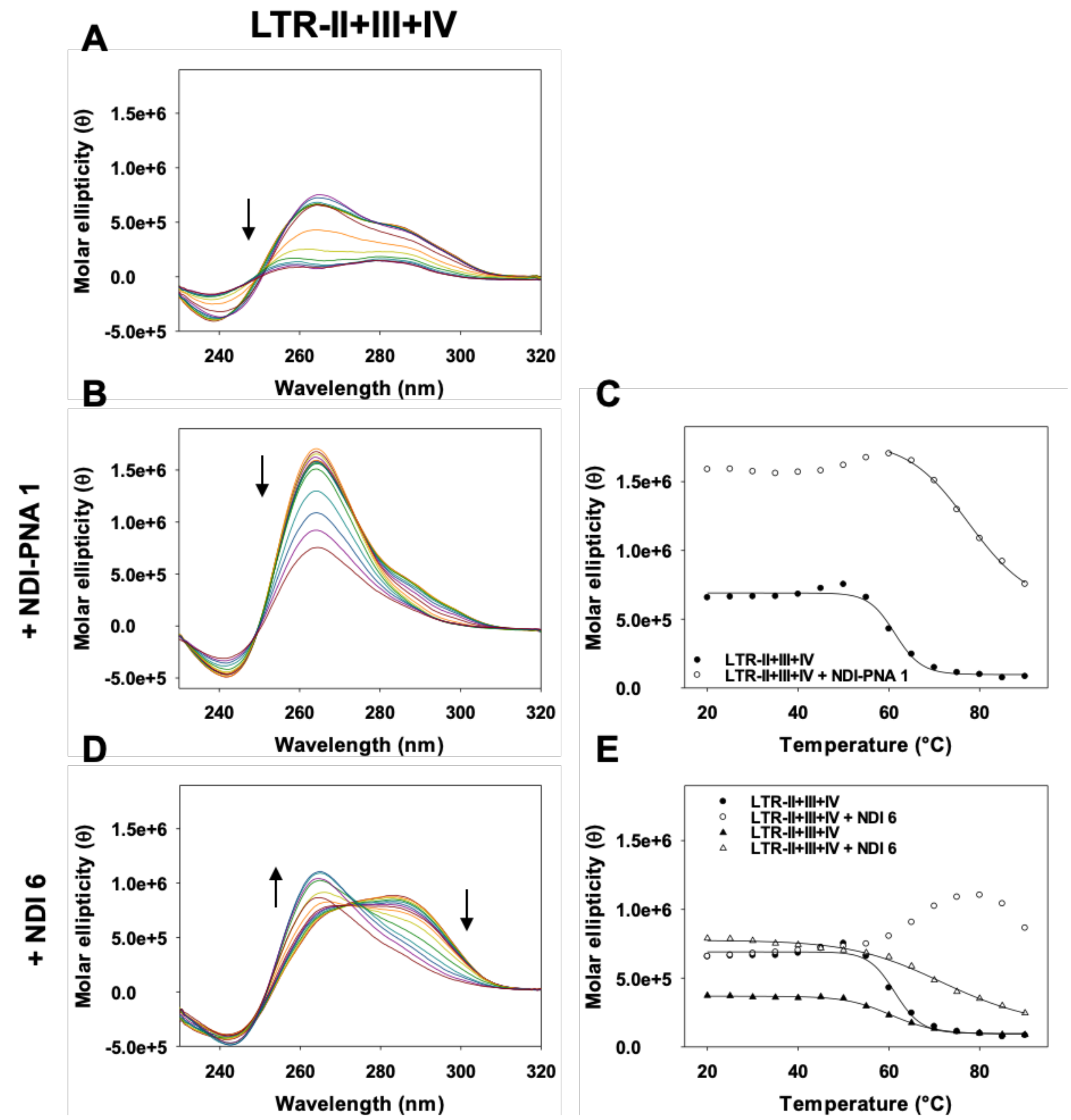

Figure S2. CD thermal unfolding analysis of LTR-II+III+IV G4 ($2x10^{-6}$ M) at $1x10^{-1}$ M KCl, alone (**A**) or in the presence of 4-fold excess of (**B**) **NDI-PNA 1** or (**D**) NDI **6**. Panels **A**, **B**, **D**, show CD spectra variation from 20 °C to 90 °C as a function of the wavelength; arrows indicate the spectral change from low to high temperatures. Panels **C** and **E** show the molar ellipticity at the peak wavelengths as a function of the temperature, fitted with the vant' Hoff equation, where possible. Circles and triangles indicate molar ellipticity values at 265 nm and 290 nm, respectively, in the absence (black) and presence (white) of the indicated ligand.

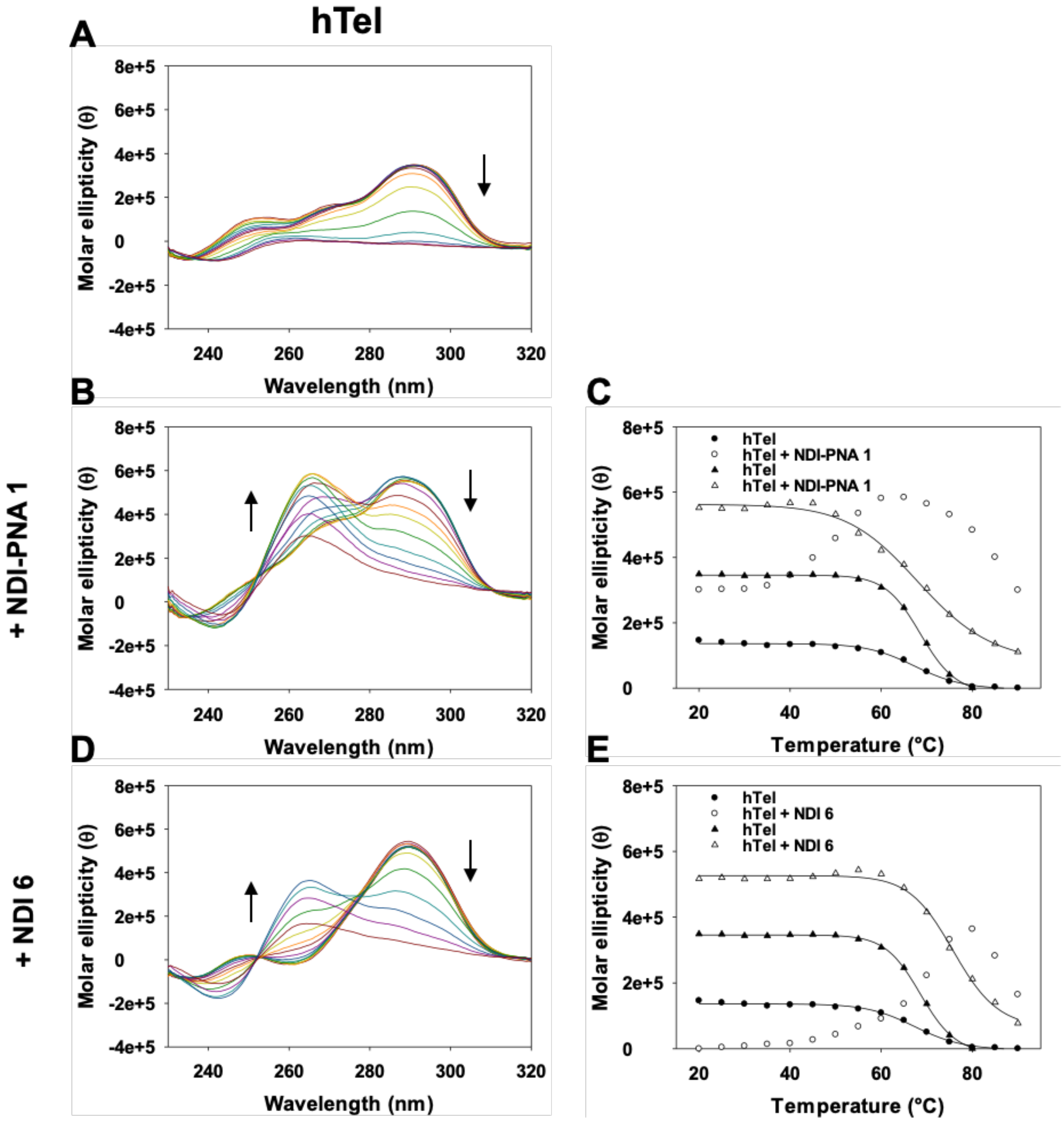

Figure S3. CD thermal unfolding analysis of hTel G4 ($2x10^{-6}$ M) at $1x10^{-1}$ M KCl, alone (**A**) or in the presence of 4-fold excess of (**B**) **NDI-PNA 1** or (**D**) NDI **6**. Panels **A**, **B**, **D**, show CD spectra variation from 20 °C to 90 °C as a function of the wavelength; arrows indicate the spectral change from low to high temperatures. Panels **C** and **E** show the molar ellipticity at the peak wavelengths as a function of the temperature, fitted with the vant' Hoff equation, where possible. Circles and triangles indicate molar ellipticity values at 265 nm and 290 nm, respectively, in the absence (black) and presence (white) of the indicated ligand.

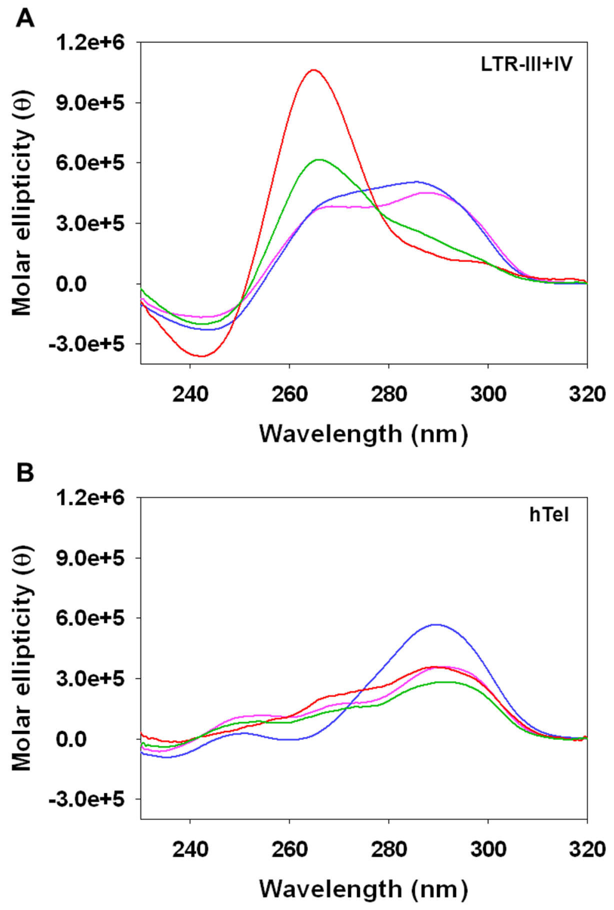

Figure S4. Representative CD spectra of (**A**) LTR-III+IV and (**B**) hTel G4 ($2x10^{-6}$ M) at $1x10^{-1}$ M KCl, alone (magenta) or in the presence of 4-fold excess of **NDI-PNA 6** (red), **PNA 7** (green), NDI **6** (blue).

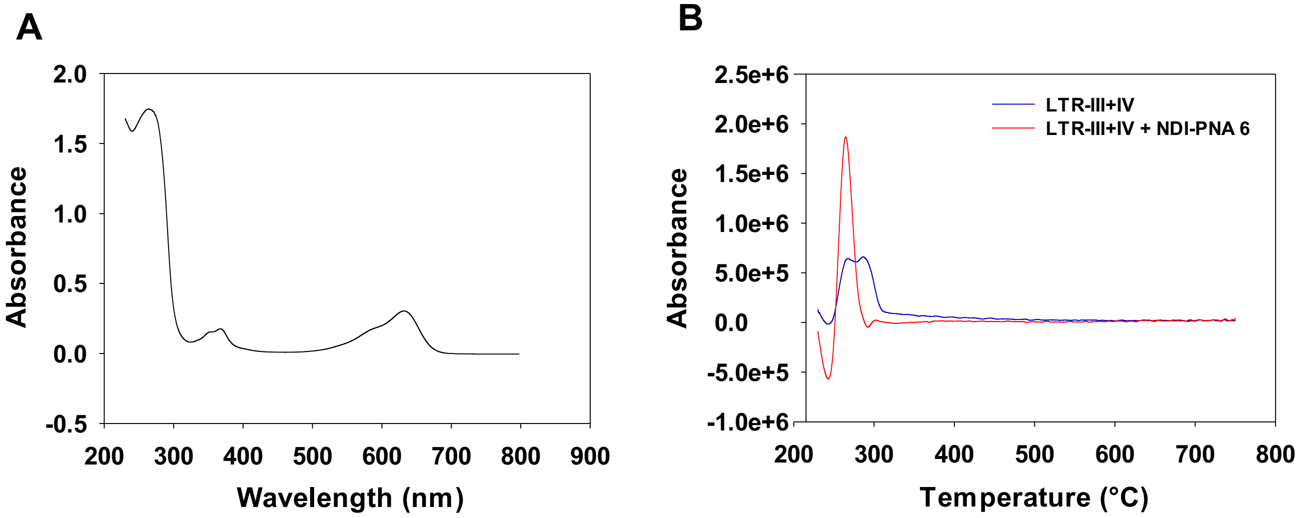

Figure S5. Investigation of **NDI-PNA 6**-induced CD spectrum changes at the longest wavelengths. (**A**) Absorption spectrum of **NDI-PNA 6** ($5x10^{-5}$ M) in lithium cacodylate buffer ($1x10^{-2}$ M, pH 7.4) and KCl ($1x10^{-1}$ M), measured between 230 and 800 nm. (**B**) CD spectra of LTR-III+IV ($2x10^{-6}$ M) in lithium cacodylate buffer ($1x10^{-2}$ M, pH 7.4) and KCl ($1x10^{-1}$ M), in the presence or absence of 4-fold excess of **NDI-PNA 6**, recorded over a wavelength range of 230-750 nM.

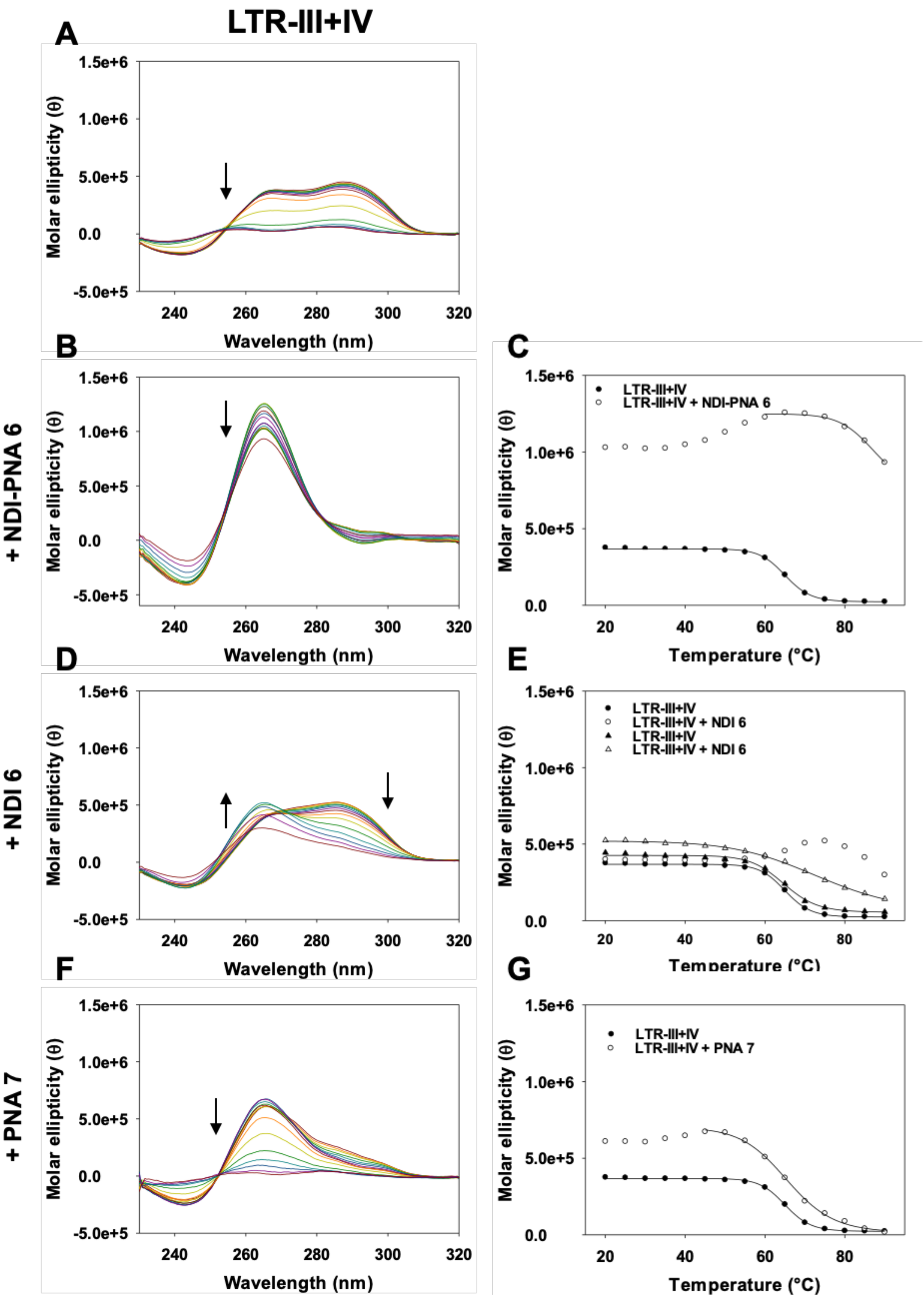

LTR-III+IV
A
B
C
D
E
F
G
+ NDI-PNA 6
+ NDI 6
+ PNA 7
Molar ellipticity (θ)
Wavelength (nm)
Temperature (°C)
LTR-III+IV
LTR-III+IV + NDI-PNA 6
LTR-III+IV + NDI 6
LTR-III+IV + PNA 7

Figure S6. CD thermal unfolding analysis of LTR-III+IV G4 ($2x10^{-6}$ M) at $1x10^{-1}$ M KCl, alone (**A**) or in the presence of 4-fold excess of (**B**) **NDI-PNA 6**, (**D**) NDI **6**, (**F**) **PNA 7**. Panels **A**, **B**, **D**, **F** show CD spectra variation from 20 °C to 90 °C as a function of the wavelength; arrows indicate the spectral change from low to high temperatures. Panels **C**, **E**, **G** show the molar ellipticity at the peak wavelengths as a function of the temperature, fitted with the vant' Hoff equation, where possible. Circles and triangles indicate molar ellipticity values at 265 nm and 290 nm, respectively, in the absence (black) and presence (white) of the indicated ligand.

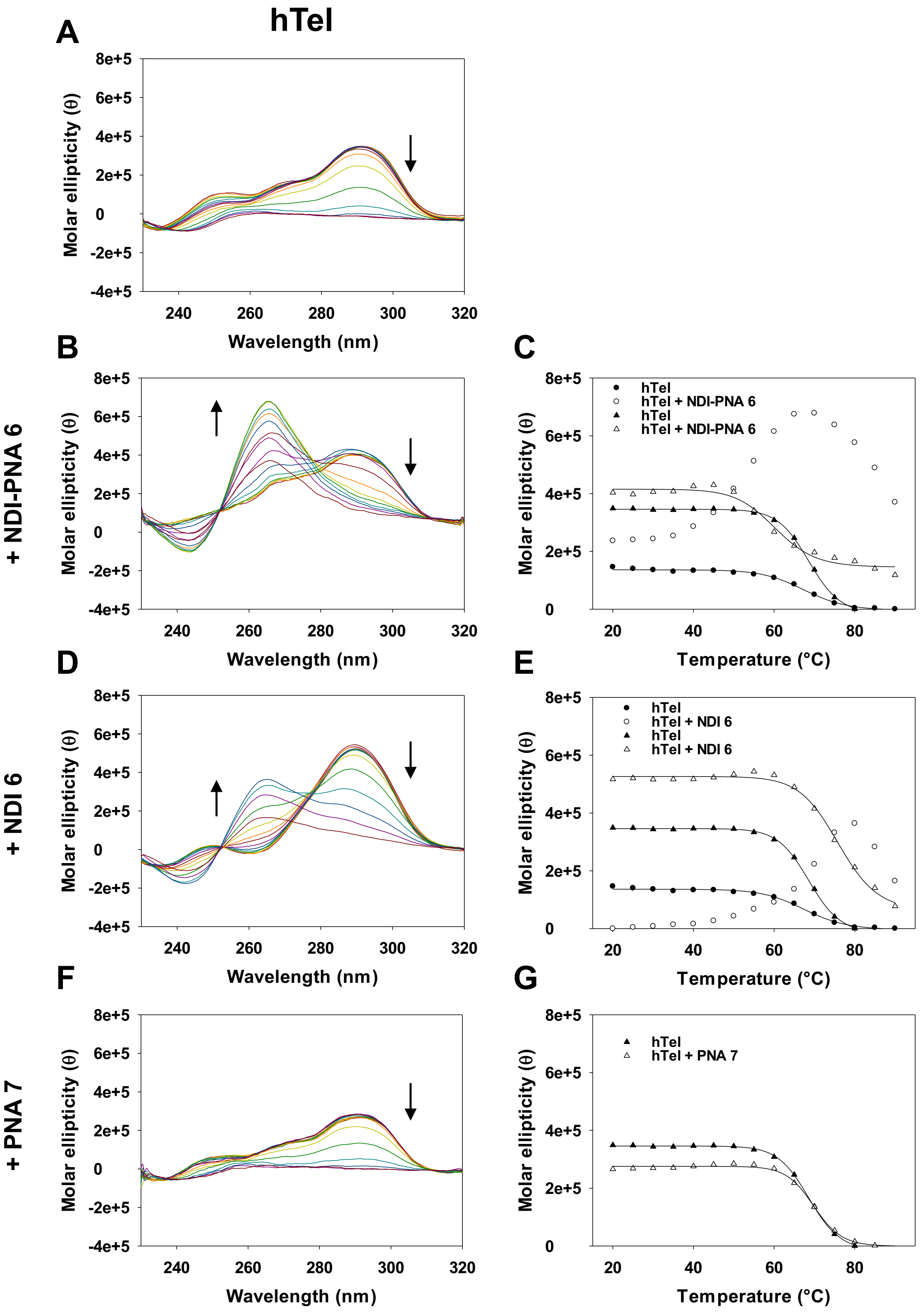
hTel
A
B
C
D
E
F
G
+ NDI-PNA 6
+ NDI 6
+ PNA 7
Molar ellipticity (θ)
Wavelength (nm)
Temperature (°C)
8e+5
6e+5
4e+5
2e+5
0
-2e+5
-4e+5
240
260
280
300
320
20
40
60
80
hTel
hTel + NDI-PNA 6
hTel
hTel + NDI-PNA 6
hTel
hTel + NDI 6
hTel
hTel + NDI 6
hTel
hTel + PNA 7

Figure S7. CD thermal unfolding analysis of hTel G4 ($2x10^{-6}$ M) at $1x10^{-1}$ M KCl, alone (**A**) or in the presence of 4-fold excess of (**B**) **NDI-PNA 6**, (**D**) NDI **6**, (F) **PNA 7**. Panels **A**, **B**, **D**, **F** show CD spectra variation from 20 °C to 90 °C as a function of the wavelength; arrows indicate the spectral change from low to high temperatures. Panels **C**, **E**, **G** show the molar ellipticity at the peak wavelengths as a function of the temperature, fitted with the vant' Hoff equation, where possible. Circles and triangles indicate molar ellipticity values at 265 nm and 290 nm, respectively, in the absence (black) and presence (white) of the indicated ligand.

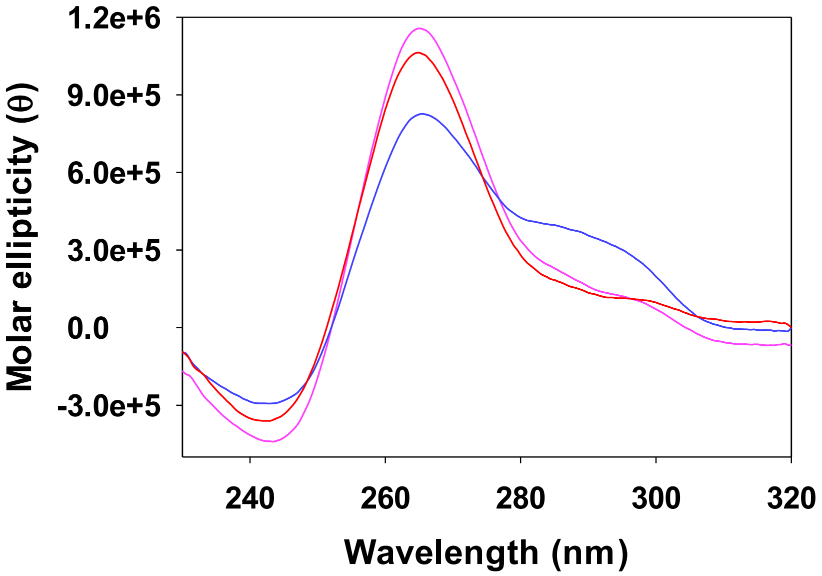

Figure S8. Representative CD spectra of LTR-III+IV at $1x10^{-1}$ M KCl in the presence of different amounts of **NDI-PNA 6**: 4-fold excess (red), 2-fold excess (magenta), 1-fold excess (blue).

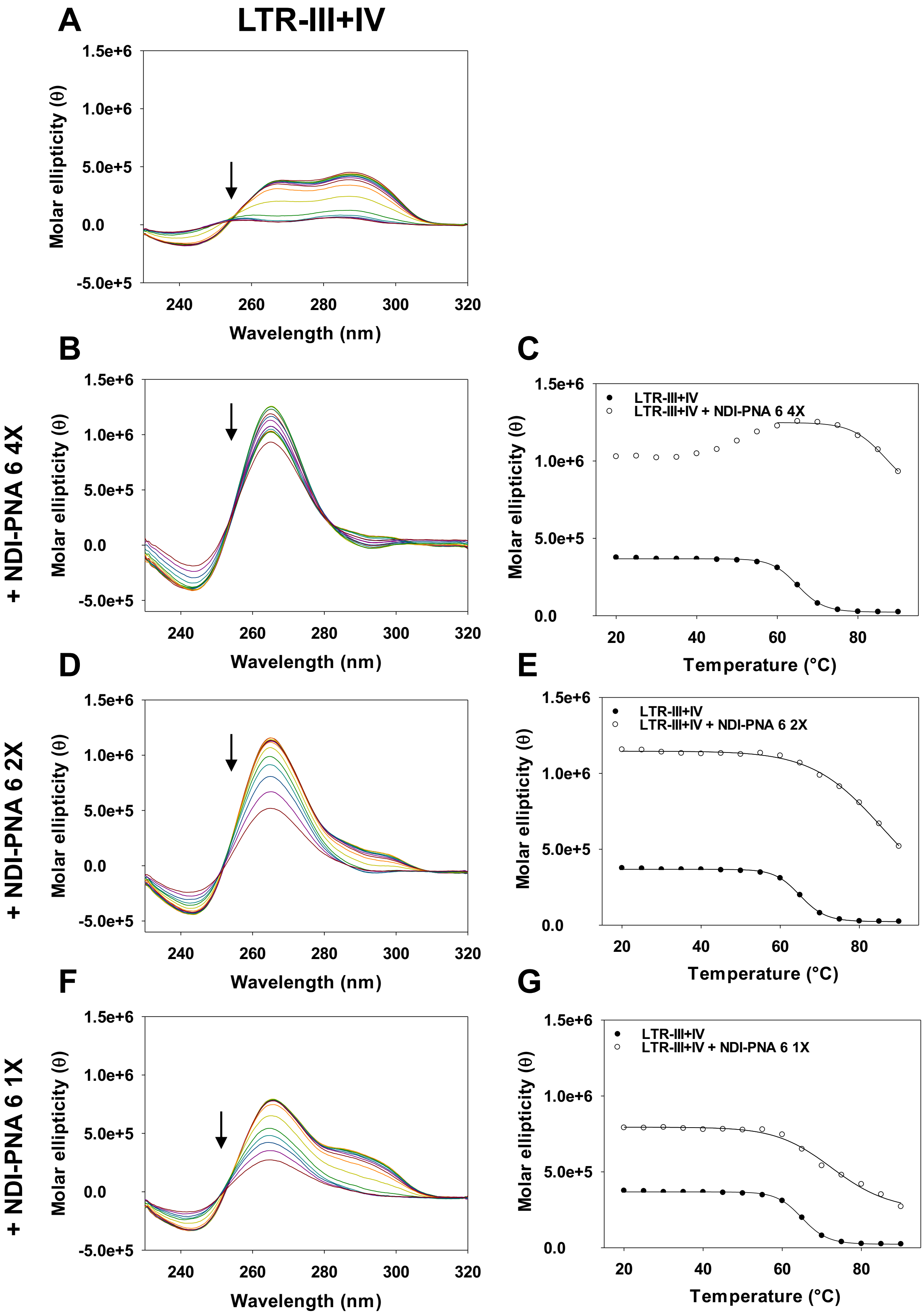
A
LTR-III+IV
Molar ellipticity (θ)
1.5e+6
1.0e+6
5.0e+5
0.0
-5.0e+5
240
260
280
300
320
Wavelength (nm)
B
+ NDI-PNA 6 4X
C
LTR-III+IV
LTR-III+IV + NDI-PNA 6 4X
20
40
60
80
Temperature (°C)
D
+ NDI-PNA 6 2X
E
LTR-III+IV
LTR-III+IV + NDI-PNA 6 2X
F
+ NDI-PNA 6 1X
G
LTR-III+IV
LTR-III+IV + NDI-PNA 6 1X

Figure S9. CD thermal unfolding analysis of LTR-III+IV G4 ($2x10^{-6}$ M) at $1x10^{-1}$ M KCl, alone (**A**) or in the presence of different amounts of **NDI-PNA 6**: (**B**) 4-fold excess, (**D**) 2-fold excess and (**F**) 1-fold excess. Panels **A**, **B**, **D**, **F** show CD spectra variation from 20 °C to 90 °C as a function of the wavelength; arrows indicate the spectral change from low to high temperatures. Panels **C**, **E**, **G** show the molar ellipticity at the peak wavelengths as a function of the temperature, fitted with the vant' Hoff equation, where possible. Circles and triangles indicate molar ellipticity values at 265 nm and 290 nm, respectively, in the absence (black) and presence (white) of the indicated ligand.

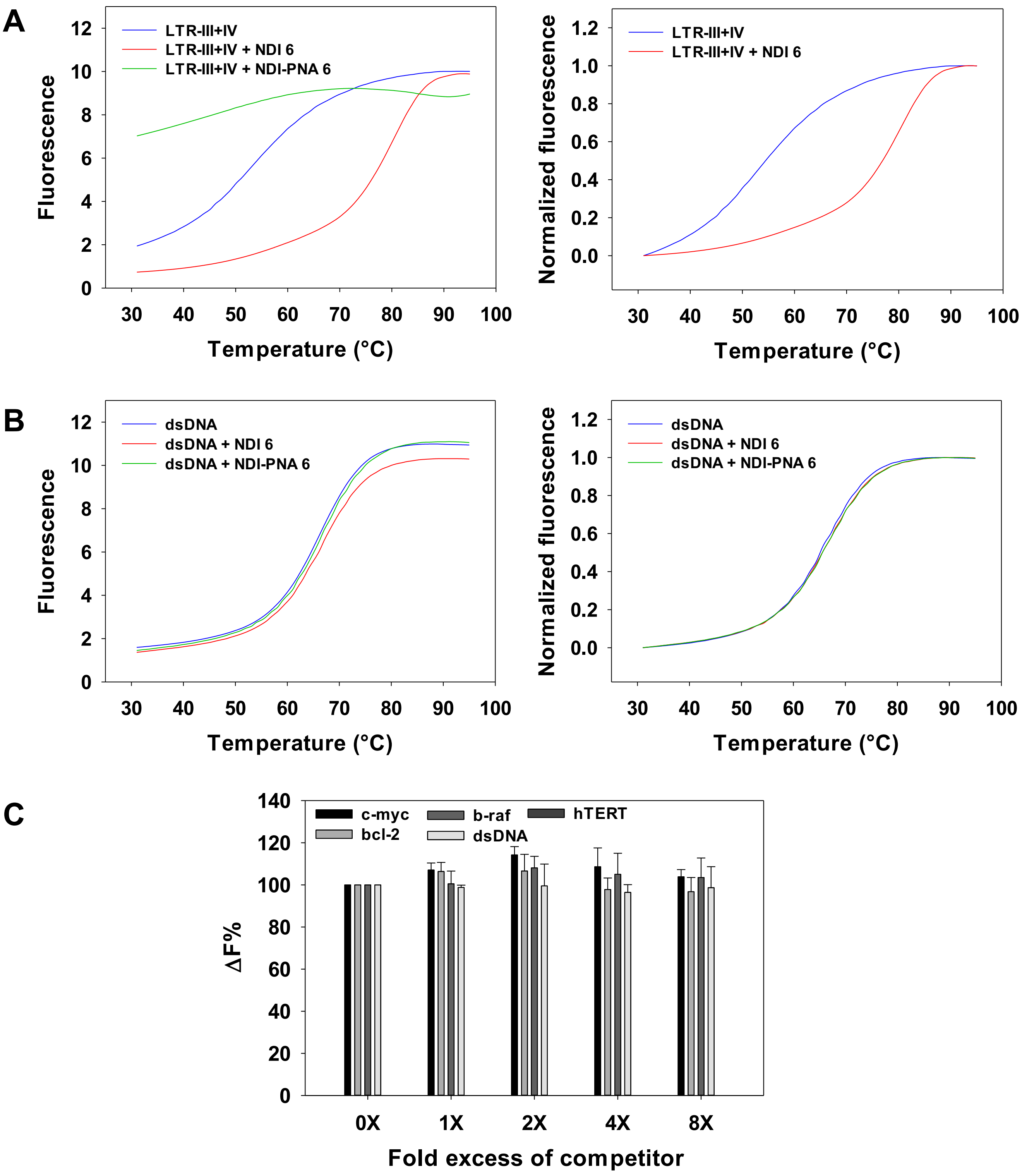

Figure S10. FRET analysis. (**A**) FRET-melting curves of the fluorescence end-labeled LTR-III+IV ($2.5x10^{-7}$ M), alone (blue curve) or in the presence $1x10^{-6}$ M of NDI **6** (red curve) or **NDI-PNA 6** (green curve). (**B**) FRET-melting curves of the fluorescence end-labeled dsDNA ($2.5x10^{-7}$ M)alone (blue curve) or in the presence of $1x10^{-6}$ M of NDI **6** (red curve) or **NDI-PNA 6** (green curve. (**C**) Isothermal FRET assay data obtained with the 5'-FAM and 3'-TAMRA unlabeled LTR-III+IV (2.5x10^{-}

[7] M) mixed with increasing concentrations (1-8X) of unlabelled competitors and a constant amount ($1x10^{-6}$ M) of **NDI-PNA 6.** ΔF% is calculated as $(\Delta F_1/\Delta F_2)x100$, where ΔF_1 is the difference between the fluorescence of the labeled NA in the presence of **NDI-PNA 6** and competitor, and the basal fluorescence of the NA alone, while ΔF_2 is the difference in fluorescence measured without competitor. The unlabeled LTR-III+IV competitor was tested in parallel, as internal positive control.

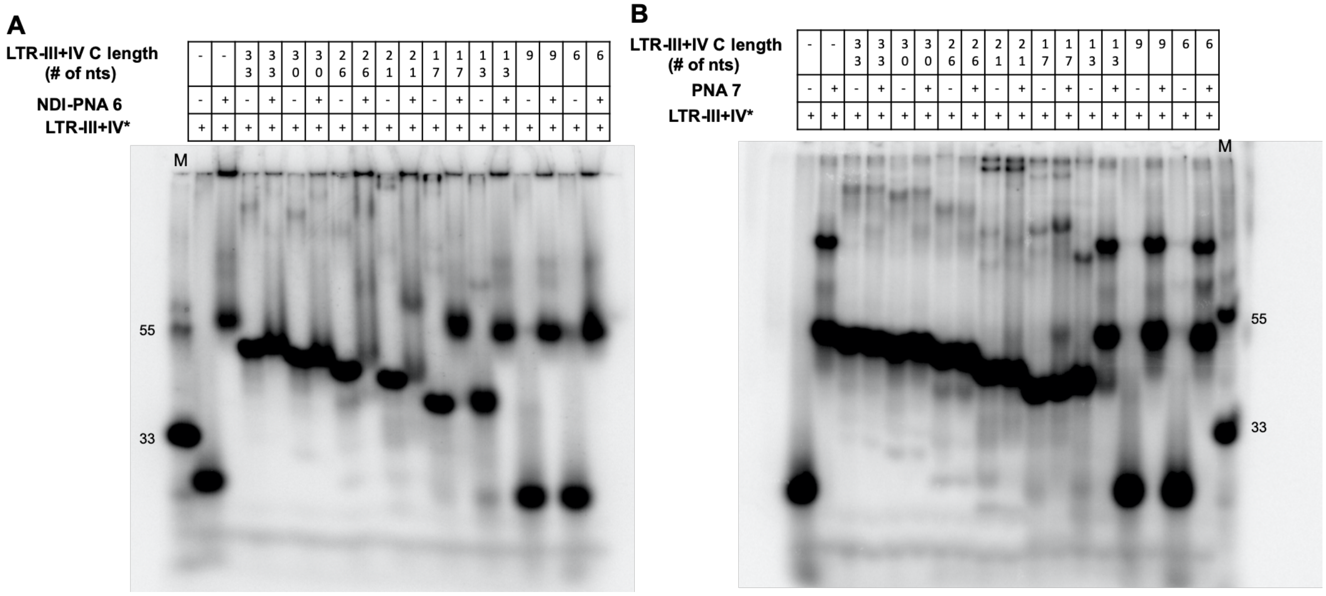

Figure S11. Strand displacement analysis by EMSA**.** Labeled LTR-III+IV was annealed to 1.1-fold excess of LTR-III+IV C complementary sequences of different lengths (from 6 to 33 nucleotides) and incubated with 16-fold excess of (**A**) **NDI-PNA 6** or (**B**) **PNA 7** at 37 °C, overnight. Reaction solutions were loaded onto 16% native polyacrylamide gel in 1X TBE buffer and KCl ($1x10^{-1}$ M). The gel was run overnight at 60 V and DNA molecules were visualized by phosphorimaging. Lane M: markers of 33 and 55-nt long oligonucleotides.

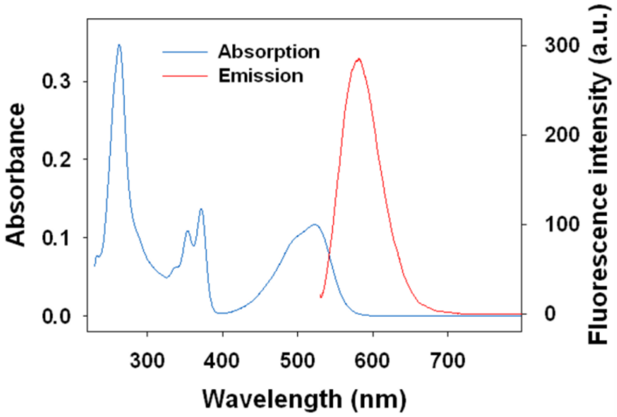

Figure S12. Absorption and emission (λ_{ex} = 523 nm) spectra of NDI **6** (1x10^{-5} M) in lithium cacodylate buffer (1x10^{-2} M, pH 7.4) and KCl (1x10^{-1} M), measured between 230 and 800 nm (λ_{em} = 580 nm).

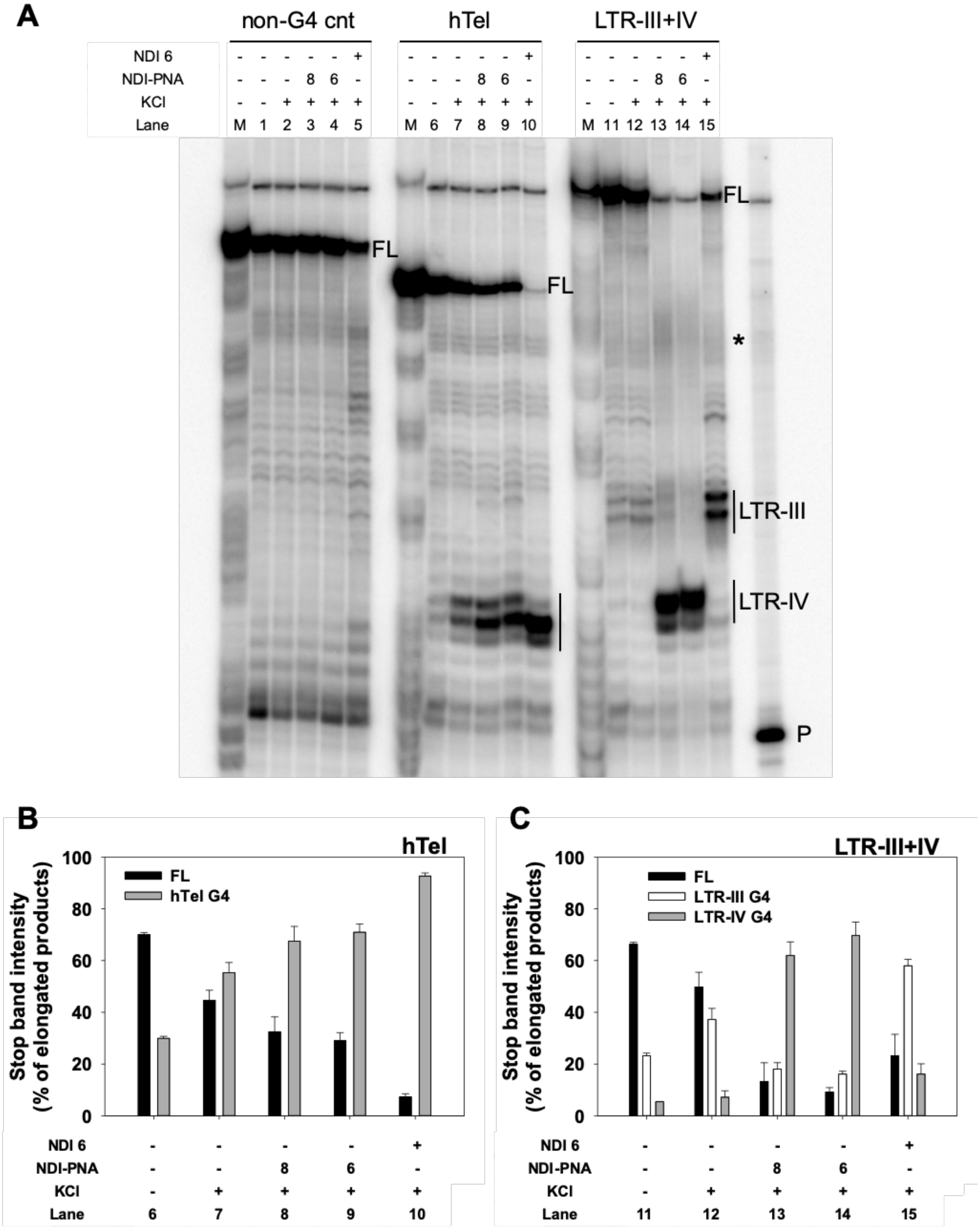
A
non-G4 cnt
hTel
LTR-III+IV
NDI 6
NDI-PNA
KCl
Lane
M 1 2 3 4 5
M 6 7 8 9 10
M 11 12 13 14 15
FL
FL
FL
*
LTR-III
LTR-IV
P
B
hTel
Stop band intensity (% of elongated products)
FL
hTel G4
C
LTR-III+IV
FL
LTR-III G4
LTR-IV G4
NDI 6
NDI-PNA
KCl
Lane
6 7 8 9 10
11 12 13 14 15

Figure S13. Image of a typical Taq polymerase stop assay. (**A**) LTR-III+IV and hTel templates were amplified by Taq polymerase at 42 °C in the absence (lanes 6 and 11) and presence of $1x10^{-1}$ M KCl, alone (lanes 7 and 12) or with $4x10^{-7}$ M of **NDI-PNA 8** (lanes 8 and 13), **NDI-PNA 6** (lanes 9 and 14) or NDI **6** (lanes 10 and 15). A template (non-G4 cnt) made of a scrambled sequence unable to fold into G4 was also used as negative control (lanes 1-5). Lane P: unreacted labelled primer. Lane M: ladder of markers obtained by the Maxam and Gilbert sequencing carried out on the amplified strand complementary to the template strand. Vertical bars indicate G4-specific Taq polymerase stop sites. * indicates the stop site corresponding to the binding of the PNA moiety to its complementary sequence on the LTR-III*IV template. (**B**) Quantification of lanes 6-10 shown in panel A. Quantification of stop bands corresponding to hTel G4 and of the full-length amplification product (FL) is shown. (**C**) Quantification of lanes 11-15 shown in panel A. Quantification of stop bands corresponding to LTR-III, LTR-IV G4s and of the full-length amplification product (FL) is shown.

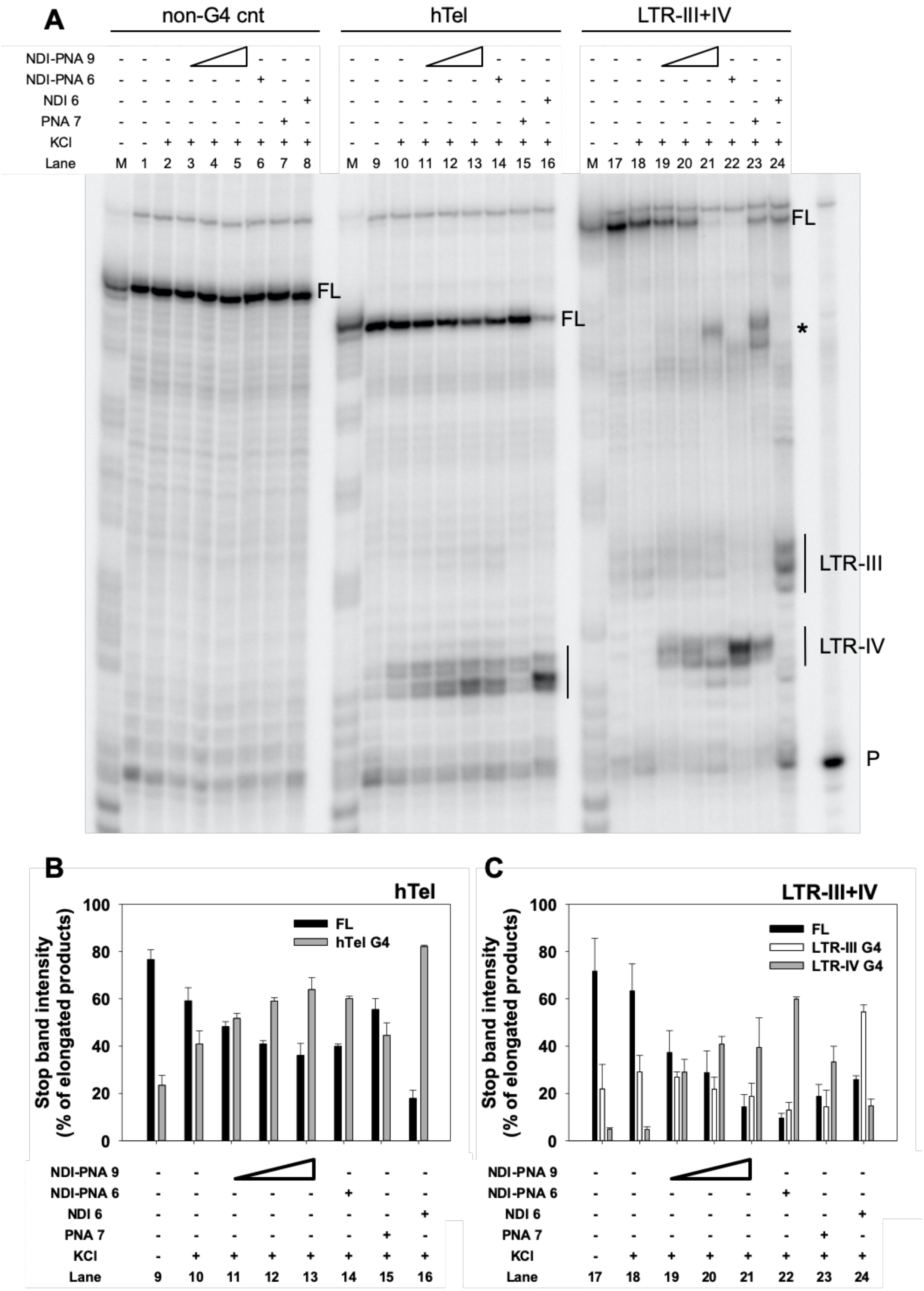
A
non-G4 cnt
hTel
LTR-III+IV
NDI-PNA 9
NDI-PNA 6
NDI 6
PNA 7
KCl
Lane
M 1 2 3 4 5 6 7 8
M 9 10 11 12 13 14 15 16
M 17 18 19 20 21 22 23 24
FL
FL
FL
*
LTR-III
LTR-IV
P
B
hTel
Stop band intensity
(% of elongated products)
0
20
40
60
80
100
FL
hTel G4
NDI-PNA 9
NDI-PNA 6
NDI 6
PNA 7
KCl
Lane
9 10 11 12 13 14 15 16
C
LTR-III+IV
Stop band intensity
(% of elongated products)
0
20
40
60
80
100
FL
LTR-III G4
LTR-IV G4
NDI-PNA 9
NDI-PNA 6
NDI 6
PNA 7
KCl
Lane
17 18 19 20 21 22 23 24

Figure S14. Image of a typical Taq polymerase stop assay. (**A**) LTR-III+IV and hTel templates were amplified by Taq polymerase at 42 °C in the absence (lanes 9 and 17) and presence of $1x10^{-1}$ M KCl, alone (lanes 10 and 18) or with increasing amounts ($1x10^{-7}$, $2x10^{-7}$ and $4x10^{-7}$ M) of **NDI-PNA 9** (lanes 11-13 and 19-21), $4x10^{-7}$ M of **NDI-PNA 6** (lanes 14 and 22), $4x10^{-7}$ M of **PNA 7** (lanes 15 and 23) or $4x10^{-7}$ M of NDI **6** (lanes 16 and 24). A template (non-G4 cnt) made of a scrambled sequence unable to fold into G4 was also used as negative control (lanes 1-8). Lane P: unreacted labelled primer. Lane M: ladder of markers obtained by the Maxam and Gilbert sequencing carried out on the amplified strand complementary to the template strand. Vertical bars indicate G4-specific Taq polymerase stop sites. * indicates the stop site corresponding to the binding of the PNA moiety to its complementary sequence on the LTR-III+IV template. (**B**) Quantification of lanes 9-16 shown in panel A. Quantification of stop bands corresponding to hTel G4 and of the full-length amplification product (FL) is shown. (**C**) Quantification of lanes 17-24 shown in panel A. Quantification of stop bands corresponding to LTR-III, LTR-IV G4s and of the full-length amplification product (FL) is shown.

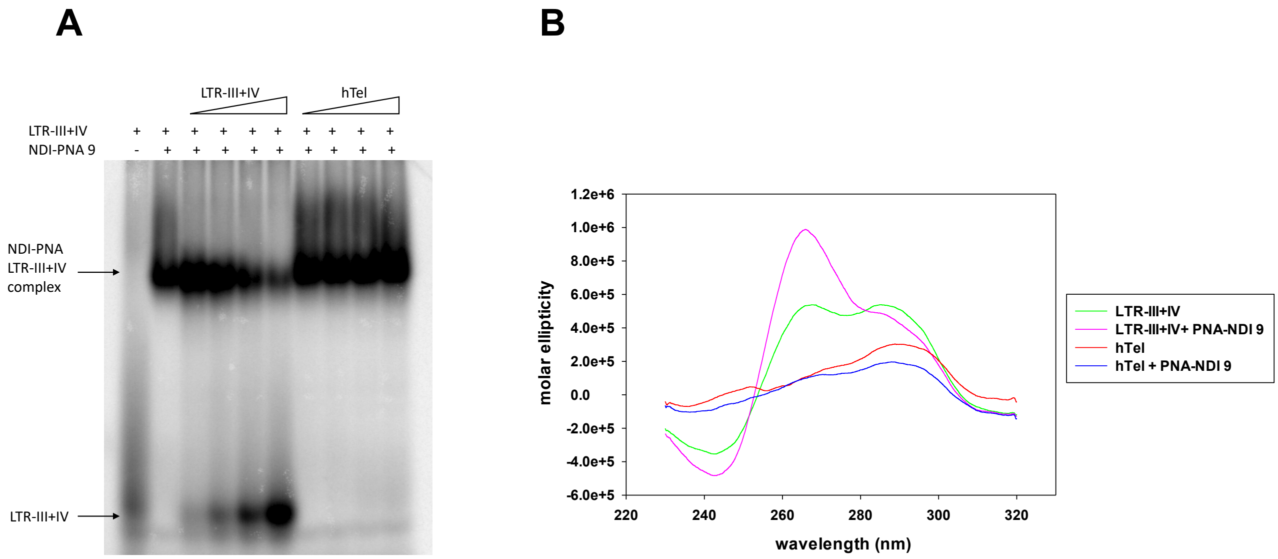

Figure S15. Biophysical characterization of **NDI-PNA 9**. **A)** EMSA competition analysis of the complex between LTR-III+IV and **NDI-PNA 9** with LTR-III+IV or hTel oligonucleotides. ^{32}P labeled LTR-III+IV (2.5×10^{-7} M) was incubated with **NDI-PNA 9** (2.5×10^{-7} M) alone or in the presence of increasing amounts of cold competitor LTR-III+IV or hTel oligonucleotides (0.25 – 0.5 – 1 – 2×10^{-6} M) in lithium cacodylate buffer (1×10^{-2} M, pH 7.4) in the presence of KCl (1×10^{-1} M) at 37° C for 24 hours. Samples were loaded on a 16% polyacrylamide native gel and run for 22 hours at 60 V. Gels were exposed overnight and visualized by phosphorimaging. On the left of the gel the position of the free labeled LTR-III+IV and that of the complex with **NDI-PNA 9** are indicated. **B)** Representative spectra of **NDI-PNA 9** with LTR-III+IV and hTel oligonucleotides.

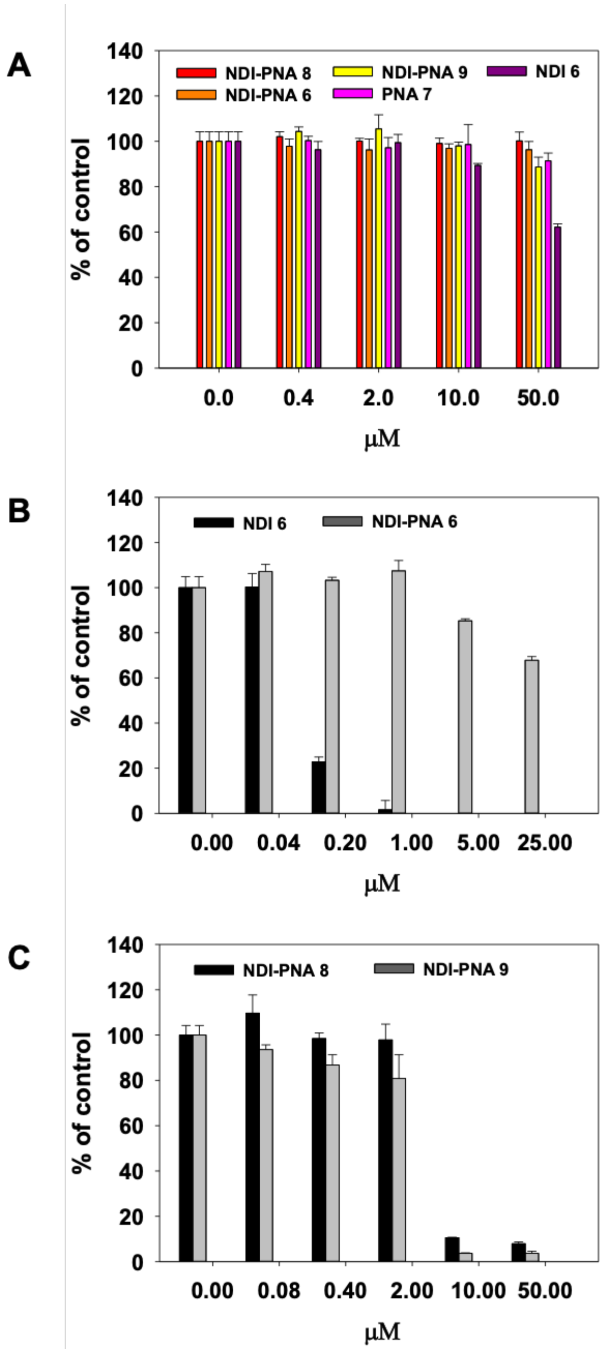

Figure S16. TZM-bl cells were treated with increasing concentrations of ligands and cell viability was evaluated by MTT assay (**A**) 3 h and (**B**, **C**) 48 h post treatment. The percentage of cell viability was calculated as follows: cell survival = (Awell − Ablank)/(Acontrol − Ablank) × 100, where blank denotes the medium without cells. Data are expressed as mean ± s.d. from two independent experiments.

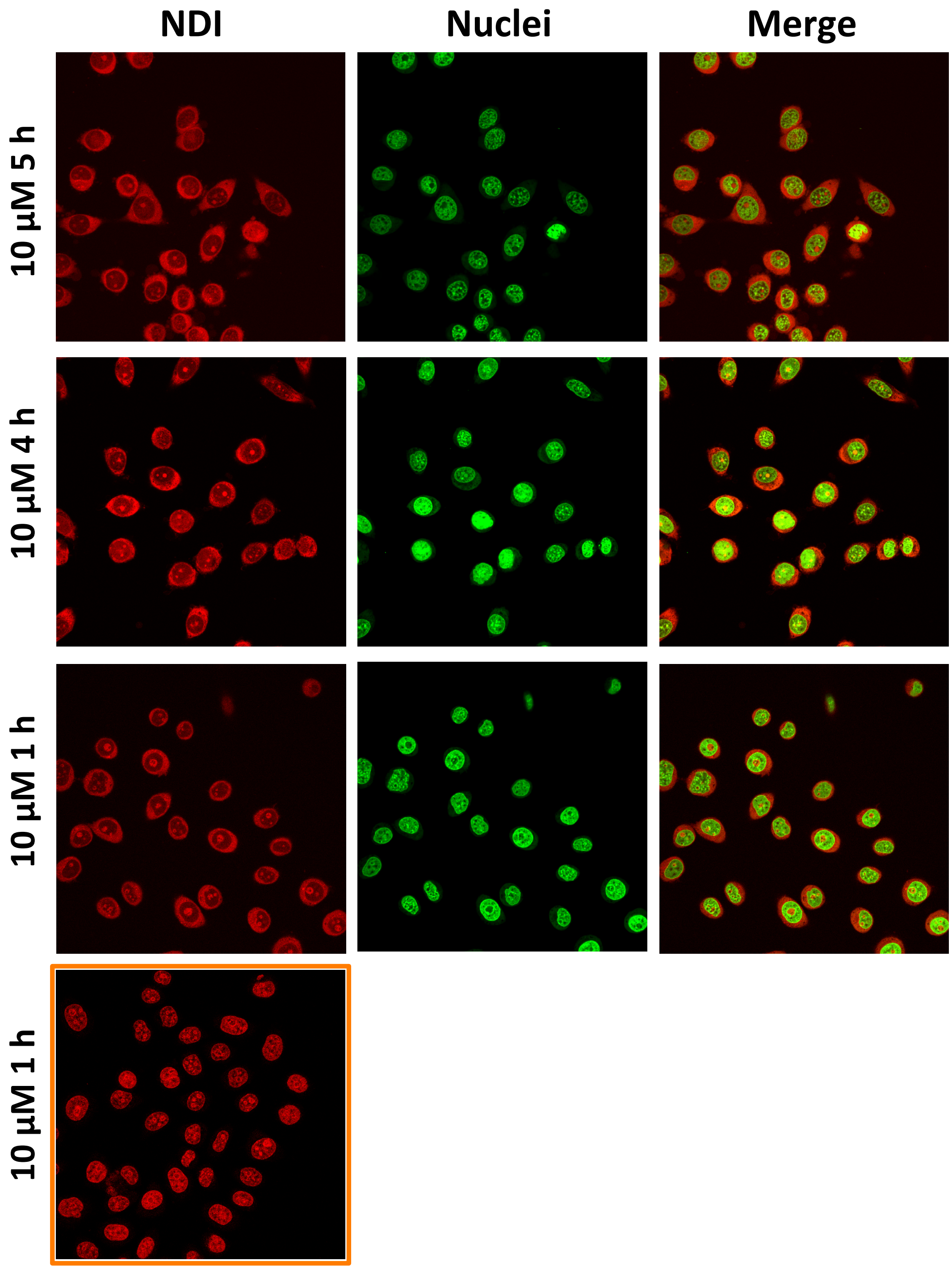

Figure S17. Images of TZM-bl cells treated with 1x10^{-5} M of **NDI-PNA 9** at different incubation times (1, 4, 5 h). For visualization, cells were fixed in 2% PFA treated with nuclear green LCS1 dye. Left panels: **NDI-PNA 9** distribution in the cells visualized at 561 nm excitation wavelength and 570-620 nm emission range; middle panels: cell nuclei visualized at 488 nm excitation wavelength and 500-550 nm emission range; right panels: overlap of NDI and nuclei localization. To note that nuclear green LCS1 dye expels the compound from the nucleus to the cytoplasm, as shown by the bottom left orange-framed panel: TZM-bl cells treated with 1x10^{-5} M of **NDI-PNA 9** and visualized without previous treatment with nuclear green LCS1 dye**.**

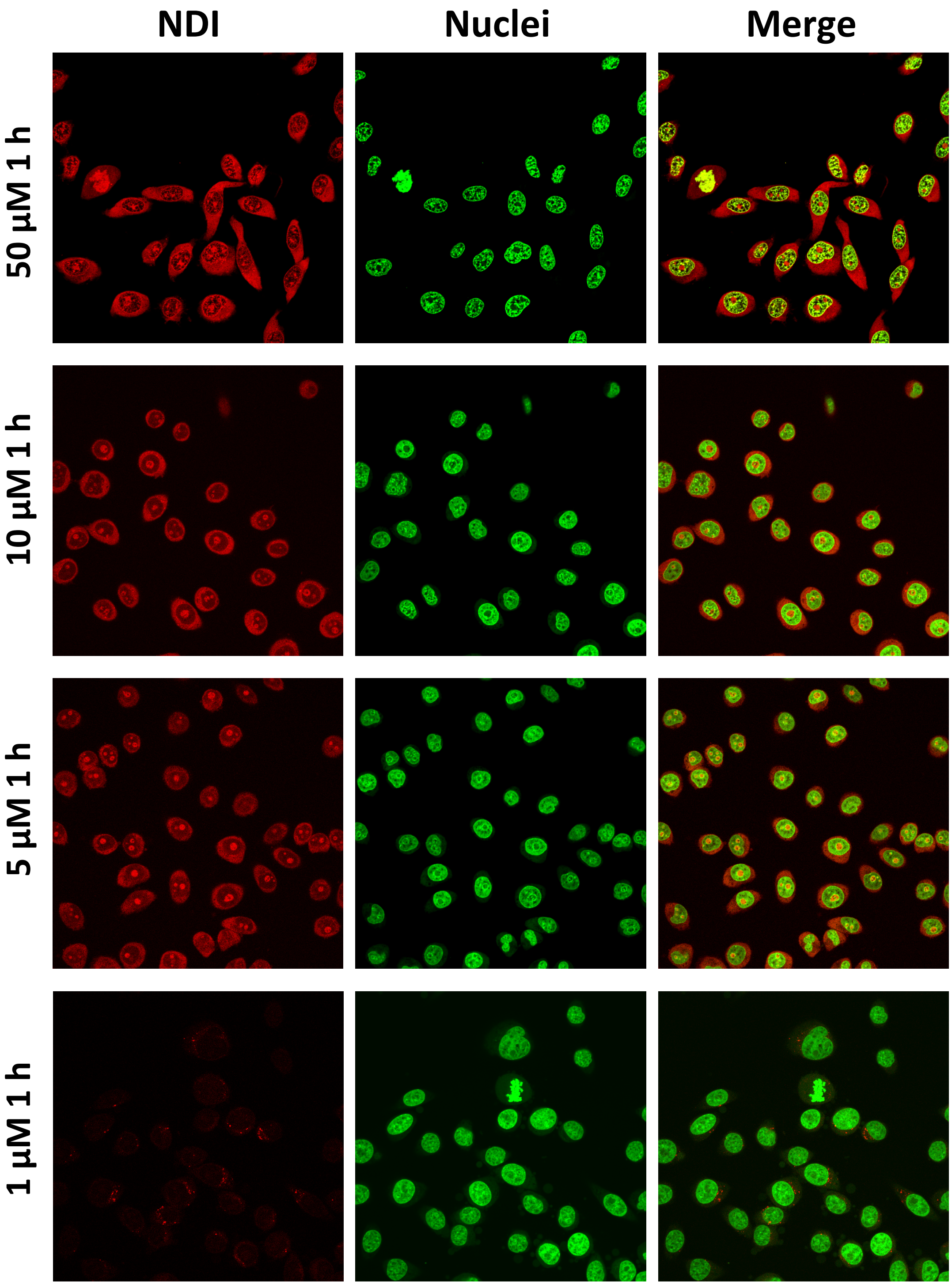

Figure S18. Images of TZM-bl cells treated with different concentrations of **NDI-PNA 9** (1-50x10^{-6} M) for 1 h. For visualization, cells were fixed in 2% PFA treated with nuclear green LCS1 dye. Left panels: **NDI-PNA 9** distribution in the cells visualized at 561 nm excitation wavelength and 570-620 nm emission range; middle panels: cell nuclei visualized at 488 nm excitation wavelength and 500-550 nm emission range; right panels: merge of NDI and nuclei localization. To note that nuclear green LCS1 dye expels the compound from the nucleus to the cytoplasm, as shown by the bottom left orange-framed panel in Figure S16.

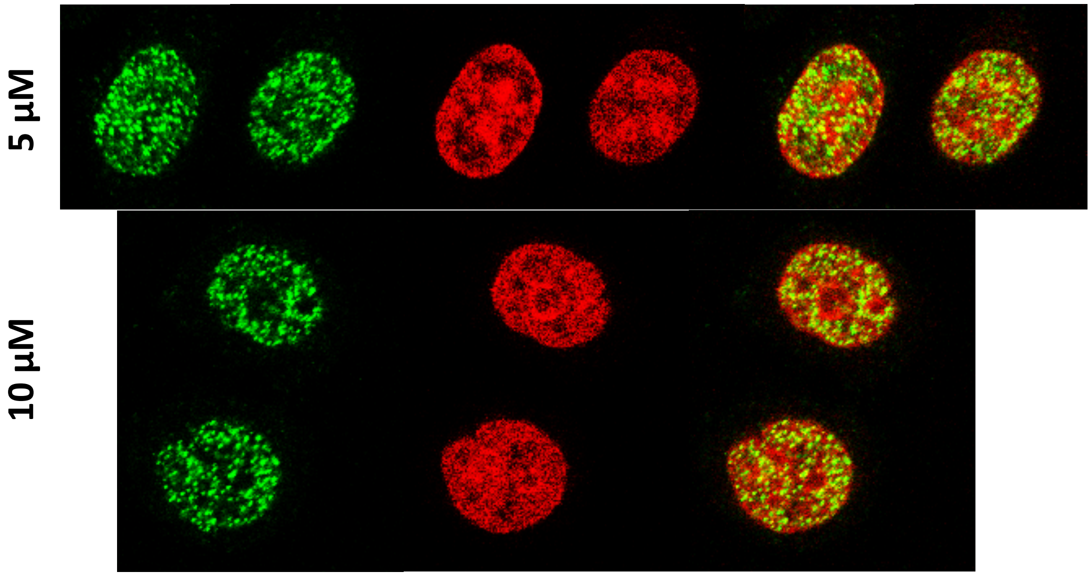

Figure S19. Colocalization of **NDI-PNA 9** with the anti-G4 antibody 1H6. **NDI-PNA 9** was used at the indicated concentrations and incubated for 1 h. Left panels: G4 localization (green signal) visualized at 488 nm excitation wavelength and 500-550 nm emission range; middle panels: **NDI-PNA 9** localization (red signal) visualized at 561 nm excitation wavelength and 570-620 nm emission range; right panels: overlapping of G4s and **NDI-PNA 9** (yellow signal). Cells were incubated with the compound for 1 h before cell fixation in 2% PFA, permeabilization with 0.5% Tween-20 for 40 min at r.t., blocking with BlockAid for 1 h at 37 °C, incubation with 1 µg/mL anti-G4 antibody 1H6 for 2 h at r.t. and with 1:250 Alexa 488 anti-mouse IgG antibody for 1 h at 37 °C.

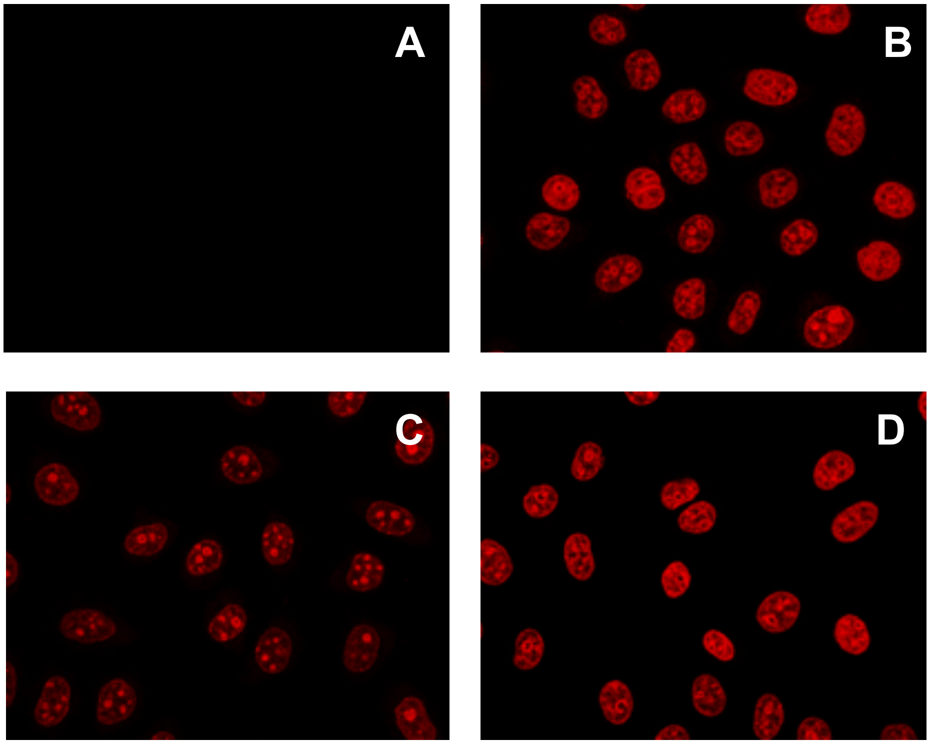

Figure S20. Images of (**A**) untreated TZM-bl cells, TZM-Bl cells treated with 1x10^{-5} M of **NDI-PNA 9**, alone (**B**) or in the presence of (**C**) 200 units DNase I for 30 min at 37 °C (**D**) or 40 μg/mL RNaseA for 30 min at 37 °C. The compounds were incubated with the cells for 1 h before cell fixation in 2% PFA. For NDI/conjugates images were visualized at 561 nm excitation wavelength and 570-620 nm emission range.

HPLC purity data

Compound **2**

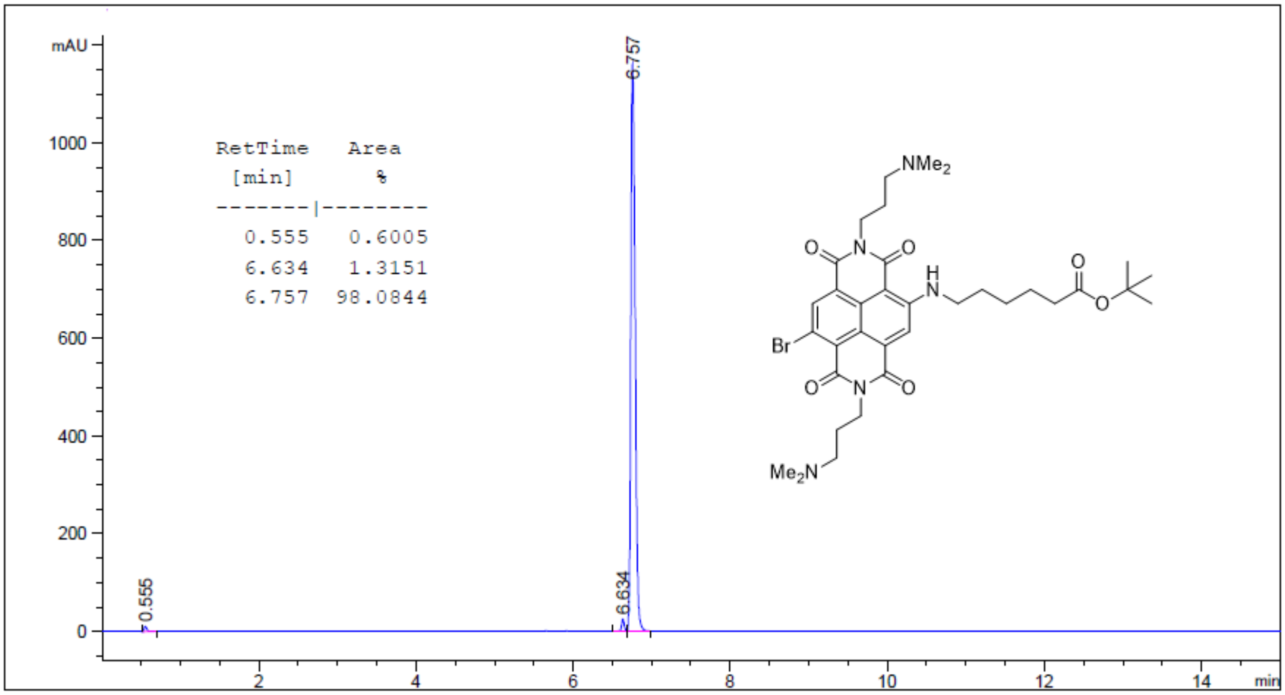

Compound **3**

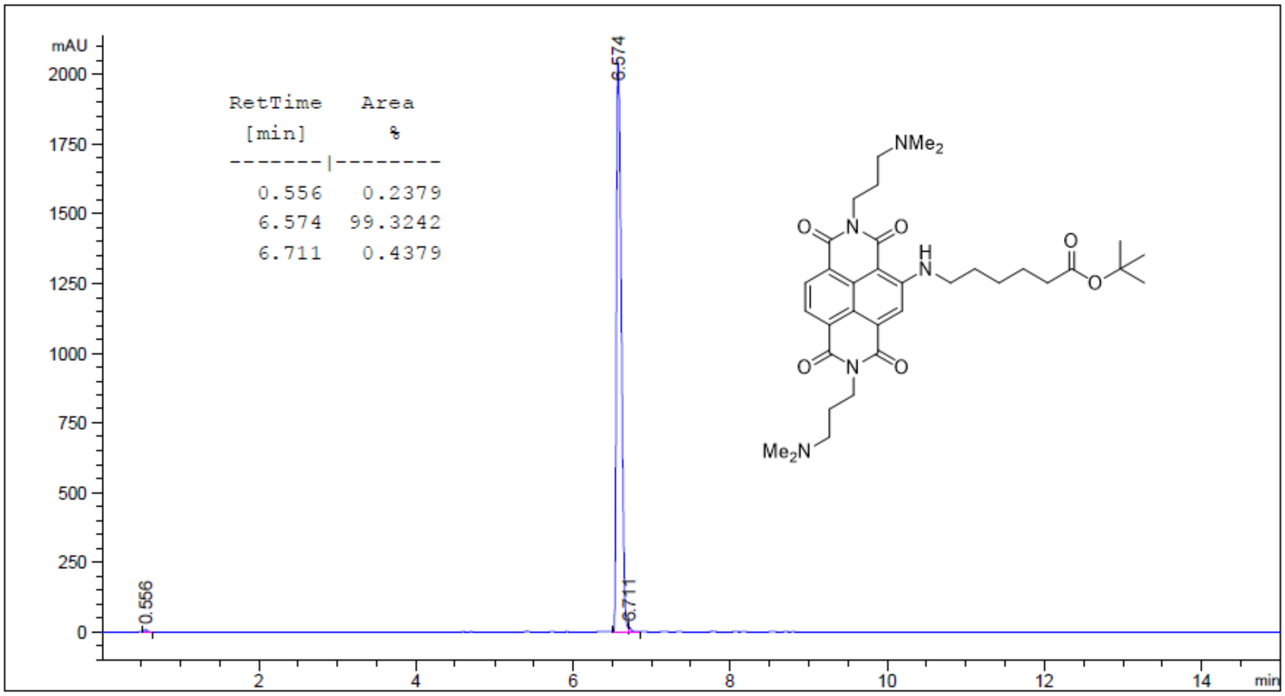

Compound **4**

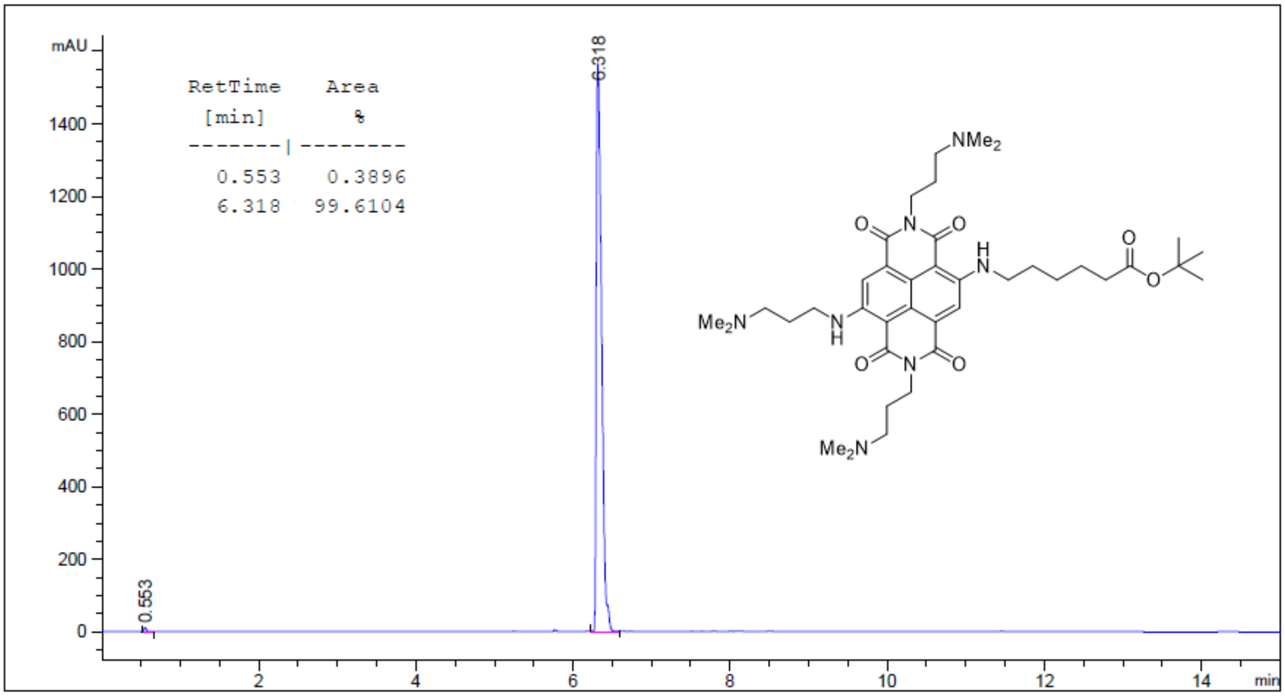

Compound **5**

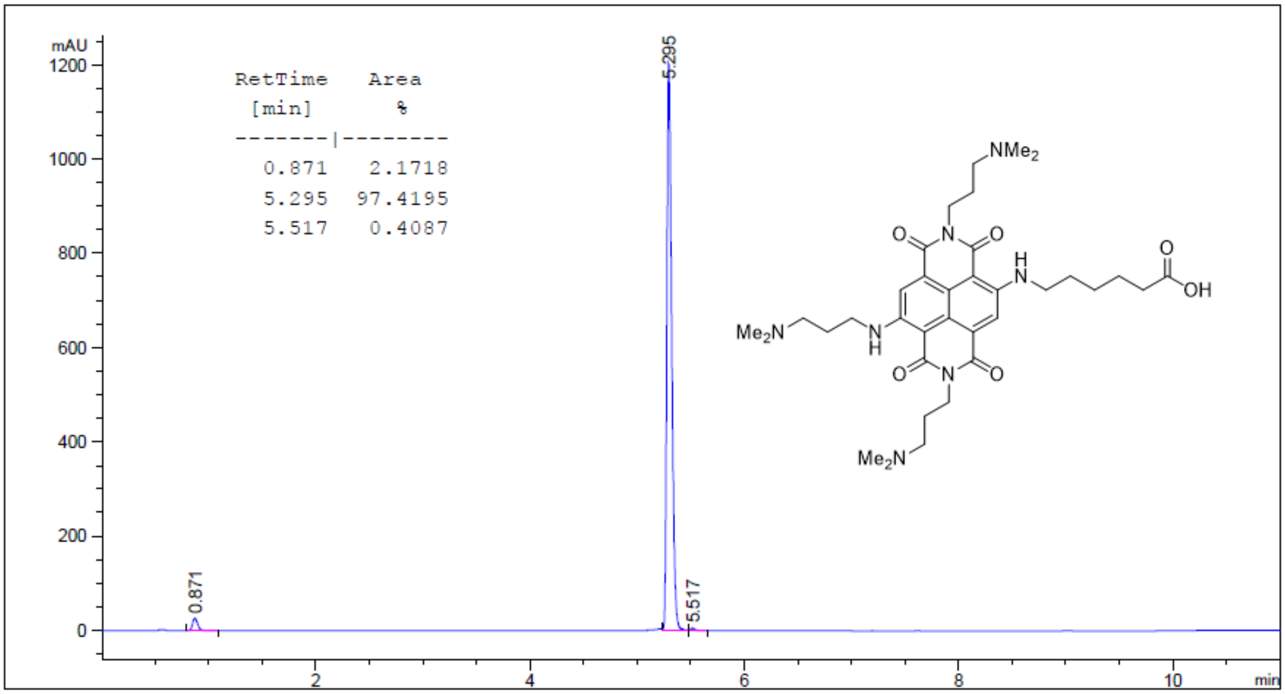

NMR characterization

Compound **2**

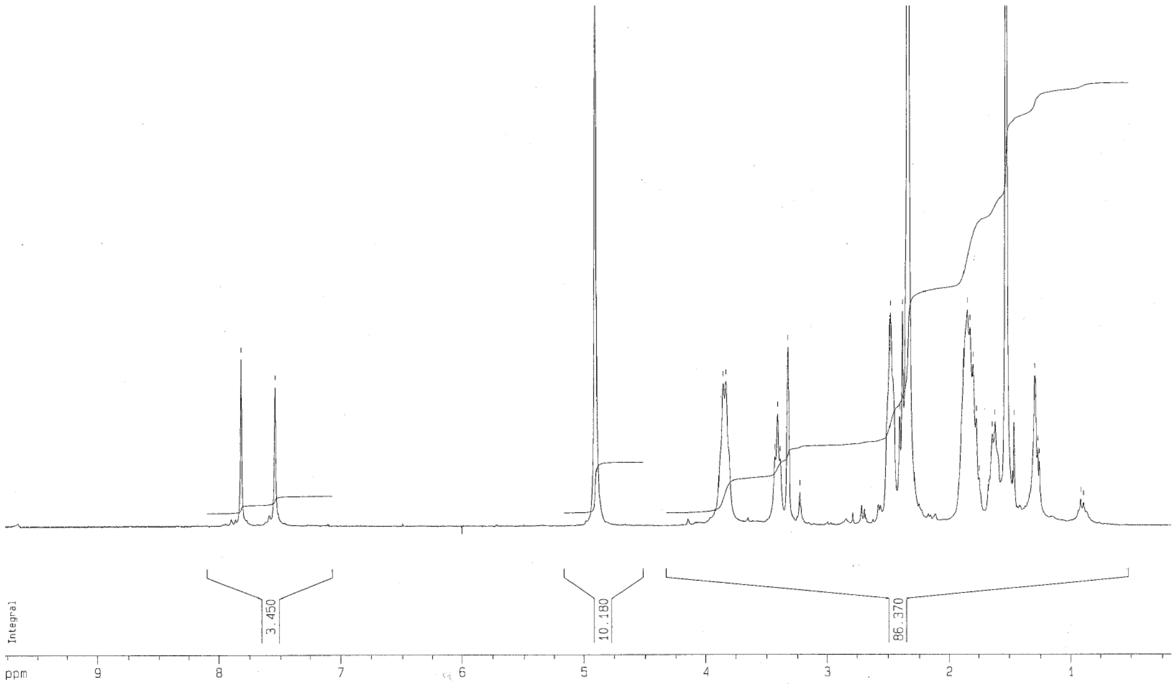

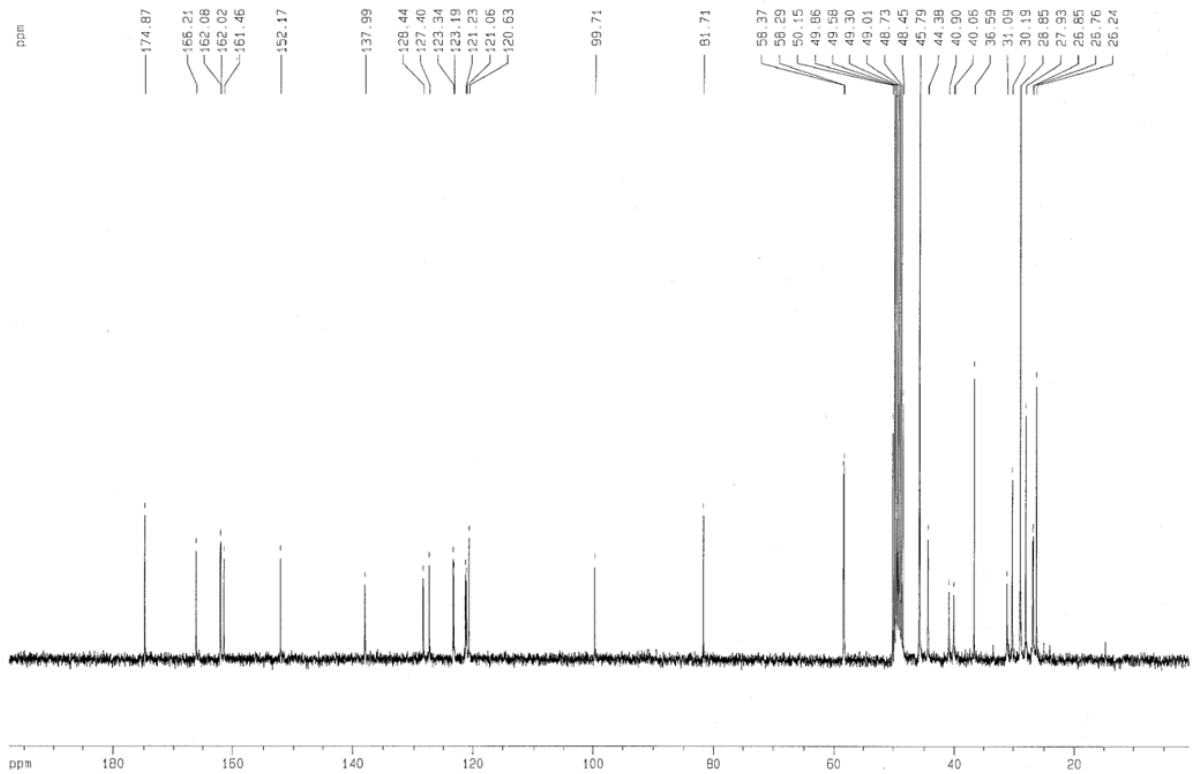
ppm
174.87
166.21
162.08
162.02
161.46
152.17
137.99
128.44
127.40
123.34
123.19
121.23
121.06
120.63
99.71
81.71
58.37
58.29
50.15
49.86
49.58
49.30
49.01
48.73
48.45
45.79
44.38
40.90
40.06
36.59
31.09
30.19
28.85
27.93
26.85
26.76
26.24
ppm 180 160 140 120 100 80 60 40 20

Compound **3**

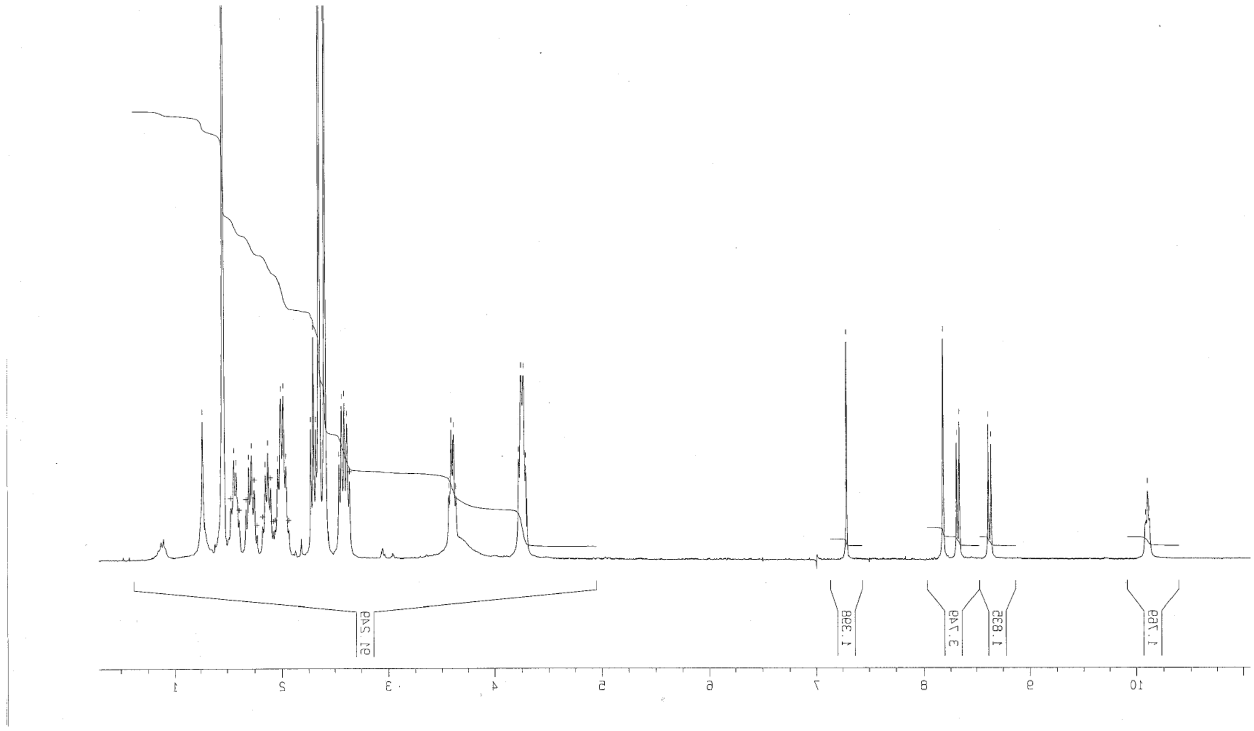

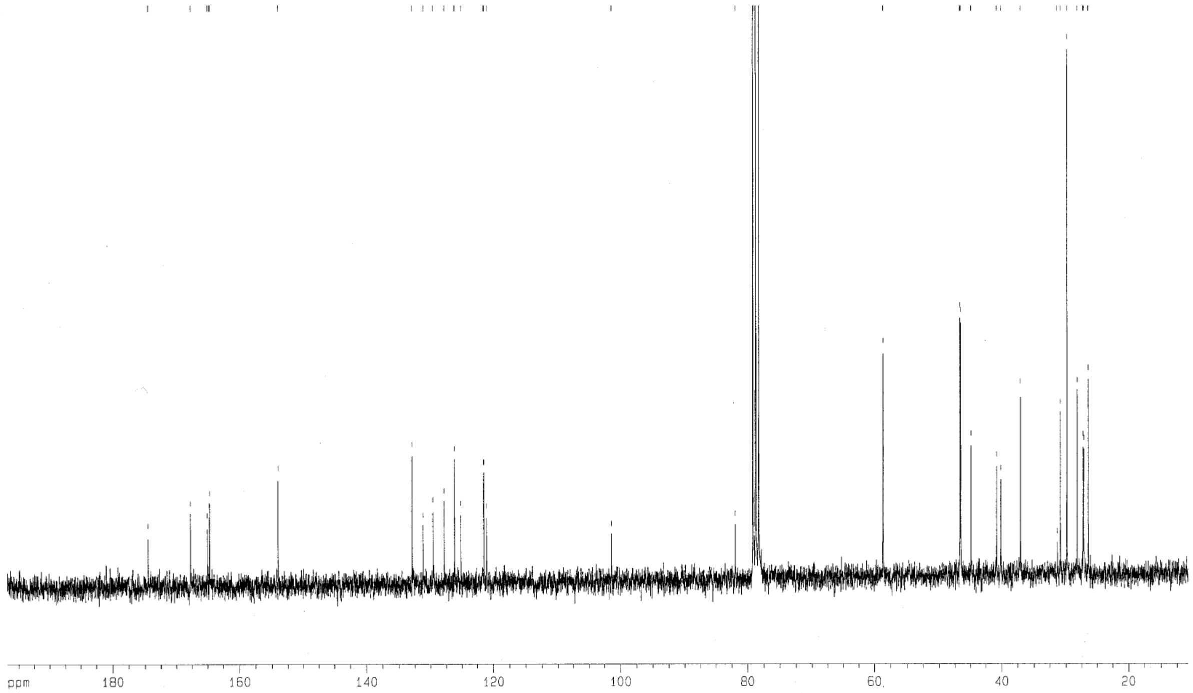
ppm
180
160
140
120
100
80
60
40
20

Compound **4**

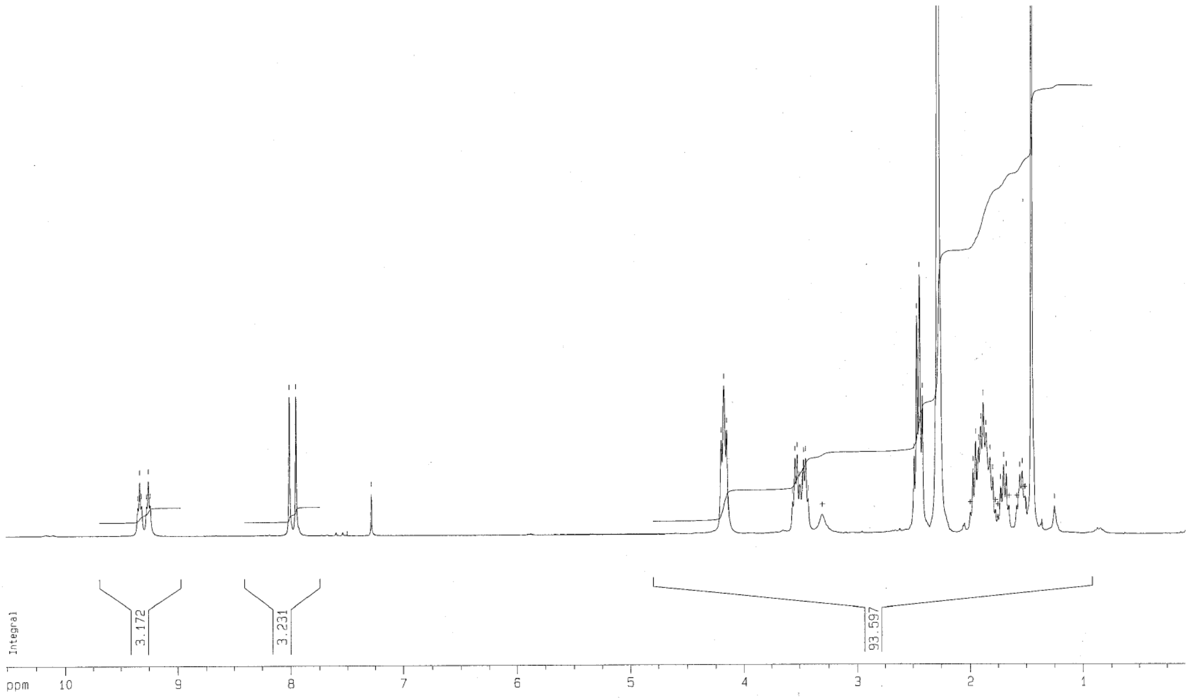

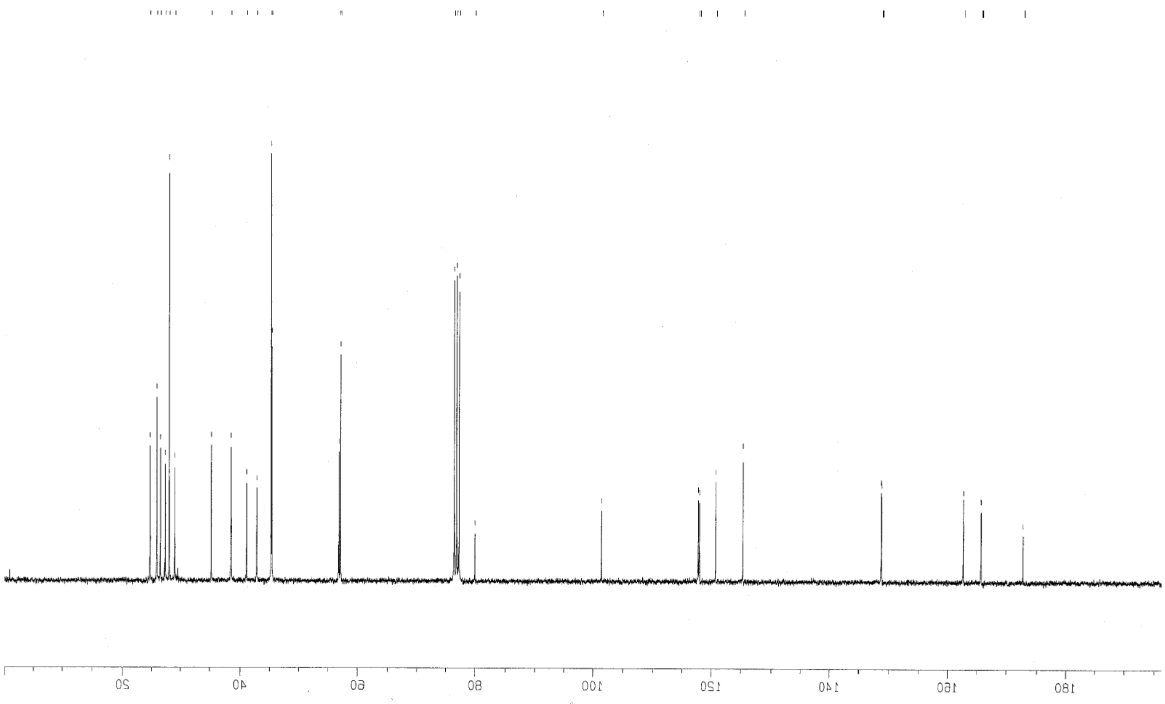
20
40
60
80
100
120
140
160
180

Compound **5**

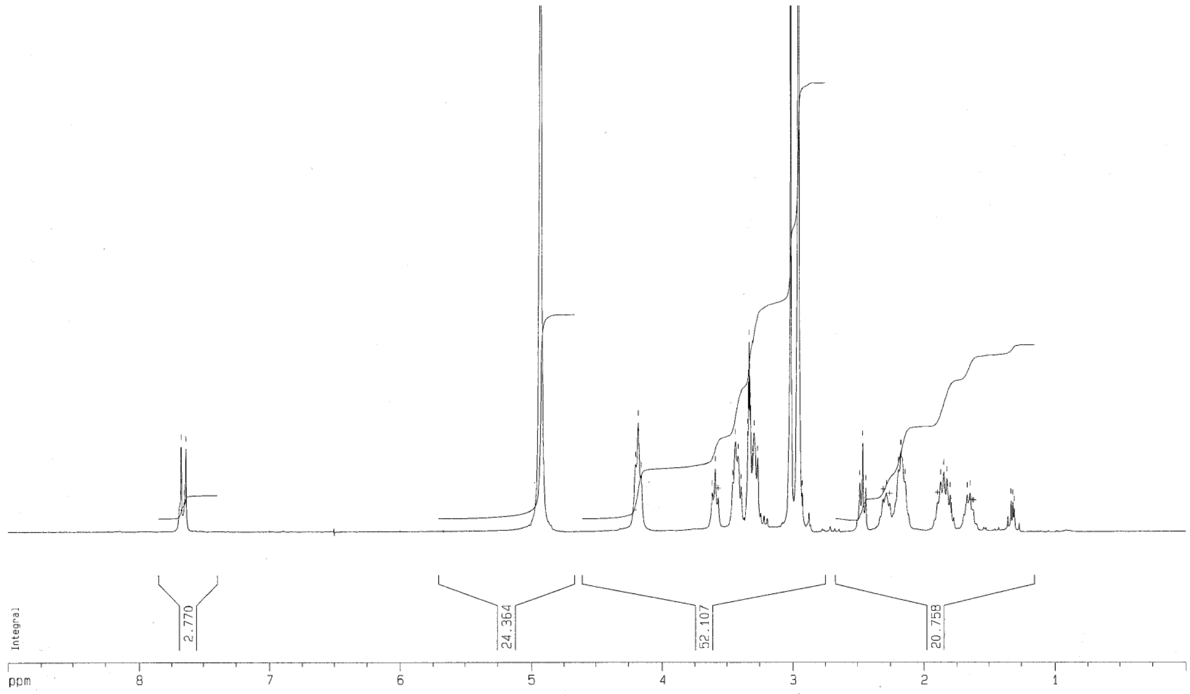

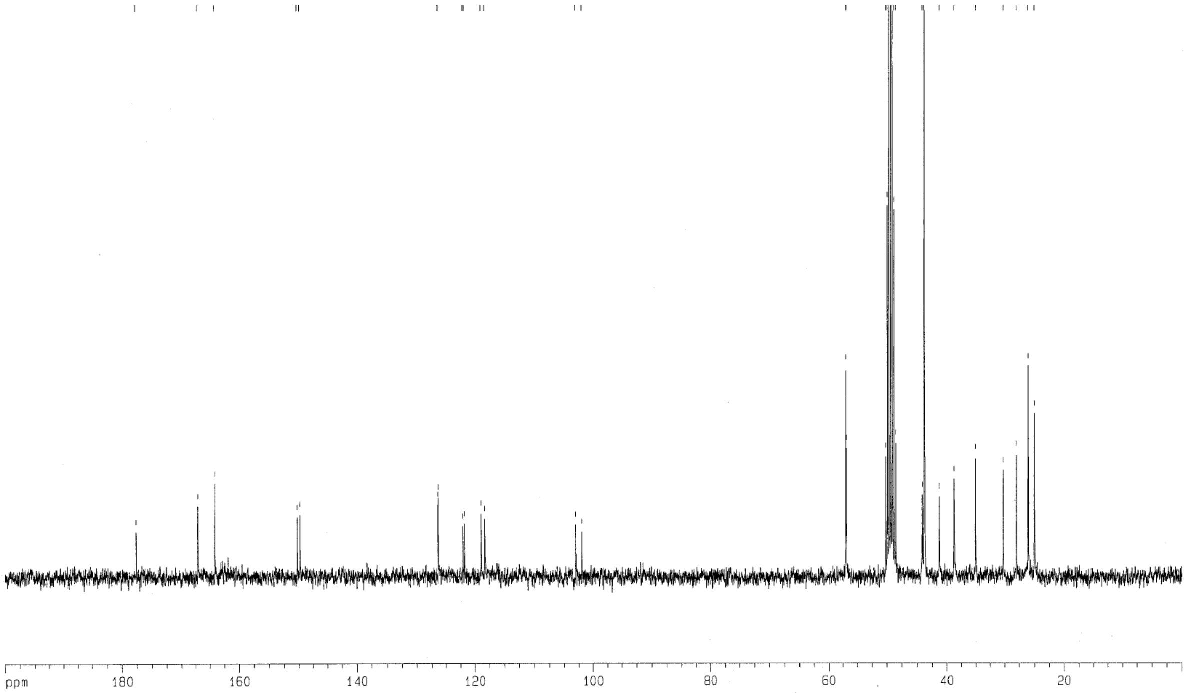
ppm
180
160
140
120
100
80
60
40
20

ESI-MS characterization

NDI-PNA 1

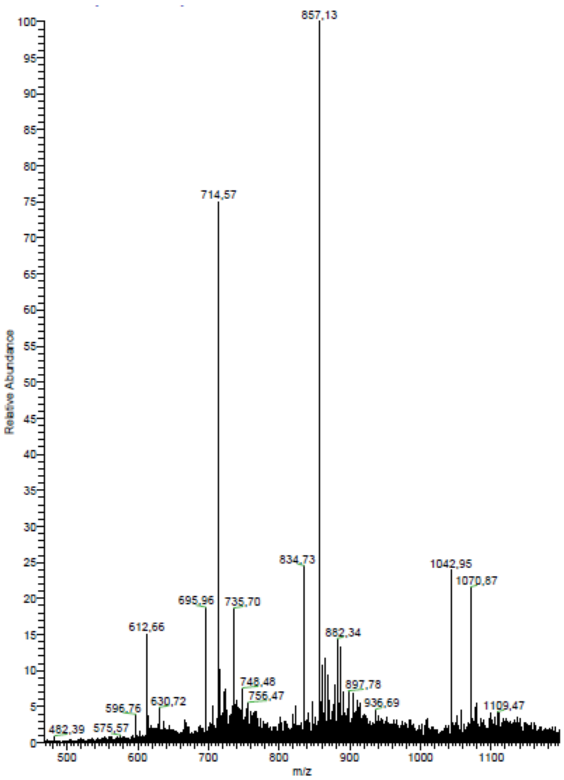

NDI-PNA 2

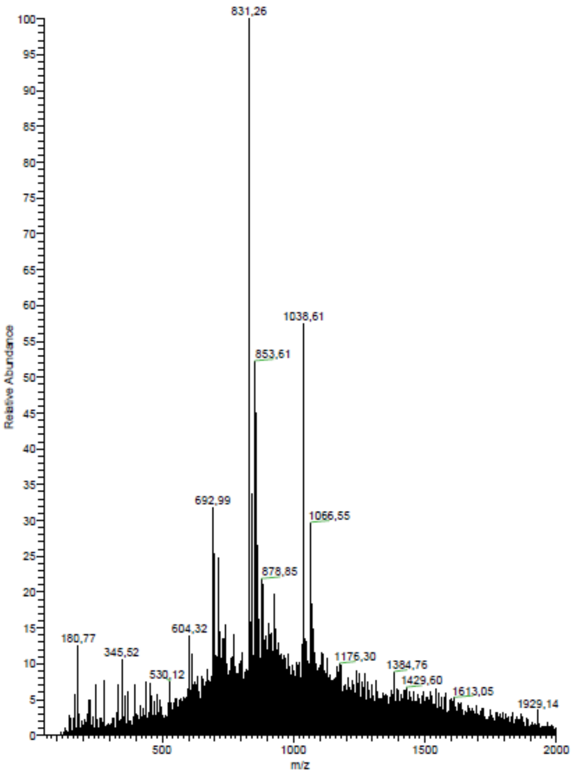

NDI-PNA 3

NDI-PNA 4

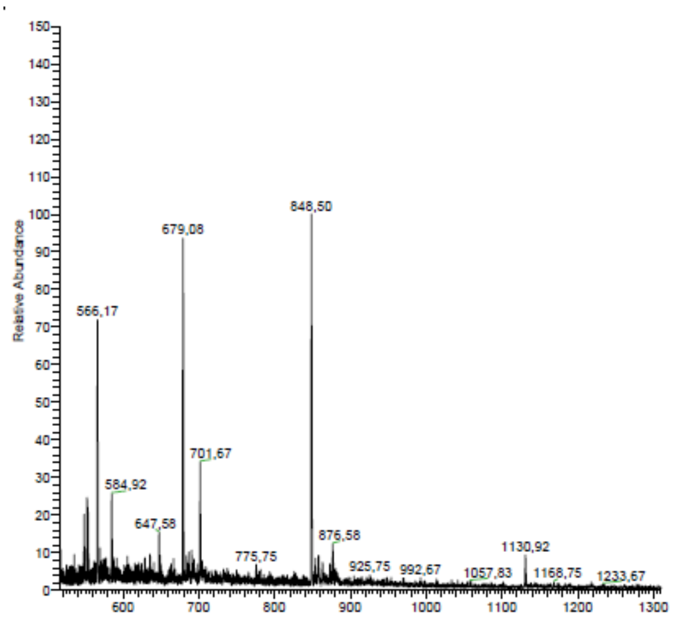

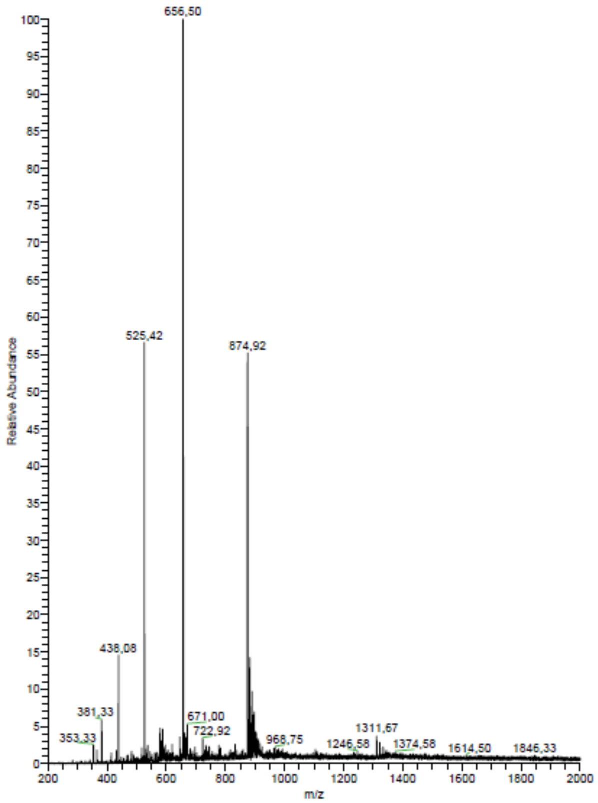

NDI-PNA 5

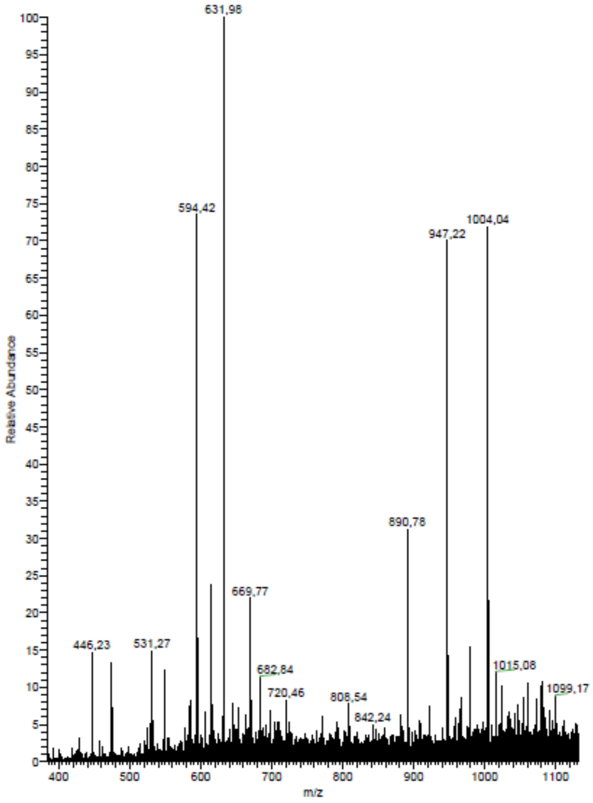

NDI-PNA 6

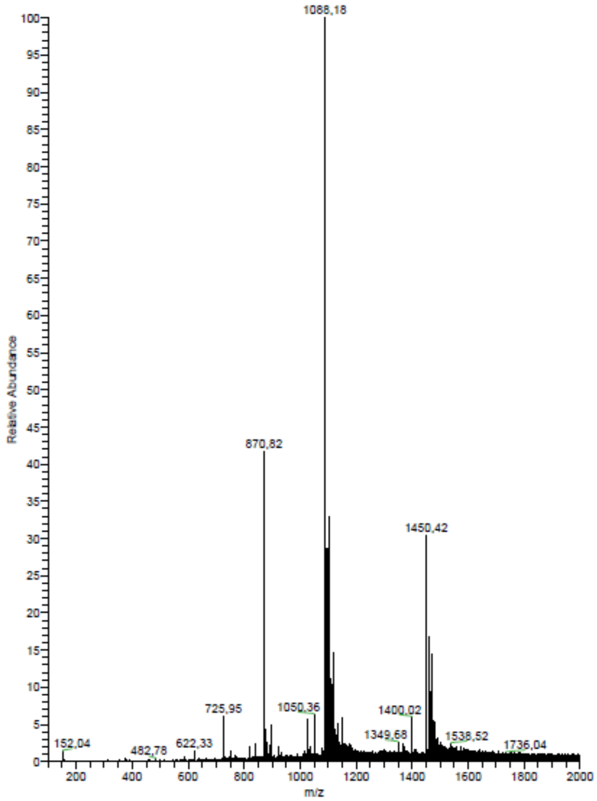

PNA 7

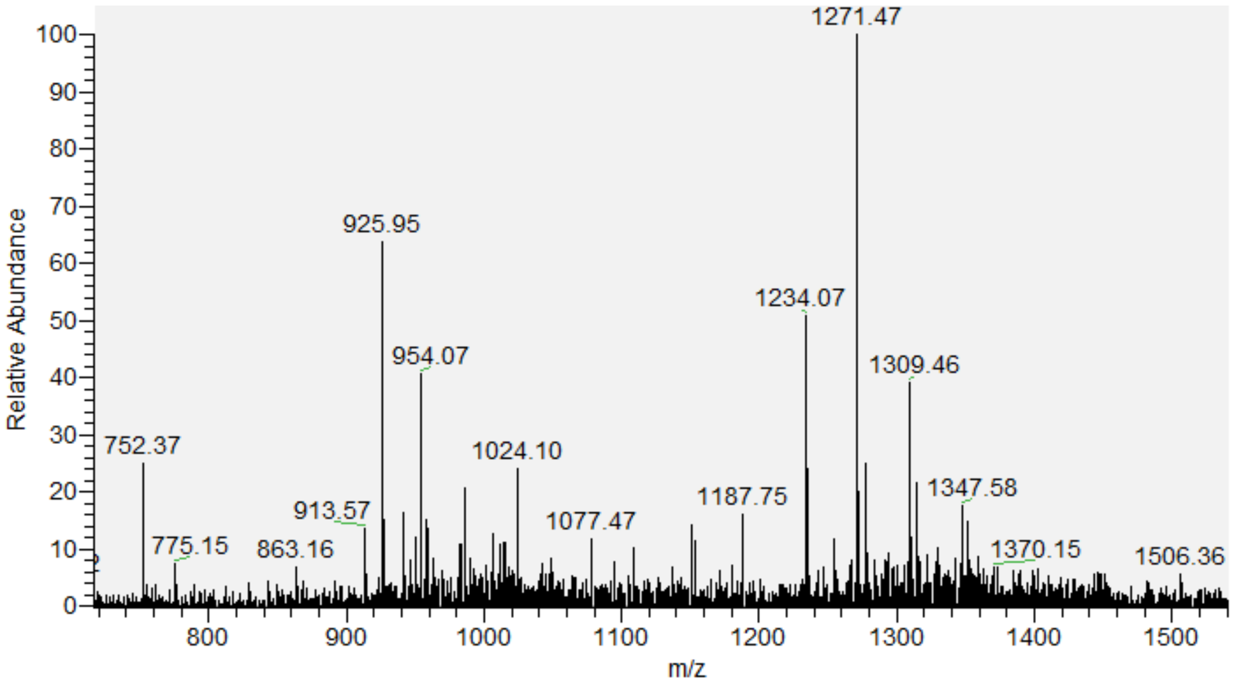

NDI-PNA 8

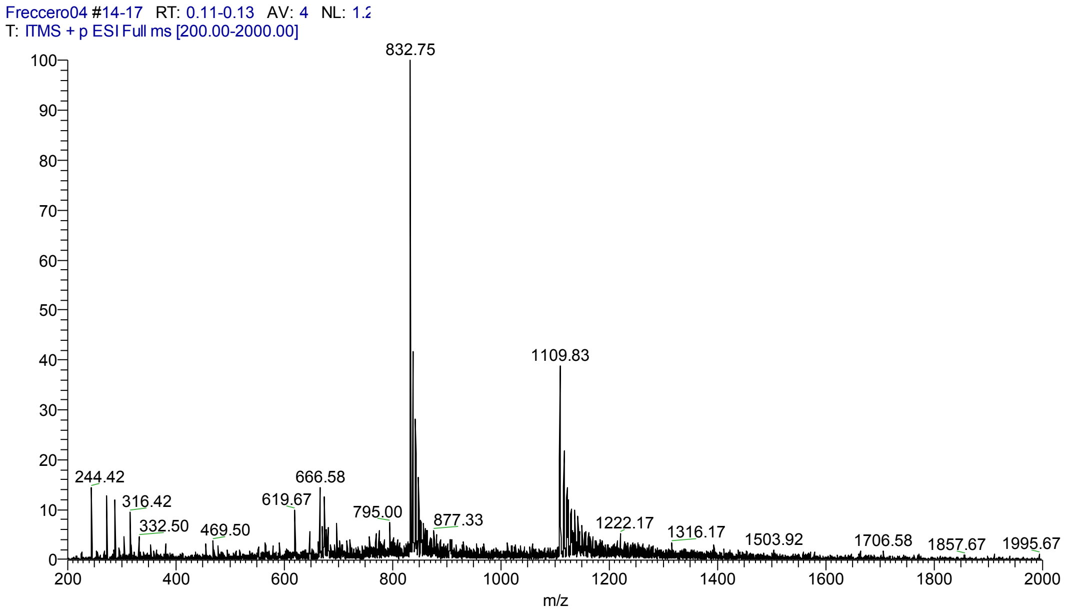

NDI-PNA 9

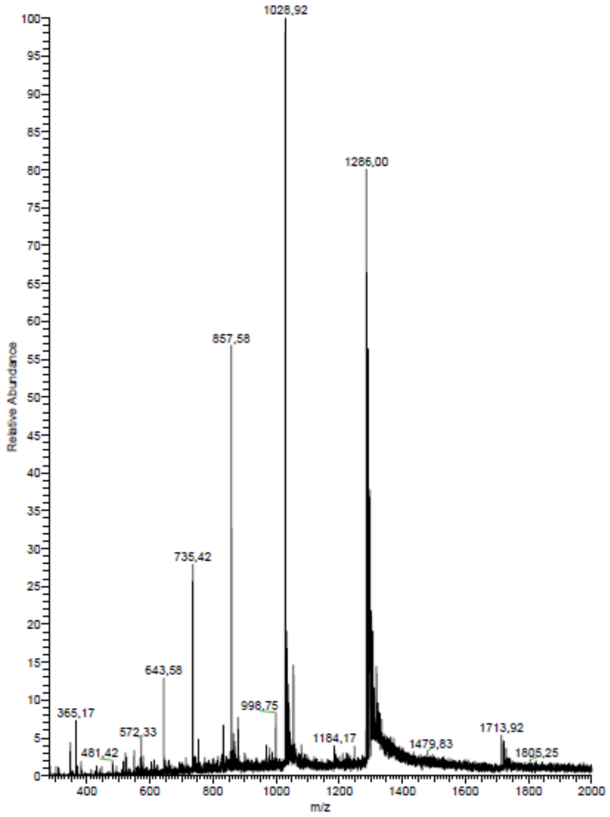
1028,92
1286,00
857,58
735,42
643,58
998,75
365,17
572,33
481,42
1184,17
1479,83
1713,92
1805,25
Relative Abundance
m/z
